# GM‐CSF Promotes Neutrophil PD‐L1 Expression Through the VEGFR‐2/STAT6/*CSF2* Signaling Axis in Oral Squamous Cell Carcinoma

**DOI:** 10.1002/advs.76960

**Published:** 2026-08-06

**Authors:** Fangxing Zhu, Yuanhe You, Yiyi Zhang, Yibin Dai, Xinyu Zhou, Yong Fu, Xi Lu, Yingying Huang, Zhihang Zhou, Ronghui Xia, Wutong Ju, Zhaoqi Yuan, Laiping Zhong, Tongchao Zhao

**Affiliations:** ^1^ Oromaxillofacial Head and Neck Surgery Center for Oromaxillofacial Head and Neck Disorders Huashan Hospital Fudan University Shanghai China; ^2^ Department of Oral & Maxillofacial‐Head & Neck Oncology Shanghai Ninth People's Hospital Shanghai Jiao Tong University School of Medicine Shanghai China; ^3^ College of Stomatology Shanghai Jiao Tong University Shanghai China; ^4^ National Center for Stomatology National Clinical Research Center for Oral Diseases Shanghai China; ^5^ Shanghai Key Laboratory of Stomatology Shanghai Research Institute of Stomatology Shanghai China; ^6^ Department of Oral Pathology Shanghai Ninth People's Hospital Shanghai Jiao Tong University School of Medicine Shanghai China; ^7^ Nanjing Stomatological Hospital Affiliated Hospital of Medical School Institute of Stomatology Nanjing University Nanjing China; ^8^ Department of Endodontics Shanghai Ninth People's Hospital Shanghai Jiao Tong University School of Medicine Shanghai China; ^9^ Department of Plastic and Reconstructive Surgery Shanghai Ninth People's Hospital Shanghai Jiao Tong University School of Medicine Shanghai China; ^10^ Department of Oral & Maxillofacial‐Head & Neck Oncology Shanghai Second People's Hospital Shanghai China

**Keywords:** GM‐CSF, neutrophils, OSCC, STAT6, VEGFR‐2

## Abstract

Oral squamous cell carcinoma (OSCC) remains a highly aggressive malignancy with limited responsiveness to anti‐PD‐1 immunotherapy. Recent neoadjuvant studies combining VEGFR‐2 inhibition (VEGFR2i) with anti‐PD‐1 (aPD‐1) have yielded markedly improved pathological responses, suggesting a VEGFR‐2‐dependent immunoregulatory mechanism. Here, we identify a PD‐L1‐expressing CD66b^+^ neutrophil‐enriched tumor‐associated population as a critical immunosuppressive population enriched in VEGFR‐2‐high OSCC tumors and associated with increased metastatic propensity and inferior patient outcomes. Using multiplex immunohistochemistry, scRNA‐seq, and flow cytometry, we demonstrate that VEGFR‐2‐overexpressing OSCC cells drive robust GM‐CSF secretion, which in turn induces PD‐L1 upregulation in neutrophils. Mechanistically, VEGFR‐2 activation promotes STAT6 phosphorylation and nuclear translocation, enabling direct transcriptional activation of *CSF2*. GM‐CSF subsequently activates STAT5 and mTOR/S6K signaling in neutrophils, thereby enhancing PD‐L1 protein synthesis. Functionally, these PD‐L1^+^ TANs suppress CD8^+^ T‐cell proliferation, augment T‐cell exhaustion, and diminish cytotoxicity. Importantly, VEGFR‐2 inhibition or GM‐CSF neutralization restored CD8^+^ T‐cell function and improved responsiveness to anti‐PD‐1 therapy in vivo, whereas exogenous GM‐CSF impaired VEGFR2i/aPD‐1 efficacy. Collectively, our findings reveal a previously unrecognized VEGFR‐2/STAT6/*CSF2* axis that licenses neutrophil‐mediated immune suppression in OSCC. These data provide mechanistic rationale and preclinical support for targeting the VEGFR‐2/GM‐CSF‐neutrophil axis to improve the efficacy of PD‐1 blockade in OSCC.

## Graphical abstract

VEGFR‐2 signaling in OSCC activates Src–STAT6‐dependent CSF2 transcription, driving tumor‐derived GM‐CSF secretion. GM‐CSF programs neutrophils to express PD‐L1 through STAT5–mTOR/S6K signaling, suppressing cytotoxic CD8^+^ T cells. This pathway reveals a tumor–neutrophil immune checkpoint circuit that limits anti‐PD‐1 responsiveness in OSCC.

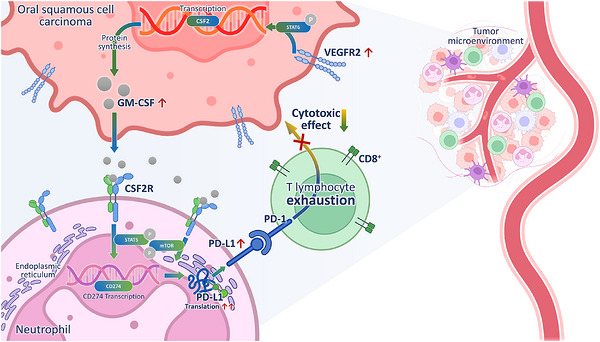

## 1 Introduction

Oral squamous cell carcinoma (OSCC) is the most common oral malignancy [1], with approximately 389 485 new cases and nearly 188 230 deaths worldwide in 2022 [2]. Standard management involves radical surgery followed by radiotherapy, as per the National Comprehensive Cancer Network (NCCN) guidelines [3]. However, this aggressive approach often compromises oral and maxillofacial function, appearance, and quality of life. Given that the five‐year survival rate remains approximately 50% [4], more effective and better individualized treatment strategies are urgently needed. We previously reported the phase I IceMelting 1.0 trial (NCT04393506), which demonstrated the safety and promising major pathological response (MPR) rate of neoadjuvant camrelizumab (anti‐PD‐1) combined with apatinib (VEGFR‐2 inhibitor, VEGFR2i) in patients with locally advanced resectable OSCC (LAROSCC) [5]. This response was superior to that reported for mono‐immunotherapy [6]. To explore the underlying mechanism, we first evaluated the antiangiogenic effect by immunofluorescence. Although CD31 and α‐SMA fluorescence intensity, representing tumor vasculature, was reduced after neoadjuvant therapy across the cohort, no difference was observed between patients who achieved MPR and those who did not. These findings suggested that VEGFR2i may exert an immunomodulatory effect beyond vascular normalization. We therefore performed multiplex immunohistochemistry (mIHC) to characterize the tumor immune microenvironment (TIME). Although several immune alterations were observed after treatment, including changes in CD68^+^CD163^+^ macrophages, CD3^+^ T cells, and the CD8^+^/FoxP3^+^ ratio, the mechanism by which VEGFR2i contributes to immune activation remained unclear.

PD‐L1 was initially identified as an immunosuppressive molecule expressed by tumor cells [7] and was later found on myeloid dendritic cells [8]. Based on the KEYNOTE‐048 and KEYNOTE‐689 studies, PD‐L1 combined positive score (CPS) is commonly used to identify patients who are more likely to benefit from immunotherapy and to predict clinical outcomes [9, 10]. CPS is calculated by dividing the number of PD‐L1‐stained cells, including tumor cells, lymphocytes, and macrophages, by the total number of viable tumor cells, and multiplying the result by 100 [11]. Notably, neutrophils and plasma cells are not included in PD‐L1 CPS calculation.

Neutrophils account for approximately 50%–70% of circulating leukocytes and represent the most abundant immune cell population in peripheral blood. Although traditionally considered short‐lived defensive leukocytes, neutrophils exhibit rapid and highly plastic responses to environmental cues. This plasticity may underlie their context‐dependent and sometimes opposing functions during inflammation and cancer. Within the tumor microenvironment (TME), tissue‐derived signals can rapidly reshape neutrophil phenotypes and functions [12]. With the rise of single‐cell RNA sequencing (scRNA‐seq), increasing attention has been paid to intratumoral neutrophils, also referred to as tumor‐associated neutrophils (TANs) [13]. TANs display considerable heterogeneity across tumor types and may exert either protumor or antitumor functions depending on the microenvironment. Large‐scale scRNA‐seq analysis has identified PD‐L1^+^ TANs in the liver tumor immune microenvironment, where they suppress T‐cell cytotoxicity [14]. In lung cancer, CD40 agonist antibody therapy acutely expands tumor neutrophils, predominantly comprising Sell^high^ neutrophils [15]. In pancreatic ductal adenocarcinoma, intratumoral neutrophils converge into a distinct transcriptional state characterized by hypoxia, glycolysis, and angiogenesis signatures that are not chromatin‐accessible in bone marrow, spleen, or peripheral blood neutrophils [16]. However, the role of neutrophils in the TIME of LAROSCC remains insufficiently explored.

In the IceMelting 1.0 study, complete blood count analysis revealed that patients who achieved MPR tended to show a reduced derived neutrophil‐to‐lymphocyte ratio (dNLR) after one cycle of neoadjuvant therapy [17]. This decrease was not observed in non‐MPR patients, even after three treatment cycles. After the full three cycles of neoadjuvant therapy, MPR patients also showed lower dNLR than non‐MPR patients. In addition, post‐treatment dNLR was positively correlated with CD66b^+^PD‐L1^+^ cell infiltration in the tumor parenchyma [17]. These observations suggested the presence of a peripheral blood‐TME axis that may contribute to an immunosuppressive microenvironment and could be modulated by VEGFR‐2 signaling. TANs are increasingly recognized as major regulators of tumor progression and immune evasion. Across cancers, TANs can promote immunosuppression, matrix remodeling [18], metastatic dissemination, and therapy resistance [19]. For example, stress‐induced neutrophil extracellular traps remodel the lung microenvironment into a prometastatic niche [20], whereas in gastric cancer, *Fusobacterium nucleatum* drives TANs recruitment and differentiation toward a PD‐L1^high^ protumor phenotype [21]. In head and neck cancer, Fpr^+^ neutrophils have recently been shown to mediate resistance to combined anti‐LAG‐3 and anti‐PD‐1 therapy [19]. In OSCC, neutrophils enhance tumor invasion through an invadopodia‐dependent pathway, and microbiota‐derived kynurenic acid promotes tumor progression by expanding S100a8^high^S100a9^high^ neutrophils, thereby amplifying IL‐1β‐driven immunosuppression and CD8^+^ T‐cell exhaustion [22]. Collectively, these findings support a protumor role of TANs in OSCC and head and neck cancers. However, whether tumor‐intrinsic VEGFR‐2 signaling drives neutrophil reprogramming in OSCC remains unclear. Therefore, we investigated the impact of VEGFR‐2 signaling and its downstream pathway on neutrophil phenotype and function in OSCC.

## 2 Results

### 2.1 PD‐L1^++^CD66b^+^ Neutrophil‐Enriched Cells are Present in Human OSCC and are Associated With Aggressive Clinicopathological Features

**FIGURE 1:**
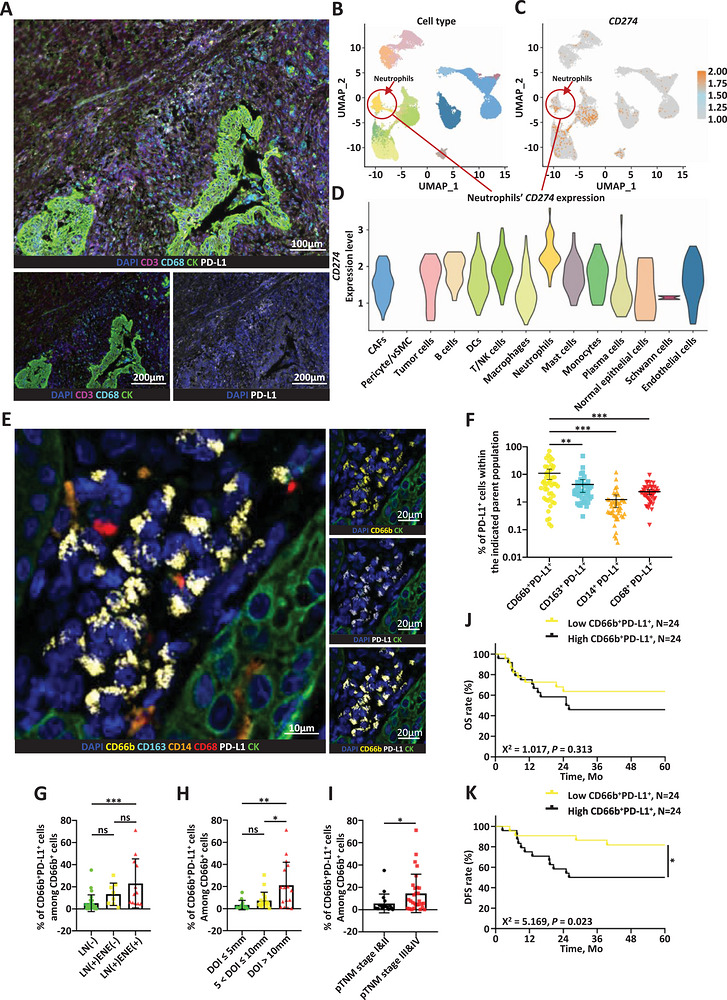
PD‐L1 is expressed on CD66b^+^ cells in OSCC, and high CD66b^+^PD‐L1^+^ cell density is associated with more aggressive clinicopathological features. (A) mIHC showing PD‐L1 staining in cells outside the CPS‐counted compartments; (B) UMAP showing clusters of cells from 6 OSCC patients; (C) UMAP showing *CD274* expression on different clusters of cells from the 6 OSCC patients; (D) Violin plot showing *CD274* expression on different clusters of cells from the 6 OSCC patients; (E) mIHC staining of representative spot from the tissue microarray (TMA) of 48 OSCC patients, showing PD‐L1 expression in CD66b^+^ cells; (F) Quantitative analysis of PD‐L1 expression within each indicated parent population (CD66b^+^ cells, CD163^+^ cells, CD14^+^ cells, or CD68^+^ cells) by mIHC; (G) Percentage of CD66b^+^PD‐L1^+^ cells in patients without lymph node metastasis (LN‐MT), with LN‐MT but without extranodal extension (ENE), or with LN‐MT and ENE; (H) Percentage of CD66b^+^PD‐L1^+^ cells of patients with different depth of invasion (DOI); (I) Percentage of CD66b^+^PD‐L1^+^ cells of patients with different pathological TNM (pTNM) stage; (J) Kaplan‐Meier analysis of overall survival (OS) based on the CD66b^+^PD‐L1^+^ cells level in 48 OSCC patients; (K) Kaplan‐Meier analysis of disease‐free survival (DFS) based on the CD66b^+^PD‐L1^+^ cells level in 48 OSCC patients. ns: non‐significant; ^*^ *p* < 0.05; ^**^ *p* < 0.01; ^***^ *p* < 0.001; mIHC: multiplex‐immunohistochemical; TMA: tissue micro‐array; LN‐MT: lymph node metastasis; ENE: extra‐node extension; pTNM: pathological TNM; OS: overall survival; DFS: disease‐free survival.

A population of PD‐L1‐positive cells that should not be included in CPS calculation was observed in baseline biopsy specimens from the IceMelting 1.0 trial (NCT04393506) (Figure S1A). To further characterize these cells, we performed mIHC staining for PD‐L1 together with markers of CPS‐counted cells, including DAPI for nuclei, pan‐CK for tumor cells, CD68 for macrophages, and CD3 for lymphocytes (Figure 1A). This analysis showed that PD‐L1 was also expressed by a population of cells outside the conventional CPS‐counted compartments. Serial hematoxylin‐eosin‐stained sections were then used to assist morphological identification (Figure S1B). Based on their neutral pink cytoplasmic staining and lobulated nuclei, these cells were suspected to be neutrophils. Consistently, analysis of our in‐house scRNA‐seq dataset from pretreatment biopsy specimens of six LAROSCC patients showed that *CD274* expression was enriched in neutrophil clusters (Figure 1B–D and Figure S1C). We also referred to the Human Protein Atlas [23] and found that neutrophils showed the highest *CD274* expression among immune cell populations (Figure S1D). Because neutrophils are among the earliest immune cells recruited to tumor or inflammatory sites and are subsequently cleared by the mononuclear phagocyte system (MPS), we generated a 7‐color mIHC panel on the Vectra 3.0 platform to verify neutrophil identity and document PD‐L1 distribution across immune cell subsets in a human OSCC tissue microarray (TMA) containing 48 paired tumor and adjacent tissues (Figure 1E and Figure S2A). The panel included DAPI for nuclei, pan‐CK for tumor cells, CD68 for macrophages, CD163 for M2‐polarized macrophages, CD14 for monocytes, CD66b for neutrophils, and PD‐L1. CD68 and CD163 were used to cover a broad macrophage compartment, although these two markers alone cannot fully distinguish M1 and M2 states. Approximately 11.0% of CD66b^+^ cells were PD‐L1‐positive (Figure 1F), a proportion higher than that observed in CD14^+^ cells (1.2%), CD68^+^ cells (2.4%), or CD163^+^ cells (4.4%). A few CD14^+^CD66b^+^PD‐L1^+^ and CD68^+^CD66b^+^PD‐L1^+^ triple‐positive cells were also observed (Figure S2A, white arrows), which may represent MPS cells that had phagocytosed PD‐L1^+^ neutrophils.

Based on the elevated PD‐L1 expression in CD66b^+^ neutrophils compared with other myeloid subsets, we further evaluated the clinical significance of the CD66b^+^PD‐L1^+^ population within the TME. High CD66b^+^PD‐L1^+^ cell density was associated with aggressive clinicopathological features. Tumors with elevated CD66b^+^PD‐L1^+^ infiltration showed a higher frequency of lymph node metastasis with extranodal extension (ENE) (Figure 1G, *p* < 0.001). CD66b^+^PD‐L1^+^ cell density also positively correlated with depth of invasion (DOI) (Figure 1H). Tumors with DOI >10 mm, which are classified as at least T3 disease, showed significantly higher CD66b^+^PD‐L1^+^ cell density than tumors with DOI of 5–10 mm (Figure 1H, *p* = 0.012) or DOI <5 mm (Figure 1H, *p* = 0.003). Consistently, patients with higher CD66b^+^PD‐L1^+^ cell density were more likely to present with advanced pathological TNM stage (III‐IV) than those with lower density (Figure 1I, 15.3% vs. 5.5%, *p* = 0.028). Although overall survival (OS) did not differ significantly between the high‐ and low‐density groups (Figure 1J), patients with high CD66b^+^PD‐L1^+^ cell density had significantly shorter disease‐free survival (DFS) by log‐rank test (Figure 1K, *p* = 0.023). In contrast, stratification by other PD‐L1^+^ myeloid populations (CD14^+^, CD68^+^, and CD163^+^) did not reveal significant differences in OS or DFS (Figure S2B–G). Together, these findings indicate that the CD66b^+^PD‐L1^+^ neutrophil‐enriched population is present in the OSCC TME and is associated with enhanced metastatic propensity and shorter DFS.

To further validate these findings, we included an independent cohort of 57 OSCC patients (Table S2). This cohort was stratified into high‐ and low‐density groups according to the percentage of CD66b^+^PD‐L1^+^ cells among CD66b^+^ cells by mIHC (Figure S3A). Consistent with the original cohort, patients with lymph node metastasis and ENE had higher CD66b^+^PD‐L1^+^ cell abundance than patients with lymph node metastasis but no ENE (Figure S3B, *p* < 0.001) or patients without lymph node metastasis (Figure S3B, *p* < 0.001). Patients with DOI >10 mm also exhibited higher CD66b^+^PD‐L1^+^ cell abundance than those with DOI of 5–10 mm (Figure S3C, *p* = 0.035) or DOI <5 mm (Figure S3C, *p* = 0.014). Patients with higher CD66b^+^PD‐L1^+^ cell density were more likely to present with advanced pathological TNM stage (III‐IV) than those with lower density (Figure S3D, 16.4% vs. 1.7%, *p* = 0.009). Similar to the original cohort, OS did not differ significantly between the low‐ and high‐density groups (Figure S3E, *p* = 0.414), whereas the low‐density group showed a higher DFS rate than the high‐density group (Figure S3F, *p* = 0.020).

### 2.2 OSCC VEGFR‐2 Expression is Positively Correlated With Infiltration of PD‐L1^+^ Neutrophils

We previously demonstrated that neoadjuvant VEGFR2i combined with anti‐PD‐1 achieved a high MPR rate in patients with LAROSCC [5]. Although CD31 and α‐SMA fluorescence intensity was reduced after treatment, indicating a strong antiangiogenic effect across the cohort, this reduction did not differ between patients who achieved MPR and those who did not. We therefore hypothesized that differences in pathological response may be attributable, at least in part, to distinct immunomodulatory effects of VEGFR2i. Based on this rationale, we examined whether VEGFR‐2 and its downstream pathway were associated with PD‐L1^+^ neutrophil infiltration in OSCC. In the TCGA‐HNSC cohort, *KDR* expression, which encodes VEGFR‐2, was higher in tumors than in paired normal tissues (Figure S4A) and positively correlated with neutrophil infiltration (Figure S4B).

**FIGURE 2:**
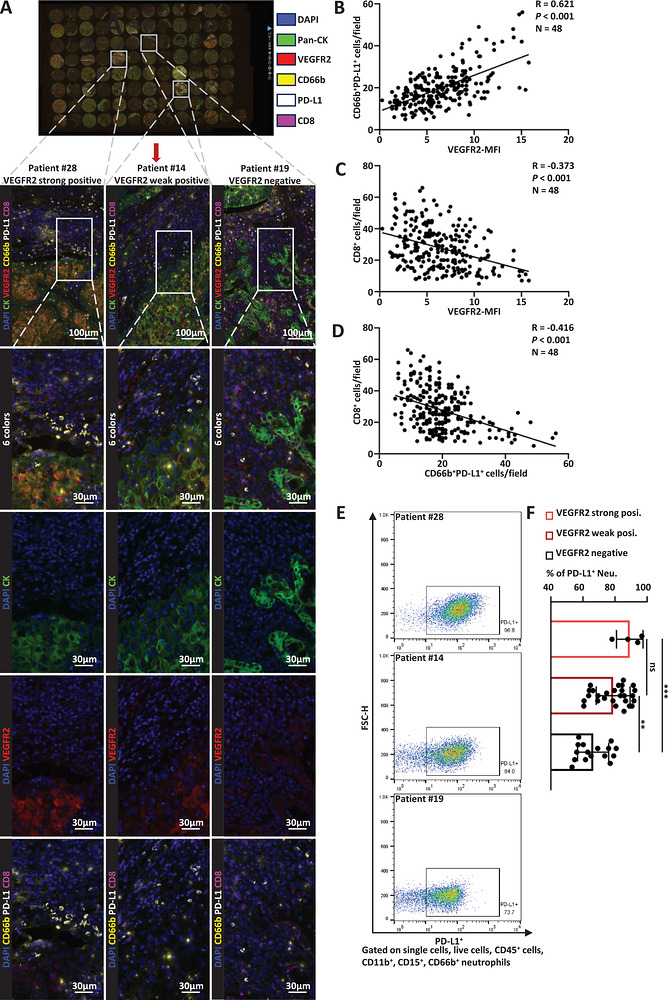
VEGFR‐2 expression is associated with increased CD66b^+^PD‐L1^+^ cell infiltration and decreased CD8^+^ T‐cell infiltration. (A) mIHC staining of the cohort showing VEGFR‐2 expression and CD66b^+^PD‐L1^+^ cells and CD8^+^ cells; (B) CD66b^+^PD‐L1^+^ cell infiltration is positively correlated with VEGFR‐2 expression; five random fields in each TMA spot were used for quantitative analysis; (C) VEGFR‐2 expression is negatively correlated with CD8^+^ T‐cell infiltration; five random fields in each TMA spot were used for quantitative analysis; (D) CD66b^+^PD‐L1^+^ cell infiltration is negatively correlated with CD8^+^ T‐cell infiltration; five random fields in each TMA spot were used for quantitative analysis; (E) Flow cytometry showing PD‐L1^+^ neutrophils in patients with different VEGFR‐2 expression levels; (F) Quantitative analysis of the flow cytometry; mIHC: multiplex‐immunohistochemical; MFI: mean fluorescence intensity; Posi.: positive.

We next stained the same TMA cohort containing 48 paired OSCC and adjacent tissues with a 6‐color mIHC panel to evaluate the relationship between tumor VEGFR‐2 expression and neutrophil PD‐L1 (Figure 2A). VEGFR‐2 expression in OSCC tissues positively correlated with CD66b^+^PD‐L1^+^ cell infiltration (Figure 2B) and negatively correlated with CD8^+^ T‐cell infiltration (Figure 2C). CD66b^+^PD‐L1^+^ cell infiltration also negatively correlated with CD8^+^ T‐cell infiltration (Figure 2D). Flow cytometric analysis of tissues from the TMA cohort further showed that tumors with higher VEGFR‐2 expression contained a greater abundance of PD‐L1^+^ neutrophils (Figure 2E,F and Figure S5D).

### 2.3 OSCC VEGFR‐2 can Induce PD‐L1 Expression in Neutrophils in Vitro and in Vivo

**FIGURE 3:**
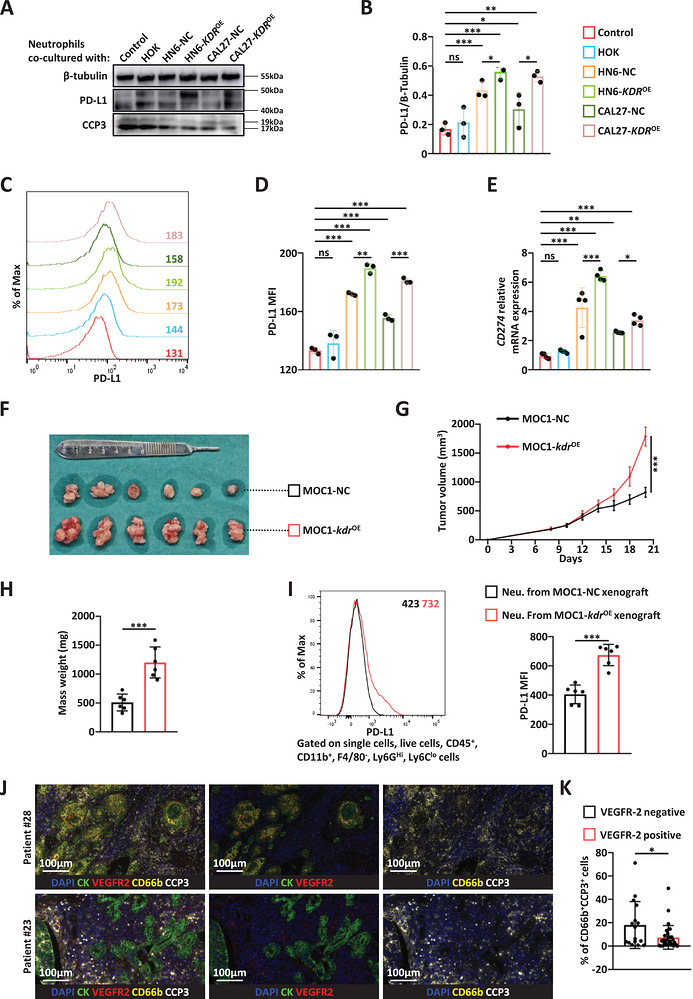
OSCC VEGFR‐2 expression positively correlates with neutrophil PD‐L1 expression and negatively correlates with neutrophil apoptosis. (A) Western blotting detection of PD‐L1 and cleaved caspase‐3 expression in neutrophils after 6 h of coculture with different cell lines; (B) Quantitative analysis of the western blot; (C) Flow cytometry detection of PD‐L1 expression on neutrophils after 6 h of coculture with different cell lines; (D) Quantitative analysis of the flow cytometry; (E) RT‐qPCR detection of relative *CD274* mRNA expression after 6 h of coculture with different cell lines; (F) Representative images of MOC1‐NC and MOC1‐*kdr* ^OE^ xenografts; (G) Tumor growth of MOC1‐NC and MOC1‐*kdr* ^OE^ xenograft; (H) Tumor weight of MOC1‐NC and MOC1‐*kdr* ^OE^ xenografts; (I) Flow cytometry detection of PD‐L1 expression on neutrophils from xenografts formed by MOC1‐NC or MOC1‐*kdr* ^OE^; (J) mIHC staining of the cohort showing VEGFR‐2 expression and CD66b^+^CCP3^+^ cells. (K) Quantitative analysis of patients with negative or positive VEGFR‐2 expression and CD66b^+^CCP3^+^ cells; CCP3: cleaved caspase 3; MFI: mean fluorescence intensity; Neu.: Neutrophil; ns: non‐significant; ^*^ *p* < 0.05; ^**^ *p* < 0.01; ^***^ *p* < 0.001.

To further investigate whether OSCC VEGFR‐2 signaling can induce PD‐L1 expression in neutrophils, human peripheral blood neutrophils were isolated by Percoll separation followed by CD66b^+^ magnetic selection (Figure S5A,D). Neutrophils were then co‐cultured with OSCC‐*KDR* ^OE^ cells or ‐NC cells in a 0.4 µm pore‐size Transwell system, which prevented direct cell‐cell contact while allowing soluble factors to diffuse between chambers. Neutrophils were seeded in the upper chamber, and OSCC cell lines were cultured in the lower chamber (Figure S5A). Among five commonly used OSCC cell lines (SCC9, HN6, CAL27, SCC25, and HN30), HN6 and CAL27 showed the lowest baseline VEGFR‐2 expression (Figure S5B, left) and were therefore selected for construction of human OSCC VEGFR‐2‐overexpressing and control cell lines (‐*KDR* ^OE^ and ‐NC) by lentiviral transfection followed by antibiotic selection (Figure S5B, right). Mouse OSCC VEGFR‐2‐overexpressing and control cell lines (‐*kdr* ^OE^ and ‐NC) were similarly constructed in the C57BL/6‐derived MOC1 cell line (Figure S5C). After 6 h of co‐culture, PD‐L1 protein expression was assessed by western blotting (Figure 3A,B) and flow cytometry (Figure 3C,D), and *CD274* mRNA expression was assessed by RT‐qPCR (Figure 3E). OSCC‐*KDR* ^OE^ cells induced the strongest upregulation of PD‐L1/*CD274* in neutrophils. In the subcutaneous xenograft model, MOC1‐*kdr* ^OE^ cells markedly accelerated tumor growth (Figure 3F–H). Flow cytometry showed that *kdr* ^OE^ tumors contained more PD‐L1^+^ neutrophils than NC tumors (Figure 3I; Figure S5E). In TMA‐mIHC analysis, VEGFR‐2‐positive tumors contained fewer CD66b^+^CCP3^+^ cells than VEGFR‐2‐negative tumors (Figure 3J,K). Consistently, neutrophils co‐cultured with OSCC‐*KDR* ^OE^ cells showed reduced CCP3 expression in vitro (Figure 3A), suggesting that high VEGFR‐2 expression may suppress neutrophil apoptosis. Together, these results indicate that OSCC VEGFR‐2 expression can induce PD‐L1 expression in neutrophils in vitro and in vivo.

### 2.4 GM‐CSF is Up‐Regulated in *KDR* ^OE^ OSCC

**FIGURE 4:**
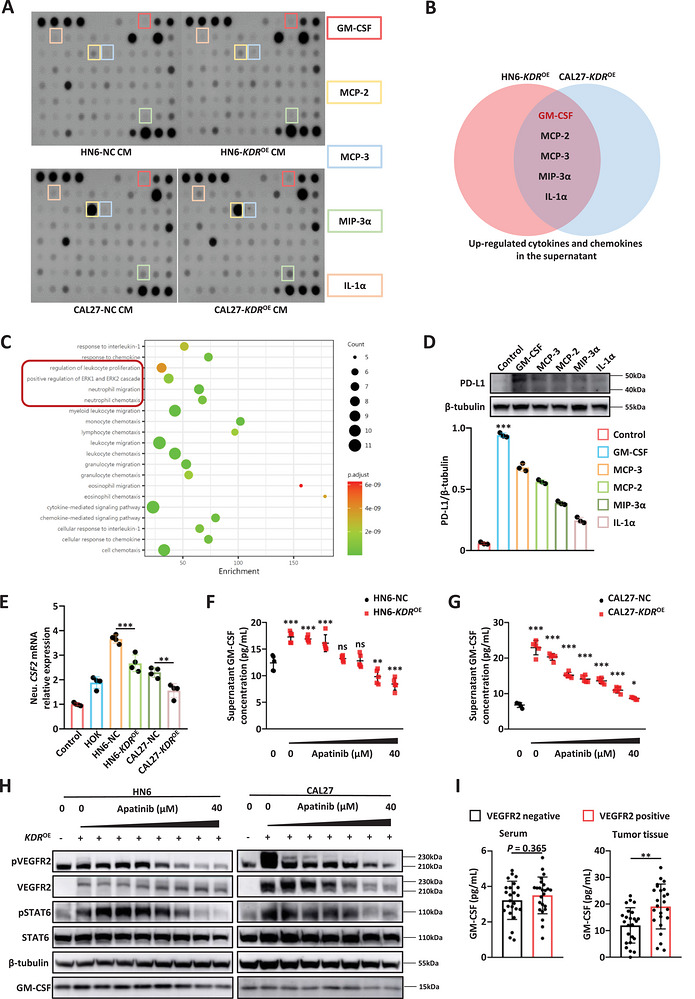
OSCC‐derived GM‐CSF is associated with neutrophil PD‐L1 expression. (A) Cytokine array dot blot showing five upregulated cytokines/chemokines in the supernatants of HN6‐*KDR* ^OE^ and CAL27‐*KDR* ^OE^ cells compared with controls; (B) Venn diagram showing the five co‐upregulated cytokines/chemokines in the supernatants of HN6‐*KDR* ^OE^ and CAL27‐*KDR* ^OE^ cells compared with controls; (C) GO analysis of upregulated cytokines/chemokines in the supernatants of HN6‐*KDR* ^OE^ and CAL27‐*KDR* ^OE^ cells compared with controls; (D) Western blotting detection of neutrophil PD‐L1 expression after culture with different cytokines/chemokines for 6 h (upper) and corresponding densitometric analysis (lower); (E) Relative *CSF2* mRNA expression in neutrophils after 6 h of coculture with different cell lines; (F) ELISA detection of GM‐CSF in HN6‐NC or HN6‐*KDR* ^OE^, treated with different concentrations of VEGFR‐2 inhibitor, apatinib. All statistical values were compared with the control group; (G) ELISA detection of GM‐CSF in HN6‐NC or CAL27‐*KDR* ^OE^, treated with different concentrations of VEGFR‐2 inhibitor, apatinib. All statistical values were compared with the control group; (H) Western blotting detection of VEGFR‐2, STAT6, and GM‐CSF in HN6‐NC or HN6‐*KDR* ^OE^, and CAL27‐NC or CAL27‐*KDR* ^OE^, treated with different concentrations of VEGFR‐2 inhibitor, apatinib; (I) ELISA detection of GM‐CSF expression in serum or tumor tissue of the cohort. Neu.: neutrophils; ns: non‐significant; ^*^ *p* < 0.05; ^**^ *p* < 0.01; ^***^ *p* < 0.001.

To investigate why OSCC‐*KDR* ^OE^ cells induce neutrophil PD‐L1 expression, we reasoned that soluble paracrine factors were likely involved because tumor cells and neutrophils were separated by a non‐contact transwell system. Conditioned medium (CM) from OSCC‐*KDR* ^OE^ and OSCC‐NC cell lines was therefore analyzed using a cytokine microarray (Figure 4A). Five cytokines/chemokines were consistently upregulated in both HN6‐*KDR* ^OE^ and CAL27‐*KDR* ^OE^ cells: GM‐CSF, MCP‐2, MCP‐3, MIP‐3α, and IL‐1α (Figure 4B). These upregulated cytokines/chemokines were enriched in pathways related to leukocyte proliferation, ERK signaling, neutrophil migration, and chemotaxis (Figure 4C). Each cytokine/chemokine was then added to ex vivo neutrophils. A concentration gradient was tested for each of the five factors, and the concentration that produced the highest PD‐L1 expression by western blotting was selected as the optimal working concentration (Figure S6A–E). These concentrations (GM‐CSF, 50 ng/mL; MCP‐3, 10 ng/mL; MCP‐2, 10 ng/mL; MIP‐3α, 100 ng/mL; IL‐1α, 20 ng/mL) were then used for comparison. Because the cytokine dose‐gradient blots were exposed separately (Figure S6A–E) and were intended to determine the optimal working concentration for each individual factor, they were not used for direct cross‐cytokine comparison. Instead, the relative PD‐L1‐inducing effects of the five factors were compared under the same experimental setting (Figure 4D), where GM‐CSF showed the strongest PD‐L1‐inducing effect among the co‐upregulated cytokines/chemokines.

To exclude the possibility that neutrophil‐derived autocrine GM‐CSF accounted for the observed effect, we assessed *CSF2* mRNA expression in neutrophils after co‐culture. Neutrophils cultured with OSCC‐*KDR* ^OE^ cells showed lower *CSF2* mRNA levels than those cultured with OSCC‐NC cells by RT‐qPCR (Figure 4E), indicating that the extra GM‐CSF was unlikely to originate from neutrophils. In contrast, ELISA and western blotting showed that OSCC‐*KDR* ^OE^ cell lines secreted more GM‐CSF than OSCC‐NC cell lines, and VEGFR‐2 inhibition reduced GM‐CSF secretion in a concentration‐dependent manner (Figure 4F–H). We also examined serum and tumor tissues from the TMA cohort and found that patients with VEGFR‐2‐positive tumors had higher GM‐CSF levels in tumor tissues, but not in serum (Figure 4I). These results indicate that GM‐CSF is upregulated in OSCC‐*KDR* ^OE^ cell lines and in VEGFR‐2^high^ OSCC TME.

### 2.5 VEGFR‐2/STAT6/*CSF*2 Signaling Axis Promotes GM‐CSF Synthesis and Secretion

**FIGURE 5:**
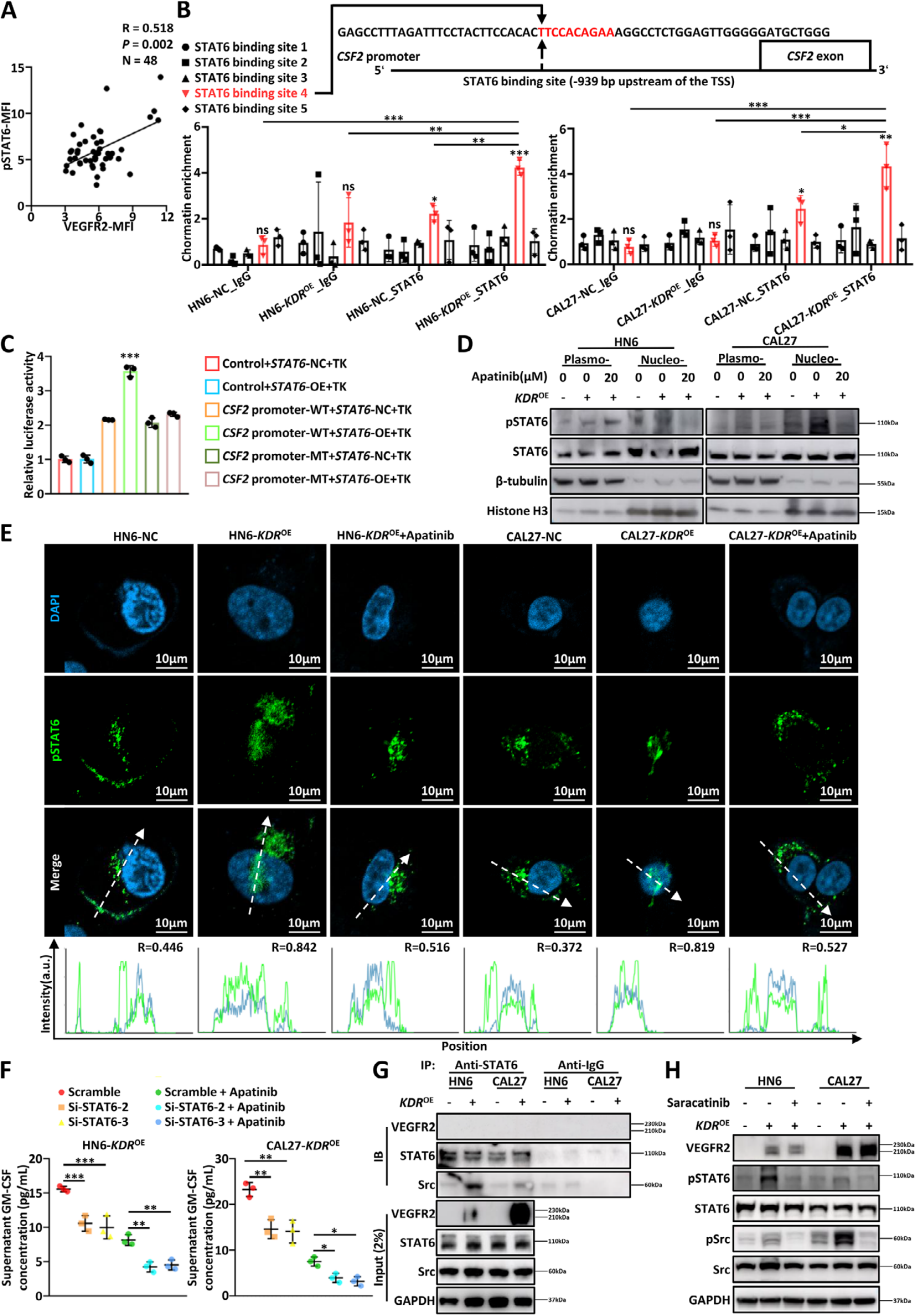
VEGFR‐2 phosphorylation promotes Src‐dependent STAT6 nuclear translocation and CSF2 transcription. (A) Quantitative analysis of mIHC staining, the relationship between pSTAT6 and VEGFR‐2 expression; (B) ChIP‐qPCR showing STAT6 binding site on *CSF2* promoter; (C) Dual luciferase assay showing *CSF2* transcription activation after STAT6 binding; (D) After the indicated treatment, cytoplasm protein and nucleus protein from HN6‐*KDR* ^OE^ and CAL27‐*KDR* ^OE^ were separated and western blot analysis were used to assess the location of pSTAT6; (E) Representative IF staining of pSTAT6 (green) and DAPI staining (blue) in human OSCC cell lines, HN6 and CAL27, ‐NC and ‐*KDR* ^OE^ groups were pretreated with apatinib treated for 12 h, as shown by confocal microscopy images; the colocalization of pSTAT6 (green) and DAPI (blue) was estimated with plot profile using ImageJ. Correlation coefficient *R* > 0.6 was considered well‐collocated, while *R* < 0.6 was taken as poorly collocated; (F) ELISA was used to assess the knockdown of STAT6 for the rescue experiment; (G) Src binds to STAT6 upon VEGFR‐2 overexpression but not VEGFR‐2 itself; (H) Western blot showing STAT6 phosphorylation and its relationship with Src phosphorylation upon VEGFR‐2 overexpression; ns: non‐significant; ^*^ *p* < 0.05; ^**^ *p* < 0.01; ^***^ *p* < 0.001; MFI: mean fluorescence intensity.

We next investigated how GM‐CSF was upregulated in OSCC‐*KDR* ^OE^ cells. Using the hTFtarget and JASPAR databases, STAT6 was predicted as the most likely transcription factor to bind the *CSF2* promoter (Figure S7A). TMA‐mIHC staining for DAPI, pan‐CK, pSTAT6, and VEGFR‐2 revealed a positive correlation between pSTAT6 and VEGFR‐2 expression (Figure 5A and Figure S7B). Other STAT family transcription factors predicted with lower probability, including STAT1, STAT3, and STAT5, were also assessed by western blotting (Figure S7C). Among these factors, only STAT6 phosphorylation was consistently increased after *KDR* ^OE^ in both HN6 and CAL27 cells and consistently inhibited by VEGFR2i treatment (Figure 4H and Figure S7C). Apatinib also inhibited VEGFR‐2 phosphorylation and STAT6 phosphorylation in a concentration‐dependent manner (Figure 4H).

To further examine the relationship between STAT6 and the CSF2 promoter, we performed STAT6 ChIP (Figure S7D). ChIP‐qPCR showed that STAT6 bound the *CSF2* promoter region at approximately ‐939 bp upstream of the transcription start site (TSS), and this binding was enhanced in both OSCC‐*KDR* ^OE^ cell lines (Figure 5B). We then performed a dual‐luciferase reporter assay to determine whether STAT6 binding to the *CSF2* promoter directly regulates *CSF2* transcription. Luciferase reporter constructs containing the full‐length *CSF2* promoter (*CSF2* promoter‐WT, −1965 bp–+35 bp) or a mutant promoter (*CSF2* promoter‐MT, −942 bp to −927 bp upstream of the TSS; CACTTCCACAGAAAGG mutated to ACAGGAACACTCCCTT) were cotransfected with a STAT6 expression plasmid. STAT6 overexpression markedly increased the luciferase activity of the *CSF2* promoter‐WT construct, but not that of the *CSF2* promoter‐MT construct (Figure 5C).

Nuclear‐cytoplasmic fractionation showed that phosphorylated STAT6 was mainly enriched in the nucleus after *KDR* ^OE^ in both OSCC cell lines, and this nuclear accumulation was inhibited by VEGFR2i (Figure 5D). Confocal immunofluorescence microscopy further confirmed increased nuclear localization of pSTAT6 in OSCC‐*KDR* ^OE^ cells compared with control cells, as indicated by a higher colocalization coefficient between pSTAT6 (green) and DAPI (blue) (Figure 5E, rows 2 and 5). This effect was significantly inhibited by VEGFR2i in OSCC‐*KDR* ^OE^ cells (Figure 5E, rows 3 and 6). These findings indicate that ‐*KDR* ^OE^ enhances STAT6 phosphorylation and nuclear translocation in OSCC cells. In rescue experiments, *STAT6* knockdown by siRNA (Figure S7E) significantly reduced GM‐CSF levels in the supernatant of ‐*KDR* ^OE^ cell lines (Figure 5F).

Given that ‐*KDR* ^OE^ markedly increased STAT6 phosphorylation, nuclear translocation, and downstream transcriptional activity in both OSCC cell lines, we next examined whether VEGFR‐2 directly interacts with STAT6. Co‐immunoprecipitation (Co‐IP) assays in HN6 and CAL27 cells showed successful precipitation of STAT6, but VEGFR‐2 was not detected in the STAT6 immunocomplexes (Figure 5G, first blot), suggesting that VEGFR‐2 does not directly bind STAT6. We then explored whether ‐*KDR* ^OE^ activates STAT6 through the classical upstream pathway. Western blot analysis showed that the canonical activating phosphorylation sites of JAK1, JAK2, and JAK3 were not increased after VEGFR‐2 overexpression (Figure S7F). Consistently, RT‐qPCR showed that VEGFR‐2 overexpression did not increase *IL‐4* or *IL‐13* mRNA levels (Figure S7G), arguing against involvement of the classical IL‐4/IL‐13‐JAK‐STAT6 pathway. Previous studies have shown that VEGF signaling can induce STAT6 phosphorylation and nuclear translocation through VEGFR‐2 and have further suggested that Src kinases may participate in VEGF‐induced STAT signaling [24]. In addition, Src is a well‐established downstream effector of VEGFR‐2 signaling [25]. Based on this rationale, we focused on Src as a candidate noncanonical intermediary linking VEGFR‐2 to STAT6 activation. Western blotting showed that Src phosphorylation was consistently increased in both HN6‐*KDR* ^OE^ and CAL27‐*KDR* ^OE^ cells, and pharmacological inhibition of Src with saracatinib markedly attenuated STAT6 phosphorylation in both cell lines (Figure 5H). Moreover, Co‐IP assays showed that ‐*KDR* ^OE^ enhanced the association between Src and STAT6 in HN6‐*KDR* ^OE^ and CAL27‐*KDR* ^OE^ cells (Figure 5G, third blot). Together, these data support a model in which VEGFR‐2 promotes STAT6 phosphorylation through a Src‐dependent noncanonical pathway.

In summary, ‐*KDR* ^OE^ or VEGFR‐2^high^ status in OSCC induces Src‐dependent STAT6 phosphorylation and nuclear translocation, thereby promoting *CSF2* transcription and GM‐CSF synthesis.

### 2.6 GM‐CSF Promotes Neutrophil PD‐L1 Expression Through STAT5 and mTOR/S6K Pathway

**FIGURE 6:**
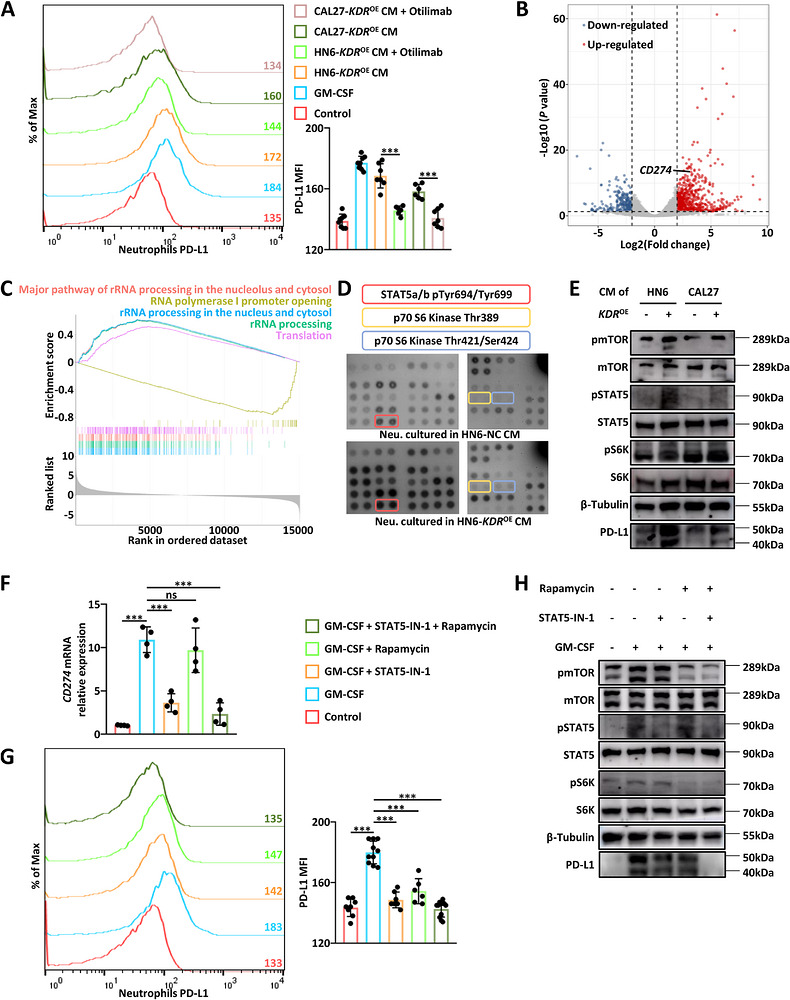
GM‐CSF induces neutrophil PD‐L1 expression through STAT5 and mTOR/S6K signaling. (A) Flow cytometry detection of PD‐L1 expression on human peripheral blood neutrophils cultured in different circumstances; (B) Volcano plot showing upregulated and downregulated genes in human peripheral blood neutrophils after GM‐CSF treatment; (C) The five most up‐regulated pathways in human peripheral blood neutrophils after GM‐CSF treatment; (D) Cytokine array dot blot showing upregulated kinases in neutrophils treated with CM from HN6‐NC or HN6‐*KDR* ^OE^ cells; (E) Western blot detection of mTOR/S6K, STAT5, and PD‐L1 expression in human peripheral blood neutrophils treated with CM from HN6‐*KDR* ^OE^, CAL27‐*KDR* ^OE^, or their control cells; (F) RT‐QPCR detection of relative *CD274* mRNA expression in human peripheral blood neutrophils after treatment with GM‐CSF, STAT5‐IN‐1, or rapamycin. GM‐CSF, 50 ng/mL; STAT5‐IN‐1, 10 µM; rapamycin, 100 nM; (G) Flow cytometry detection of PD‐L1 expression on human peripheral blood neutrophils after treated with GM‐CSF, STAT5 inhibitor, STAT5‐IN‐1 or mTOR inhibitor, rapamycin; (H) Western blot detection of mTOR/S6K, STAT5, and PD‐L1 expression in neutrophils treated with GM‐CSF, STAT5‐IN‐1, or rapamycin; CM: conditioned medium; MFI: mean fluorescence intensity; ns: non‐significant; ^*^ *p* < 0.05; ^**^ *p* < 0.01; ^***^ *p* < 0.001.

Because neutrophils were co‐cultured with tumor cells in a 0.4 µm pore‐size Transwell system, we inferred that neutrophil PD‐L1 expression was induced through soluble factors rather than direct cell‐cell contact. To simplify the model, CM from OSCC‐*KDR* ^OE^ cells was applied to human peripheral blood neutrophils ex vivo. Otilimab was used to neutralize GM‐CSF in CM. After 6 h of stimulation, flow cytometry showed that both recombinant human GM‐CSF and CM from OSCC‐*KDR* ^OE^ cells increased neutrophil PD‐L1 expression, whereas GM‐CSF neutralization reversed CM‐induced PD‐L1 upregulation (Figure 6A). RNA‐seq was then performed on neutrophils stimulated with GM‐CSF for 6 h to explore differentially expressed genes. As shown in the volcano plot, *CD274* mRNA was elevated after GM‐CSF stimulation (Figure 6B). Gene enrichment analysis showed that the most upregulated pathways were related to protein synthesis (Figure 6C). To further explore downstream signaling, we used a human phospho‐kinase array to profile pathway activation in neutrophils treated with CM from HN6‐NC or HN6‐*KDR* ^OE^ cells. STAT5a/b pTyr694/Tyr699 was the most strongly upregulated phospho‐kinase signal, and integration with RNA‐seq results further identified p70 S6 kinase Thr389 and Thr421/Ser424 as candidate mediators of PD‐L1 upregulation in neutrophils (Figure 6D). These phosphorylation events were validated by western blotting (Figure 6E). To determine whether these pathways mediated PD‐L1 induction, neutrophils were treated with the STAT5 inhibitor STAT5‐IN‐1, the mTOR inhibitor rapamycin, or both. After 6 h of treatment, STAT5 inhibition did not reverse GM‐CSF‐induced *CD274* mRNA upregulation, whereas mTOR inhibition did (Figure 6F). However, both STAT5 and mTOR inhibition reduced PD‐L1 protein expression by flow cytometry and western blotting (Figure 6G,H). These results indicate that GM‐CSF promotes neutrophil PD‐L1 expression through STAT5 and mTOR/S6K signaling.

### 2.7 PD‐L1^+^ Neutrophils Suppress CD8^+^ T Cells Proliferation and Induce CD8^+^ T Cells Exhaustion

**FIGURE 7:**
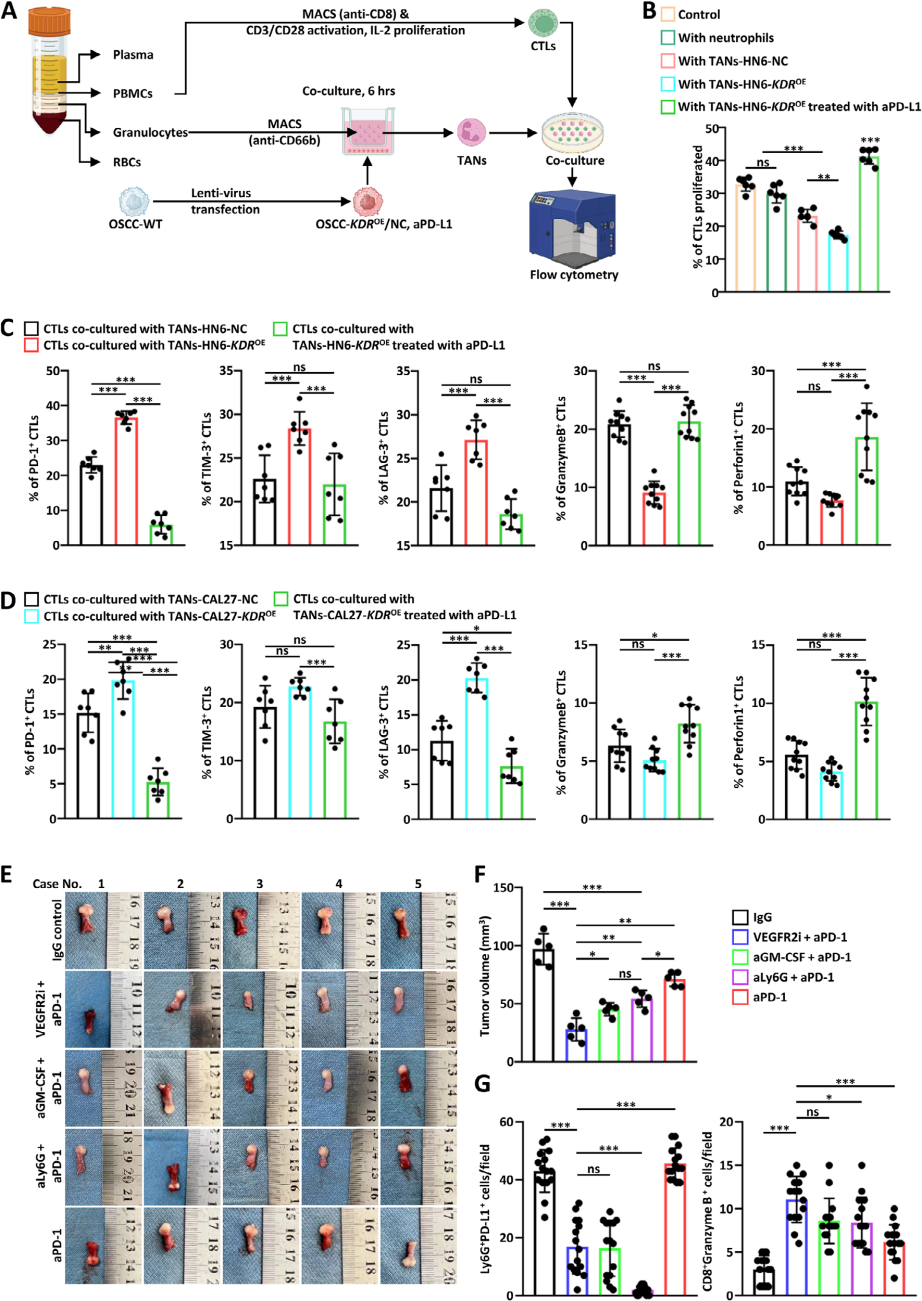
OSCC‐*KDR* ^OE^ ‐derived TANs induce CD8^+^ T‐cell exhaustion. (A) Schematic flowchart; (B) Quantitative flow cytometry analysis showing the proliferation rate of CFSE‐labeled CTLs after co‐culture with neutrophils or TANs; (C) Quantitative flow cytometry analysis showing CTL exhaustion markers after co‐culture with TANs induced by HN6‐NC or HN6‐*KDR* ^OE^ cells; (D) Quantitative flow cytometry analysis showing CTL exhaustion markers after co‐culture with TANs induced by CAL27‐NC or CAL27‐*KDR* ^OE^ cells; (E) Tumor growth of MOC1‐*kdr* ^OE^ tongue orthotopic xenograft treated with the drug combinations; (F) Tumor weight at day 20; (G) Quantitative analysis of Ly6G^+^PD‐L1^+^ cells and CD8^+^Granzyme B^+^ cells within each treatment by mIHC.

Because the major antitumor effect of the immune system is mediated by cytotoxic CD8^+^ T cells, we next evaluated how PD‐L1^+^ TANs affected CD8^+^ T‐cell function after direct contact. CD8^+^ T cells were isolated from healthy donor peripheral blood by Percoll density‐gradient centrifugation followed by anti‐CD8^+^ magnetic selection. The cells were activated with anti‐CD3/CD28 and expanded with IL‐2 for 2 days before subsequent experiments. Neutrophils were isolated from the same donor by Percoll density‐gradient centrifugation followed by anti‐CD66b^+^ magnetic selection and then cocultured with HN6‐NC, HN6‐*KDR* ^OE^, CAL27‐NC, or CAL27‐*KDR* ^OE^ cells for 6 h to generate TANs‐like cells (Figure 7A), as previously described by Xue et al. [14] CD8^+^ T cells were labeled with CFSE and co‐cultured with normal neutrophils, TANs educated by HN6‐NC or HN6‐*KDR* ^OE^ cells, or HN6‐*KDR* ^OE^‐educated TANs treated with a PD‐L1‐blocking antibody. TANs conditioned by HN6‐*KDR* ^OE^ exerted the strongest inhibitory effect on CD8^+^ T‐cell proliferation (Figure 7B and Figure S8A). Consistently, CD8^+^ T cells co‐cultured with HN6‐*KDR* ^OE^‐ or CAL27‐*KDR* ^OE^‐programmed TANs displayed higher expression of the exhaustion‐associated markers PD‐1, TIM‐3, and LAG‐3 than those co‐cultured with HN6‐NC‐ or CAL27‐NC‐programmed TANs (Figure 7C,D and Figure S8B–D). In parallel, the cytotoxic program of CD8^+^ T cells was impaired, as reflected by reduced granzyme B and perforin 1 expression (Figure 7C,D and Figure S8E,F). Importantly, PD‐L1 blockade in neutrophils substantially reversed these effects, restoring CD8^+^ T‐cell proliferation, attenuating exhaustion‐marker expression, and rescuing cytotoxic effector function. These data indicate that TANs educated by OSCC‐*KDR* ^OE^ cells possess enhanced immunosuppressive activity ex vivo and establish neutrophil PD‐L1 as a functional mediator of CD8^+^ T‐cell suppression in this system.

To further verify whether the VEGFR‐2/STAT6/*CSF2* axis induces T‐cell exhaustion through TANs in vivo, MOC1‐*kdr* ^OE^ cells were injected subcutaneously into the flank or orthotopically into the tongue edge of C57BL/6 mice. Treatments started on day 11. Mice were sacrificed on day 20, and xenograft tumors were harvested, weighed, photographed, dissected, and analyzed by flow cytometry or fixed and embedded in paraffin for HE staining and multiplex immunofluorescence. Peripheral blood, bone marrow, and spleen were also collected to assess neutrophil PD‐L1 expression in these compartments. In the subcutaneous xenograft model, VEGFR2i combined with aPD‐1 produced the strongest tumor suppression (Figure S9A–C). GM‐CSF neutralization combined with aPD‐1 also significantly suppressed tumor growth, whereas peritumoral GM‐CSF injection impaired the therapeutic effect of VEGFR2i plus aPD‐1 (Figure S9A–C). Flow cytometry showed no significant differences in neutrophil PD‐L1 expression among treatment groups in peripheral blood, bone marrow, or spleen (Figure S9D–G). In contrast, TANs from IgG‐ and aPD‐1‐treated xenografts showed higher PD‐L1 expression than neutrophils from peripheral blood, bone marrow, or spleen (Figure S9D–H,M,N). This effect was reversed by VEGFR‐2 inhibition or GM‐CSF neutralization, under which TANs PD‐L1 MFI no longer differed from that of neutrophils in other organs (Figure S9O–Q). Peritumoral GM‐CSF injection partially restored neutrophil PD‐L1 expression (Figure S9D,H). These findings suggest that the TME, particularly GM‐CSF, promotes TANs PD‐L1 expression and distinguishes TANs from neutrophils in peripheral or reservoir compartments. For tumor‐infiltrating CD8^+^ T cells (Figure S9S), PD‐1 expression was significantly lower in the VEGFR2i, VEGFR2i+aPD‐1, and aGM‐CSF+aPD‐1 groups than in the other treatment groups (Figure S9I,J), whereas granzyme B expression was significantly higher in the VEGFR2i+aPD‐1 and aGM‐CSF+aPD‐1 groups (Figure S9K,L). Conversely, peritumoral GM‐CSF injection increased PD‐1 expression and reduced granzyme B expression in CTLs (Figure S9I–L). Together, these results indicate that TME‐derived GM‐CSF induces PD‐L1 expression in TANs, suppresses CD8^+^ T‐cell proliferation, and promotes CD8^+^ T‐cell exhaustion.

In the tongue orthotopic model, which more closely recapitulates the OSCC TME, endpoint tumor volume differed significantly among treatment groups (Figure 7E,F). Compared with the IgG control group, anti‐PD‐1 monotherapy moderately reduced tumor volume, whereas VEGFR2i combined with aPD‐1 produced the strongest tumor‐suppressive effect and resulted in the smallest tumors. Neutrophil depletion or GM‐CSF neutralization also markedly suppressed tumor growth, with tumor volumes lower than those in the anti‐PD‐1 group. These data indicate that VEGFR‐2 inhibition enhances the antitumor efficacy of PD‐1 blockade in vivo. mIHC further revealed marked remodeling of the tumor immune microenvironment across treatment groups (Figure 7G and Figure S10A–E). The VEGFR2i+aPD‐1 group exhibited the lowest abundance of PD‐L1^+^Ly6G^+^ cells and the highest abundance of CD8^+^Granzyme B^+^ cells, consistent with the strongest reversal of neutrophil‐associated immunosuppression and the most robust cytotoxic T‐cell activation. In the neutrophil‐depletion group, Ly6G^+^ cells were nearly absent, whereas the number of CD8^+^Granzyme B^+^ cells was slightly lower than that in the VEGFR2i+aPD‐1 group. In the GM‐CSF‐neutralization group, a subset of Ly6G^+^ cells remained detectable, but PD‐L1 expression on these cells was markedly reduced; CD8^+^Granzyme B^+^ cells were also increased, although still slightly fewer than in the VEGFR2i+aPD‐1 group. By contrast, anti‐PD‐1 monotherapy modestly increased CD8^+^Granzyme B^+^ cells compared with the IgG control group, but the magnitude of this increase remained limited relative to the other treatment groups. Moreover, PD‐L1^+^Ly6G^+^ cell abundance in the anti‐PD‐1 group was comparable to that in the IgG group and significantly higher than that in the other treatment groups. Together, these findings indicate that VEGFR‐2 inhibition enhances the therapeutic efficacy of PD‐1 blockade by reducing PD‐L1^+^ neutrophil accumulation and restoring cytotoxic CD8^+^ T‐cell activity, at least in part through suppression of the GM‐CSF‐neutrophil axis.

## 3 Discussion

In this study, we identified PD‐L1‐expressing neutrophils within the OSCC TME that were associated with aggressive clinicopathological features and shorter DFS. We further demonstrated that OSCC VEGFR‐2 signaling promotes PD‐L1 expression in neutrophils through a STAT6‐dependent *CSF2* transcriptional mechanism that increases tumor‐derived GM‐CSF secretion. Tumor‐derived GM‐CSF then acts on neutrophils in the TME and activates STAT5 and mTOR/S6K signaling, leading to enhanced protein synthesis and PD‐L1 upregulation. Functional assays confirmed that these PD‐L1^+^ neutrophils suppress CD8^+^ T‐cell proliferation and promote exhaustion, thereby contributing to an immunosuppressive microenvironment. These findings provide a mechanistic basis for the ability of VEGFR‐2 inhibition to enhance responses to anti‐PD‐1 therapy in OSCC.

Neutrophils have emerged as key modulators of tumor immunity, with distinct subsets exerting either antitumor or protumor functions depending on context [14, 15, 16]. Ng et al. reported three intratumoral neutrophil states that were epigenetically and transcriptionally distinct from neutrophils in the bone marrow, spleen, and blood. These tumor‐educated neutrophils exhibited dcTRAIL‐R1 upregulation, prolonged lifespan, and proangiogenic features [16]. Wu et al. further reported ten neutrophil states across cancers, spanning inflammation, angiogenesis, and antigen presentation. A leucine‐rich diet reprogrammed neutrophil metabolism and epigenetically activated antigen‐presentation programs, thereby boosting T‐cell neoantigen responses and enhancing anti‐PD‐1 therapy [26]. Although PD‐L1 expression has been extensively studied in tumor cells and myeloid cells such as macrophages and dendritic cells [27, 28], its expression on neutrophils within the OSCC TME remains underexplored. Our identification of PD‐L1^+^ neutrophils in human OSCC is consistent with recent scRNA‐seq studies across tumor types [14, 26] and suggests a broader role for neutrophils in immune checkpoint regulation. By integrating scRNA‐seq, IHC, and flow cytometry in OSCC specimens, our data indicate that VEGFR‐2 signaling and its downstream pathway may drive PD‐L1 upregulation in neutrophils within the OSCC TME. VEGFR2i has been widely used in several cancers [29, 30, 31]. In our previous phase I trial, neoadjuvant aPD‐1 combined with VEGFR2i achieved a 40% MPR rate in LAROSCC [5], but the underlying mechanism remained unclear. Here, our data extend those clinical observations by delineating a link between tumor VEGFR‐2/STAT6/*CSF2* signaling and neutrophil PD‐L1 expression through tumor‐derived GM‐CSF.

GM‐CSF generally stimulates hematopoietic progenitors to produce granulocytes and monocytes and participates in immune regulation. In clinical practice, GM‐CSF has been used to prevent chemotherapy‐associated leukopenia and to treat bone marrow dysfunction or myelodysplastic syndrome. It has also been evaluated in sepsis‐associated immunosuppression to restore monocytic immunocompetence [32]. However, it failed to improve impaired neutrophil phagocytosis in critically ill patients, despite increasing monocytic HLA‐DR expression [33]. In tumor immunity, GM‐CSF was initially considered an antitumor cytokine because dendritic cells generated in vitro from monocytes using GM‐CSF can be activated and loaded with tumor‐associated antigens to stimulate T‐cell responses. However, the resulting cell population is heterogeneous and includes both conventional dendritic cells and macrophages [34]. GM‐CSF‐elicited myeloid cells were subsequently found to have immunosuppressive properties [35, 36], indicating that the role of GM‐CSF in cancer remains context‐dependent. In inflammation, GM‐CSF is produced by Th17 cells in response to IL‐23R engagement [37]. Tumor‐derived GM‐CSF has also been reported in colon carcinogenesis [38], breast cancer [39], and laryngeal squamous cell carcinoma [40]. In the present study, we identified tumor‐derived GM‐CSF as a critical cytokine mediating VEGFR‐2‐driven PD‐L1 expression in TANs from OSCC. Using ChIP‐qPCR and dual‐luciferase assays, we showed that VEGFR‐2 activation promotes STAT6 phosphorylation and nuclear entry, enabling STAT6 to bind the CSF2 promoter and drive GM‐CSF transcription. Although STAT6 is classically activated downstream of IL‐4/IL‐13 signaling and regulates targets involved in M2‐like macrophage polarization [41], Th2 differentiation, and B‐cell class switching [42], our data suggest that OSCC cells can engage STAT6 through a non‐canonical VEGFR‐2/Src route. Subsequent neutrophil exposure to GM‐CSF activated STAT5 and mTOR/S6K signaling, leading to enhanced translation and PD‐L1 upregulation. Although STAT3 has also been reported to regulate neutrophil‐mediated immunosuppression and tumor progression [43], STAT3 phosphorylation was less prominent than STAT5 and mTOR/S6K activation after GM‐CSF stimulation in our system, supporting a dominant role for STAT5 and mTOR/S6K in PD‐L1 upregulation. In addition to PD‐L1, MMP‐9 was also upregulated in neutrophils primed with CM from OSCC‐*KDR* ^OE^ cells (not shown), suggesting that VEGFR‐2‐driven TANs programming may also contribute to OSCC metastasis. Together, these findings highlight the complexity of neutrophil plasticity and identify potential therapeutic targets for modulating neutrophil‐mediated immunosuppression.

Our findings have several clinical implications. First, the density of CD66b^+^PD‐L1^+^ neutrophils may serve as a potential biomarker associated with DFS in OSCC, although validation in larger and prospectively designed cohorts is needed. Increased intratumoral neutrophil infiltration has previously been reported as an independent adverse prognostic factor in esophageal squamous cell carcinoma [44]. However, the direct effects of these cells on tumor progression and their indirect effects through immune regulation require further clarification. Our data provide a rationale for VEGFR2i to enhance immune checkpoint blockade by simultaneously targeting angiogenesis and neutrophil‐mediated immunosuppression. This mechanism may help explain the favorable response observed in our previous phase I trial of neoadjuvant aPD‐1 plus VEGFR2i in LAROSCC [5]. Neutrophil PD‐L1 expression has also been reported to promote lung injury in an experimental mouse model of sepsis [45], consistent with the immunosuppressive role of neutrophil PD‐L1 observed here. Nevertheless, immunosuppression is a multicellular process, and additional immune populations, such as helper T cells and M2‐like macrophages, warrant further investigation.

Several limitations should be acknowledged. Although an independent validation cohort was added, the clinical correlations still require confirmation in larger prospective studies. In addition, although multiple models were used to validate the VEGFR‐2/GM‐CSF/PD‐L1 axis, the translational relevance of mouse models to human OSCC remains partially constrained. Other cytokines or cell types may also contribute to neutrophil modulation in the TME and warrant further investigation. In the ex vivo co‐culture assays, peripheral blood neutrophils were enriched by Percoll density‐gradient separation followed by CD66b‐positive selection, which likely reduced contamination by low‐density PMN‐MDSC‐like cells. Nevertheless, CD66b alone cannot fully distinguish conventional neutrophils from PMN‐MDSC‐like granulocytic populations in human tumor tissues. Future studies using additional markers and functional assays are needed to better define the precise identity and differentiation status of these cells. Moreover, the donor setting used in the ex vivo immune‐cell assays was limited and should be further validated using cells from additional donors. Future work should also explore the functional role of neutrophil‐specific PD‐L1 using conditional knockout models and evaluate the therapeutic potential of targeting GM‐CSF or downstream pathways, such as STAT5/mTOR, in combination with immunotherapy. Single‐cell multi‐omics approaches may further resolve neutrophil heterogeneity and plasticity in OSCC.

## 4 Conclusion

In conclusion, we identified a PD‐L1^+^CD66b^+^ neutrophil‐enriched population in the OSCC TME and delineated a VEGFR‐2/STAT6/*CSF2* signaling axis in OSCC cells that drives PD‐L1 expression in neutrophil‐lineage cells, contributing to CD8^+^ T‐cell dysfunction and adverse clinical outcomes. These findings deepen our understanding of neutrophil biology in cancer and provide mechanistic support for combining antiangiogenic agents with immunotherapy in OSCC treatment.

## 5 Methods

### 5.1 Ethics

Before study initiation, written informed consent was obtained from all human participants. The research protocol was conducted in accordance with the Declaration of Helsinki and was approved by the Ethics Committee of the Ninth People's Hospital, Shanghai Jiao Tong University School of Medicine (Shanghai, China). All animal procedures were performed in accordance with the National Institutes of Health Guide for the Care and Use of Laboratory Animals and were approved by the Institutional Animal Care and Use Committee of the same institution.

### 5.2 Specimens

Clinical specimens, including OSCC tumors and paired adjacent normal tissues, were obtained from the Department of Oral and Maxillofacial‐Head & Neck Oncology at Shanghai Ninth People's Hospital, Shanghai Jiao Tong University School of Medicine. A cohort of 48 paired samples for mIHC was collected between March 2015 and September 2018. Pathological diagnosis and clinical staging were established according to the World Health Organization Classification of Tumors and the American Joint Committee on Cancer (AJCC) tumor‐node‐metastasis (TNM) staging system (eighth edition). The clinicopathological characteristics of the 48‐patient discovery cohort and the 57‐patient validation cohort are summarized in Tables S1 and S2.

### 5.3 TCGA Database Analysis

The RNA‐seq data (in FPKM format) and the corresponding clinical information for pan‐cancer analysis, including the Head and Neck Squamous Cell Carcinoma (TCGA‐HNSC) dataset, were downloaded from the official TCGA data portal. The differential expression of the *KDR* gene between tumor and adjacent normal tissues was investigated across all available TCGA cancer types. The relative infiltration level of neutrophils in the TCGA‐HNSC cohort was quantified using the single‐sample Gene Set Enrichment Analysis (ssGSEA) algorithm through the GSVA R package. The gene set used to represent neutrophils was obtained from the well‐established immune signature collection in the literature. Subsequently, the correlation between *KDR* expression and the calculated neutrophil infiltration score was evaluated using Spearman's rank correlation coefficient. The statistical significance of the correlation was assessed, and the result was presented as a scatter plot.

### 5.4 Single‐Cell RNA Sequencing and Analysis

Tumor specimens were rinsed with ice‐cold PBS, mechanically minced, and enzymatically dissociated in DMEM containing collagenase I/II/IV, DNase I, and 5% FBS at 37°C. Single‐cell suspensions were obtained through sequential filtration (70 and 30 µm), red blood cell lysis, and repeated washing. Cell viability and aggregation were assessed using AO/PI staining on the Countstar Rigel S2 system. High‐quality suspensions were adjusted to 200 cells/µL and processed with the DECODER Single Cell 3’ Kit (Dynamic Biosystems, v1.0) for single‐cell capture, cDNA synthesis, amplification, and library preparation. Indexed libraries were constructed following the manufacturer's protocol and sequenced on an Illumina NovaSeq platform using 150‐bp paired‐end reads. Raw sequencing data were aligned to the GRCh38 reference genome using DynamicEX (v1.0.3). Downstream analysis was performed in Seurat (v4.3.0), including quality filtering based on feature counts, UMI counts, and mitochondrial transcript percentages. Putative doublets were removed with DoubletFinder (v2.0.3), and batch effects were mitigated using Harmony (v0.1.1) [46]. Processed data were normalized, scaled, and subjected to PCA, followed by UMAP embedding and Louvain clustering. Cluster‐specific marker genes were identified using the FindAllMarkers function with the Wilcoxon rank‐sum test. The expression level of *CD274* was then assessed across all annotated cell populations and visualized by UMAP and violin plot. The processed single‐cell RNA‐sequencing data generated in this study have been deposited in the Zenodo database under Digital Object Identifier https://doi.org/10.5281/zenodo.21296909.

### 5.5 Cell Lines and Culture

The human HNSCC cell lines SCC9, SCC25, HN30, HN6, and CAL27, and the immortalized human oral keratinocyte HOK cell line, were used in this study. HN6 and HN30 were generously provided by the University of Maryland Dental School (USA), and SCC9, SCC25, CAL27, and HOK were obtained from the American Type Culture Collection (ATCC, USA). HN30, HN6, and CAL27 cells were maintained in Dulbecco's modified Eagle medium (Basal Media), whereas SCC9 and SCC25 cells were maintained in Dulbecco's modified Eagle medium/F12, supplemented with 10% fetal bovine serum (FBS; Gibco, USA), 100 U/mL penicillin, and 100 µg/mL streptomycin (New Cell & Molecular Biotech, China) at 37°C in a humidified 5% CO_2_ incubator. Human promyelocytic HL‐60 cells were used as a neutrophil‐like model for in vitro experiments. HL‐60 cells were obtained from ATCC and maintained in Iscove's modified Dulbecco's medium (IMDM, Basal Media) supplemented with 20% FBS, 100 U/mL penicillin, and 100 µg/mL streptomycin at 37°C in a humidified 5% CO_2_ incubator. HL‐60 cells were differentiated into neutrophil‐like cells (dHL‐60) by culture in basal medium containing 1.25% dimethyl sulfoxide (DMSO) for at least 4 days. The mouse OSCC cell line MOC1 was cultured in 2:1 IMDM/Ham's F10‐F12 medium (Hyclone, USA) supplemented with 5% FBS, 100 U/mL penicillin, 100 µg/mL streptomycin, 5 mg/mL insulin, 40 ng/mL hydrocortisone, and 5 ng/mL EGF. All cells were routinely screened for mycoplasma contamination using a GMyc‐PCR Mycoplasma Test Kit (Yeasen, China).

For neutrophil coculture with tumor cell lines, 24‐well Transwell chambers (Corning, 0.4 µm pore size) were used. Once 80% confluence was reached, transfected OSCC cell lines or HOK cells were collected and seeded in the lower chamber of 0.4 µm pore‐size inserts (2 × 10^4^ cells/well) in basal medium and cultured overnight. The next day, primary neutrophils (2 × 10^4^ cells/well) were suspended in the upper chamber. Further experiments were performed after 4–8 h of co‐culture. To determine whether neutrophil‐derived PD‐L1 mediated CD8^+^ T‐cell suppression, neutrophils were incubated with an anti‐human PD‐L1 blocking antibody (Cat. #329745, BioLegend) at a final concentration of 10 µg/mL for 30 min at 37°C before co‐culture. After antibody pretreatment, neutrophils were cocultured with CD8^+^ T cells in the continued presence of the antibody for the indicated time.

### 5.6 Primary Neutrophil and CD8^+^ T‐Cell Isolation and Culture

Peripheral blood was obtained from healthy donors and processed for neutrophil and CD8^+^ T‐cell isolation. For neutrophil isolation, fresh peripheral blood was separated using Percoll according to the manufacturer's instructions (Cat. #LZS11131, TBDscience, China). Briefly, diluted blood was carefully layered onto a two‐layer Percoll gradient of different densities. After centrifugation, neutrophils at the interface between the low‐density and high‐density layers were collected, and erythrocytes were lysed using ammonium–chloride–potassium buffer. The collected neutrophils were further purified by magnetic separation using a CD66b^+^ positive selection kit (Cat. #130‐104‐913, Miltenyi Biotec, Germany) according to the manufacturer's instructions. After isolation, primary neutrophils were suspended in RPMI 1640 supplemented with 10% FBS and cultured at 37°C in a humidified 5% CO_2_ incubator. For primary CD8^+^ T‐cell isolation, peripheral blood from the same donor was separated using Percoll, and peripheral blood mononuclear cells (PBMCs) were collected from the top of the low‐density layer. PBMCs were further purified using a CD8^+^ positive selection kit (Cat. #130‐096‐495, Miltenyi Biotec, Germany). CD8^+^ T cells were then cultured and expanded with CD3/CD28/IL‐2 stimulation before subsequent experiments.

### 5.7 RNA‐Seq and Analysis

The isolation of neutrophils from peripheral blood of healthy donors was described above. The isolated cells were then resuspended in TRIzol (T9424, Sigma–Aldrich, USA) to extract total RNA. The RNA‐seq was performed by Cosmos Wisdom Co., Ltd (Hangzhou, China). The Illumina Novaseq 6000 platform was used for sequencing. Illumina TruSeq RNA sample prep Kit was used for library construction. The gene expression level was calculated using FPKM. *p* value < 0.05 and |fold change| > 1.5 were set as the threshold for DEGs. Further Gene Ontology (GO) and Kyoto Encyclopedia of Genes and Genomes (KEGG) enrichment analyses were performed with the DEGs to illustrate the biological functions and pathway differences between the different treatments.

### 5.8 Lentiviral Transduction and Screening for Stable Cell Lines

The human *KDR*, mouse *kdr*, and corresponding control lentiviral vectors were constructed by Genomeditech Biotechnology Co., Ltd. *KDR*‐overexpressing (‐*KDR* ^OE^), kdr‐overexpressing (‐*kdr* ^OE^), and control (‐NC) cells were generated by lentiviral transduction according to the manufacturer's protocol. Stable cells were selected with culture medium containing puromycin (Yeasen, China) at a final concentration of 10 µg/mL for 2 weeks. Sequences used for target overexpression are listed (Table S3).

### 5.9 RNA Extraction and Real‐Time PCR Analysis

For tissue RNA extraction, total RNA was extracted from clinical samples using TRIzol reagent (Takara, Japan) and reverse‐transcribed into cDNA using a PrimeScript RT Reagent Kit (Takara, Japan). For cellular RNA extraction, cells were seeded in 6‐well plates and cultured in their respective basal media. After treatment, RNA was extracted using the RNA‐Quick Purification Kit (EZBioscience, China) according to the manufacturer's protocol. Reverse transcription was performed using Evo M‐MLV RT Master Mix (EZBioscience, China). Real‐time PCR was performed using Hieff qPCR SYBR Green Master Mix (EZBioscience, China) on a StepOnePlus Real‐Time PCR System. The 2^ΔΔCt^ method was used to normalize target gene expression to *ACTB*. Primer sequences are listed in Table S3.

### 5.10 Nuclear and Cytoplasmic Proteins Isolation

Transfected OSCC cell lines (‐*KDR* ^OE^ or ‐NC; HN6 and CAL27) were seeded in 10 cm dishes (5 × 10^6^ cells per dish) and treated with VEGFR2i (apatinib, Cat. #S2221, Selleck, USA) or control for 24 h. Cells were washed twice with ice‐cold PBS. Nuclear and cytoplasmic proteins were isolated using a nuclear and cytoplasmic protein extraction kit (Cat. #P0027, Beyotime, China) according to the manufacturer's instructions. Briefly, cells were collected with a cell scraper, transferred to centrifuge tubes, and centrifuged at 2000 g for 5 min. Lysis buffer was added to resuspend the cells, followed by incubation on ice for 15 min. Cytoplasmic proteins were collected by centrifugation at 12 000 g for 5 min at 4°C. The remaining pellets were washed with lysis buffer and subjected to two additional centrifugation steps at 12 000 g for 5 min at 4°C before nuclear proteins were collected. Western blotting was subsequently performed to detect pSTAT6 and STAT6 in the nuclear and cytoplasmic fractions.

### 5.11 Immunoprecipitation Assays

For immunoprecipitation assays, HN6‐NC, HN6‐*KDR* ^OE^, CAL27‐NC, and CAL27‐*KDR* ^OE^ cells were lysed in SDS‐free immunoprecipitation lysis buffer. Cell lysates were incubated overnight with an anti‐STAT6 antibody and protein A/G magnetic beads (Merck Millipore, MA, USA). Proteins bound to STAT6 were analyzed by western blotting.

### 5.12 Western Blotting Analysis

Transfected OSCC cell lines, neutrophil‐like dHL‐60 cells, and primary peripheral blood neutrophils were seeded in 10 cm culture dishes or 6‐well plates. After the indicated treatments, culture medium was removed, and cells were washed twice with ice‐cold PBS. SEMS lysis buffer (Beyotime, China) was then added. Cell lysates were collected and clarified by centrifugation, and protein concentrations were determined using a BCA assay. Equal amounts of protein were mixed with loading buffer and heated at 100°C for 10 min before SDS‐PAGE. Protein samples (20 µg) were loaded onto SurePAGE protein gels (GenScript, USA) and separated at 150 V for 70 min. Proteins were then transferred to 0.22 µm polyvinylidene fluoride membranes (Millipore, USA). Membranes were blocked with 5% skim milk in TBST (Solarbio, China) at room temperature and incubated with primary antibodies at 4°C overnight. After six washes with TBST, membranes were incubated with the corresponding secondary antibodies for 1 h at room temperature. Signals were visualized using enhanced chemiluminescence reagents (New Cell & Molecular Biotech, China) with an Amersham Imager 600. Antibodies are listed in Table S4. Densitometric analysis of the blots was performed using ImageJ [47]. Full‐length raw and unedited immunoblot images corresponding to the cropped blots shown in the main and supplementary figures are provided in Figure S11.

### 5.13 Extracellular Cytokine Analysis

Transfected OSCC‐*KDR* ^OE^ cell lines (HN6 and CAL27) and their control cell lines were seeded in 10 cm dishes and cultured overnight at 37°C in a humidified 5% CO_2_ incubator. The next day, cells were washed twice with PBS and cultured overnight in serum‐free medium. The resulting CM was centrifuged at 1,200 g for 15 min at 4°C and analyzed using the Human Cytokine Array C5 (Cat. #AAH‐CYT‐5‐2, RayBiotech, USA) according to the manufacturer's protocol. Targets are listed in Table S5.

### 5.14 Intracellular Signaling Pathway Analysis

Primary neutrophils were lysed after 6 h of CM treatment. Cell lysates were analyzed using the Proteome Profiler Human Phospho‐Kinase Array Kit (Cat. #ARY003C, R&D Systems, USA) according to the manufacturer's protocol. Targets are listed in Table S6.

### 5.15 Immunocytochemistry (ICC)

Transfected OSCC cell lines were seeded in 3.5 cm confocal dishes (5 × 10^3^ cells/well). After treatment with VEGFR2i or PBS control, cells were incubated with PDTC or serum‐free medium for 1 h, washed twice with precooled PBS, and fixed with 4% paraformaldehyde (PFA; Biosharp, China) for 15 min. Cells were permeabilized with 0.1% Triton X‐100 (Solarbio, China), blocked with 5% goat serum in PBST, and incubated with anti‐phospho‐STAT6 (Tyr641) antibody (D8S9Y, 1:200, Cat. #56554, CST, USA) at 4°C overnight. After two PBS washes, cells were incubated with secondary antibodies for 1 h in the dark at room temperature. Nuclei were stained with DAPI for 15 min. Images were acquired using a Zeiss LSM900 microscope and processed with ZEN 1.1.0.

### 5.16 Chromatin Immunoprecipitation (ChIP)

Chromatin immunoprecipitation was performed using the SimpleChIP Enzymatic Chromatin IP Kit (Cat. #9003, CST, USA) according to the manufacturer's instructions. Treated OSCC cells were fixed, lysed, and sonicated. Anti‐phospho‐STAT6 (Tyr641) antibody (D8S9Y, 1:100, Cat. #56554, CST, USA) or IgG was used to enrich *CSF2* promoter regions. Bound DNA fragments were analyzed by qPCR. Primer sequences are listed in Table S3. STAT6 binding sites in promoters were predicted using the hTFtarget (https://ngdc.cncb.ac.cn/) and JASPAR (http://jaspar.genereg.net/) databases.

### 5.17 Dual‐Luciferase Reporter Assay

HEK293T cells were used for the dual‐luciferase reporter assay. The full‐length *CSF2* promoter region or mutant promoter was cotransfected with the STAT6 expression construct in the pcDNA3.1(+) vector. Cells were harvested after 24 h of culture. Luciferase activity was measured using a Renilla‐Firefly Luciferase Dual Assay Kit (Thermo Scientific, MA, USA).

### 5.18 Enzyme‐Linked Immunosorbent Assay (ELISA)

Transfected OSCC cell lines (HN6‐*KDR* ^OE^ and CAL27‐*KDR* ^OE^) were seeded in 6‐well plates (1 × 10^6^ cells per plate). After 24 h of treatment with different concentrations of apatinib, the culture medium was replaced with serum‐free DMEM for 24 h before supernatant collection. Supernatants (100 µL) and protein standards were added to antibody‐precoated 96‐well plates for human GM‐CSF detection using ELISA kits (Multi Sciences, China) according to the manufacturer's protocol. Absorbance was measured at 450 and 630 nm using a SpectraMax i3 instrument, and concentrations were calculated from standard curves.

### 5.19 Tissue Processing

Tumor tissues from OSCC patients were preserved at −80°C. After mechanical disruption, tissues were digested for 30 min at 37°C in RPMI 1640 containing 80 U/mL DNase (Cat. #abs47047435, Absin, China) and 300 U/mL collagenase type I (Cat. #abs47048000, Absin, China). Digested tissues were passed through a 70 µm nylon mesh, centrifuged, subjected to red blood cell lysis, washed, filtered through a 40 µm mesh, centrifuged again, and resuspended in PBS containing 2.5% FCS and 2 mM EDTA.

### 5.20 Flow Cytometry

Single‐cell suspensions from tumor tissues or in vitro cocultures of neutrophils and CD8^+^ T cells were collected and washed twice with PBS. Zombie Green Fixable Viability Kit (Cat. #423112, BioLegend, USA) was used to identify live cells, and TruStain FcX PLUS (Cat. #156603, BioLegend, USA) was used for Fc blockade. For surface staining, antibodies were incubated with cells in PBS for 30 min on ice. For intracellular staining, cells were fixed and permeabilized (Cat. #554714, BD Biosciences, USA) for 30 min on ice after surface staining, followed by incubation with intracellular antibodies in PBS for 30 min on ice. All antibodies are listed in Table S7. Samples were analyzed using a BD FACSCanto II (BD Biosciences, USA) or NovoCyte Penteon (Agilent, China), and data were analyzed using FlowJo 10.8.1.

### 5.21 Immunohistochemical (IHC) Staining

Paraffin sections of OSCC clinical samples, adjacent normal tissues, and mouse tumors were heated at 65°C for 2 h, dewaxed by three rounds of immersion in 100% dimethylbenzene, rehydrated through sequential immersion in 100%, 90%, and 70% ethanol, and immersed in pure water. Tris‐EDTA antigen retrieval buffer (Proteintech, China) was used for heat‐induced epitope retrieval in a microwave oven. Endogenous peroxidase activity was blocked with 3% H_2_O_2_ (Absin, China) for 15 min, and 10% goat serum (Biosharp, China) was used for blocking. Anti‐PD‐L1 antibody (E1L3N, 1:400, Cat. #13684, CST, USA) was applied to tissue sections and incubated at 4°C overnight. After five PBS washes, goat anti‐mouse/rabbit poly‐HRP‐conjugated secondary antibody (Proteintech, China) was applied. DAB detection kit (Absin, China) was used for visualization, and nuclei were counterstained with hematoxylin (Sangon Biotech, China). After dehydration through graded ethanol and dimethylbenzene, coverslips were mounted with neutral balsam (Absin, China), and slides were scanned using a Vectra automated quantitative pathology system.

### 5.22 HE Staining

Dewaxing was performed on paraffin sections of OSCC samples as described above for IHC staining. The rehydrated sections were then stained with Harris's hematoxylin solution for 5–8 min to visualize cell nuclei. After rinsing in running tap water to remove excess stain, the sections were differentiated in 1% acid ethanol and blued in 0.2% ammonia water or Scott's tap water substitute. Subsequently, the cytoplasm was counterstained with eosin Y solution for 1–3 min. Finally, the stained sections were dehydrated through a graded ethanol series, cleared in xylene, and mounted with a synthetic resinous medium.

### 5.23 Multiplex Immunohistochemical (mIHC) Staining

Dewaxing, antigen retrieval, and blocking were performed on paraffin sections of OSCC samples as described above for IHC staining. Sections were then incubated with primary antibodies at 4°C overnight. After incubation with goat anti‐mouse/rabbit HRP‐conjugated secondary antibody (Starter, China) at room temperature for 20 min, HRP‐targeted fluorescent probes (AAT, USA) were applied for another 20 min at room temperature. Sections were washed five times with PBST and heated for antigen retrieval before the next round of primary antibody staining. After all markers were stained, nuclei were counterstained with DAPI for 15 min. Coverslips were mounted with Fluoromount‐G (Beyotime, China). Fluorescence images were acquired using a Zeiss LSM900 microscope and processed with ZEN 1.1.0. For quantification of immune cell infiltration, five randomly selected high‐power fields (40 × magnification) were analyzed for each sample. CD66b^+^PD‐L1^+^ cells and CD8^+^ cells were counted manually and used for statistical analysis. VEGFR‐2 MFI was measured in the corresponding fields. Antibodies are listed in Table S8.

### 5.24 In Vivo Tumorigenicity Assay

Six‐week‐old male C57BL/6 mice were purchased from Shanghai Bikai Keyi Biotechnology Co., Ltd. All mice were bred and housed under specific pathogen‐free conditions in the animal facility of Shanghai Ninth People's Hospital. To evaluate the in vivo role of vegfr‐2 (*kdr*), tongue orthotopic and subcutaneous transplantation models were established. MOC1 cells were engineered to overexpress *kdr* by lentiviral transfection and antibiotic selection, and overexpression was verified by western blotting. Cells were collected during logarithmic growth, digested with trypsin, centrifuged, and resuspended in normal saline. Insulin syringes (KDL) were used to inject 20 µL of cell suspension (200 000 cells; 1 × 10^4^ cells/uL) into the tongue edge or 150 µL of cell suspension (1 × 10^6^ cells; 6.67 × 10^6^ cells/mL) into the backs of mice. Treatment started on day 11. Mouse anti‐PD‐1 antibody (Cat. #A2122, Selleck, USA) was administered intraperitoneally at 10 mg/kg every 3 days. Mouse anti‐GM‐CSF antibody (Cat. #A2148, Selleck, USA) was administered by peritumoral injection at 40 mg/kg every 3 days. The VEGFR‐2 inhibitor cabozantinib/XL‐184 (Cat. #S1119, Selleck, USA) was administered by oral gavage at 25 mg/kg daily. Mouse GM‐CSF was administered by peritumoral injection at 100 µg/kg every 3 days. Mouse anti‐Ly6G antibody was administered intraperitoneally at 10 mg/kg. Tumor volume was calculated as V = 1/2 × length × width^2^. Tumors were harvested on day 20 and were weighed, photographed, fixed, and embedded in paraffin for IHC and multiplex immunofluorescence analysis.

### 5.25 Statistical Analysis

Statistical analyses and data visualization were performed using R Statistical Software and GraphPad Prism 8. Differences among more than two groups were analyzed by one‐way ANOVA, and differences between two groups were analyzed by Student's t‐test. Kaplan‐Meier survival analysis with the log‐rank test was used to compare survival between OSCC patient groups. Receiver operating characteristic (ROC) curves were used to compare tumor tissues and adjacent normal tissues. All *p* values were two‐sided, and *p* < 0.05 was considered statistically significant.

## Author Contributions

Conceptualization: **Fangxing Zhu**, **Yuanhe You**, **Laiping Zhong**, and **Tongchao Zhao**. Methodology: Yuanhe You, **Yiyi Zhang**, and **Zhaoqi Yuan**. Software: Yiyi Zhang. Data curation: **Yibin Dai**. Investigation: **Xinyu Zhou**. Validation: **Yong Fu**, **Xi Lu**, and **Ronghui Xia**. Formal analysis: **Yingying Huang**, **Zhihang Zhou**, and **Wutong Ju**. Supervision: Tongchao Zhao. Funding acquisition: Yong Fu, Laiping Zhong, and Tongchao Zhao. Visualization: Fangxing Zhu and Zhaoqi Yuan. Project administration: Laiping Zhong and Tongchao Zhao. Resources: Yuanhe You, Laiping Zhong, and Tongchao Zhao. Writing – original draft: Fangxing Zhu. Writing – review and editing: Fangxing Zhu, Laiping Zhong, and Tongchao Zhao.

## Acknowlegment

The authors take full responsibility for the content of the manuscript.

## Funding

This work was financially supported, in whole or in part, by Seed Program for Research and Translation of New Medical Technologies of Shanghai Municipal Health Commission (Grant Number: 2025ZZ1032), the National Natural Science Foundation of China (82473268; 82172734), the Excellent Academic Leader Program of Shanghai Oriental Talent Plan (BJWS2024045), the Clinical Scientist Development Program of Fudan University (DGF828024‐2/032), the Shanghai Sailing Program grant (22YF1421700; 24YF2722800), project of Shanghai Huangpu District Science and Technology Commission (No. HLQ202304), the Real‐world research charity project on clinical nutrition management for cancer patients (ZLHZLC‐SH‐2024001), 2025 Shanghai Municipal Health Commission Special Project for Clinical Research in the Health Sector (20254Y0198) the High‐Level Hospital Construction Project of Nanjing Stomatological Hospital, Affiliated Hospital of Medical School, Institute of Stomatology, Nanjing University (No. 0224C019).

## Ethics Statement

This study involving human participants was approved by the Medical Ethics Committee of the Ninth People's Hospital, Shanghai Jiao Tong University School of Medicine (approval No. SH9H‐2019‐T351‐2). All animal experiments were approved by the Institutional Animal Care and Use Committee of the Ninth People's Hospital, Shanghai Jiao Tong University School of Medicine (approval No. SH9H‐2021‐A1048‐SB).

## Conflicts of Interest

The authors declare no conflicts of interest.

## Supporting information




**Supporting File1**: advs76960‐sup‐0001‐SuppMat.docx.

**Supporting File2**: advs76960‐sup‐0002‐FigS1.pdf.

**Supporting File3**: advs76960‐sup‐0003‐FigS2.pdf.

**Supporting File4**: advs76960‐sup‐0004‐FigS3.pdf.

**Supporting File5**: advs76960‐sup‐0005‐FigS4.pdf.

**Supporting File6**: advs76960‐sup‐0006‐FigS5.pdf.

**Supporting File7**: advs76960‐sup‐0007‐FigS6.pdf.

**Supporting File8**: advs76960‐sup‐0008‐FigS7.pdf.

**Supporting File9**: advs76960‐sup‐0009‐FigS8.pdf.

**Supporting File10**: advs76960‐sup‐0010‐FigS9.pdf.

**Supporting File11**: advs76960‐sup‐0011‐FigS10.pdf.

**Supporting File12**: advs76960‐sup‐0012‐FigS11.pdf

**Supporting File13**: advs76960‐sup‐0013‐TableS1.xlsx.

**Supporting File14**: advs76960‐sup‐0014‐TableS2.xlsx.

**Supporting File15**: advs76960‐sup‐0015‐TableS3.xlsx.

**Supporting File16**: advs76960‐sup‐0016‐TableS4.xlsx.

**Supporting File17**: advs76960‐sup‐0017‐TableS5.xlsx.

**Supporting File18**: advs76960‐sup‐0018‐TableS6.xlsx.

**Supporting File19**: advs76960‐sup‐0019‐TableS7.xlsx.

**Supporting File20**: advs76960‐sup‐0020‐TableS8.xlsx.

**Supporting File21**: advs76960‐sup‐0021‐TableS9.xlsx.

## Data Availability

The processed single‐cell RNA‐sequencing data generated in this study have been deposited in the Zenodo database under Digital Object Identifiehttps://doi.org/10.5281/zenodo.21296909. Other datasets supporting the conclusions of this study are available from the corresponding authors upon reasonable request.

## References

[1] A. Miranda‐Filho and F. Bray , “Global Patterns and Trends in Cancers of the Lip, Tongue and Mouth,” Oral Oncology 102 (2020): 104551, 10.1016/j.oraloncology.2019.104551. 31986342

[2] F. Bray , M. Laversanne , H. Sung , et al., “Global Cancer Statistics 2022: GLOBOCAN Estimates of Incidence and Mortality Worldwide for 36 Cancers in 185 Countries,” CA: A Cancer Journal for Clinicians 74, no. 3 (2024): 229–263, 10.3322/caac.21834. 38572751

[3] A. D. Colevas , A. J. Cmelak , D. G. Pfister , et al., “NCCN Guidelines® Insights: Head and Neck Cancers, Version 2.2025,” Journal of the National Comprehensive Cancer Network 23, no. 2 (2025): 2–11, 10.6004/jnccn.2025.0007. 39938471

[4] S. Warnakulasuriya , “Global Epidemiology of Oral and Oropharyngeal Cancer,” Oral Oncology 45, no. 4‐5 (2009): 309–316. 18804401 10.1016/j.oraloncology.2008.06.002

[5] W.‐T. Ju , R.‐H. Xia , D.‐W. Zhu , et al., “A Pilot Study of Neoadjuvant Combination of Anti‐PD‐1 Camrelizumab and VEGFR2 Inhibitor Apatinib for Locally Advanced Resectable Oral Squamous Cell Carcinoma,” Nature Communications 13, no. 1 (2022): 5378, 10.1038/s41467-022-33080-8. PMC9472189 36104359

[6] R. Uppaluri , R. I. Haddad , Y. Tao , et al., “Neoadjuvant and Adjuvant Pembrolizumab in Locally Advanced Head and Neck Cancer,” New England Journal of Medicine 393, no. 1 (2025): 37–50, 10.1056/NEJMoa2415434. 40532178

[7] H. Dong , S. E. Strome , D. R. Salomao , et al., “Tumor‐Associated B7‐H1 Promotes T‐Cell Apoptosis: A Potential Mechanism of Immune Evasion,” Nature Medicine 8, no. 8 (2002): 793–800, 10.1038/nm730. 12091876

[8] T. J. Curiel , S. Wei , H. Dong , et al., “Blockade of B7‐H1 Improves Myeloid Dendritic Cell–Mediated Antitumor Immunity,” Nature Medicine 9, no. 5 (2003): 562–567, 10.1038/nm863. 12704383

[9] B. Burtness , K. J. Harrington , R. Greil , et al., “Pembrolizumab Alone or with Chemotherapy versus cetuximab with Chemotherapy for Recurrent or Metastatic Squamous Cell Carcinoma of the Head and Neck (KEYNOTE‐048): A Randomised, Open‐Label, Phase 3 Study,” The Lancet 394, no. 10212 (2019): 1915–1928, 10.1016/s0140-6736(19)32591-7. 31679945

[10] K. J. Harrington , B. Burtness , R. Greil , et al., “Pembrolizumab with or without Chemotherapy in Recurrent or Metastatic Head and Neck Squamous Cell Carcinoma: Updated Results of the Phase III KEYNOTE‐048 Study,” Journal of Clinical Oncology 41, no. 4 (2023): 790–802, 10.1200/jco.21.02508. 36219809 PMC9902012

[11] K. Kulangara , N. Zhang , E. Corigliano , et al., “Clinical Utility of the Combined Positive Score for Programmed Death Ligand‐1 Expression and the Approval of Pembrolizumab for Treatment of Gastric Cancer,” Archives of Pathology & Laboratory Medicine 143, no. 3 (2019): 330–337, 10.5858/arpa.2018-0043-OA. 30028179

[12] I. Ballesteros , A. Rubio‐Ponce , M. Genua , et al., “Co‐Option of Neutrophil Fates by Tissue Environments,” Cell 183, no. 5 (2020): 1282–1297.e18, 10.1016/j.cell.2020.10.003. 33098771

[13] M. E. Shaul and Z. G. Fridlender , “Tumour‐Associated Neutrophils in Patients with Cancer,” Nature Reviews Clinical Oncology 16, no. 10 (2019): 601–620, 10.1038/s41571-019-0222-4. 31160735

[14] R. Xue , Q. Zhang , Q. Cao , et al., “Liver Tumour Immune Microenvironment Subtypes and Neutrophil Heterogeneity,” Nature 612, no. 7938 (2022): 141–147, 10.1038/s41586-022-05400-x. 36352227

[15] J. Gungabeesoon , N. A. Gort‐Freitas , M. Kiss , et al., “A Neutrophil Response Linked to Tumor Control in Immunotherapy,” Cell 186, no. 7 (2023): 1448–1464.e20, 10.1016/j.cell.2023.02.032. 37001504 PMC10132778

[16] M. S. F. Ng , I. Kwok , L. Tan , et al., “Deterministic Reprogramming of Neutrophils within Tumors,” Science 383, no. 6679 (2024): adf6493, 10.1126/science.adf6493. PMC11087151 38207030

[17] F. Zhu , Y. Zhang , X. Zhou , et al., “dNLR and Tumor Infiltrated Neutrophils Predict the Efficacy of Neoadjuvant Anti‐PD‐1 Camrelizumab and VEGFR2 Inhibitor Apatinib in Locally Advanced Resectable Oral Squamous Cell Carcinoma,” International Journal of Surgery 112 (2026): 1005195.

[18] J. E. Glogauer , C. X. Sun , G. Bradley , and M. A. Magalhaes , “Neutrophils Increase Oral Squamous Cell Carcinoma Invasion through an Invadopodia‐Dependent Pathway,” Cancer Immunology Research 3, no. 11 (2015): 1218–1226, 10.1158/2326-6066.Cir-15-0017. 26112922

[19] X. Wang , M. Cheng , S. Chen , et al., “Resistance to Anti‐LAG‐3 plus Anti‐PD‐1 Therapy in Head and Neck Cancer Is Mediated by Sox^9+^ Tumor Cells Interaction with Fpr1+ Neutrophils,” Nature Communications 16, no. 1 (2025): 3975, 10.1038/s41467-025-59050-4. PMC12037843 40295483

[20] X.‐Y. He , Y. Gao , D. Ng , et al., “Chronic Stress Increases Metastasis via Neutrophil‐Mediated Changes to the Microenvironment,” Cancer Cell 42, no. 3 (2024): 474–486.e12, 10.1016/j.ccell.2024.01.013. 38402610 PMC11300849

[21] T. Zhang , Y. Li , E. Zhai , et al., “Intratumoral Fusobacterium nucleatum Recruits Tumor‐Associated Neutrophils to Promote Gastric Cancer Progression and Immune Evasion,” Cancer Research 85, no. 10 (2025): 1819–1841, 10.1158/0008-5472.Can-24-2580. 39992708 PMC12079103

[22] J. Zhou , Z. Hu , L. Wang , et al., “Tumor‐Colonized Streptococcus Mutans Metabolically Reprograms Tumor Microenvironment and Promotes Oral Squamous Cell Carcinoma. Microbiome,” Microbiome 12, no. 1 (2024): 193, 10.1186/s40168-024-01907-9. 39369210 PMC11452938

[23] M. Uhlén , L. Fagerberg , B. M. Hallström , et al., “Proteomics. Tissue‐Based Map of the human Proteome,” Science 347, no. 6220 (2015): 1260419, 10.1126/science.1260419. 25613900

[24] M. Bartoli , X. Gu , N. T. Tsai , et al., “Vascular Endothelial Growth Factor Activates STAT Proteins in Aortic Endothelial Cells,” Journal of Biological Chemistry 275, no. 43 (2000): 33189–33192, 10.1074/jbc.C000318200. 10961983

[25] Z. Sun , X. Li , S. Massena , et al., “VEGFR2 induces c‐Src Signaling and Vascular Permeability in Vivo via the Adaptor Protein TSAd,” Journal of Experimental Medicine 209, no. 7 (2012): 1363–1377, 10.1084/jem.20111343. 22689825 PMC3405501

[26] Y. Wu , J. Ma , X. Yang , et al., “Neutrophil Profiling Illuminates Anti‐Tumor Antigen‐Presenting Potency,” Cell 187, no. 6 (2024): 1422–1439.e24, 10.1016/j.cell.2024.02.005. 38447573

[27] S. Chen , G. A. Crabill , T. S. Pritchard , et al., “Mechanisms Regulating PD‐L1 Expression on Tumor and Immune Cells,” Journal for ImmunoTherapy of Cancer 7, no. 1 (2019): 305, 10.1186/s40425-019-0770-2. 31730010 PMC6858680

[28] W. Fang , T. Zhou , H. Shi , et al., “Progranulin Induces Immune Escape in Breast Cancer via Up‐Regulating PD‐L1 Expression on Tumor‐Associated Macrophages (TAMs) and Promoting CD8+ T Cell Exclusion,” Journal of Experimental & Clinical Cancer Research 40, no. 1 (2021): 4, 10.1186/s13046-020-01786-6. 33390170 PMC7780622

[29] S. Qin , S. L. Chan , S. Gu , et al., “Camrelizumab plus rivoceranib versus sorafenib as First‐Line Therapy for Unresectable Hepatocellular Carcinoma (CARES‐310): A Randomised, Open‐Label, International Phase 3 Study,” The Lancet 402, no. 10408 (2023): 1133–1146, 10.1016/s0140-6736(23)00961-3. 37499670

[30] E. B. Garon , T.‐E. Ciuleanu , O. Arrieta , et al., “Ramucirumab plus Docetaxel versus Placebo plus Docetaxel for Second‐Line Treatment of Stage IV Non‐Small‐Cell Lung Cancer after Disease Progression on Platinum‐Based Therapy (REVEL): A Multicentre, Double‐Blind, Randomised Phase 3 Trial,” The Lancet 384, no. 9944 (2014): 665–673, 10.1016/s0140-6736(14)60845-x. 24933332

[31] C. S. Fuchs , J. Tomasek , C. J. Yong , et al., “Ramucirumab Monotherapy for Previously Treated Advanced Gastric or Gastro‐Oesophageal Junction Adenocarcinoma (REGARD): An International, Randomised, Multicentre, Placebo‐Controlled, Phase 3 Trial,” The Lancet 383, no. 9911 (2014): 31–39, 10.1016/s0140-6736(13)61719-5. 24094768

[32] C. Meisel , J. C. Schefold , R. Pschowski , et al., “Granulocyte–Macrophage Colony‐Stimulating Factor to Reverse Sepsis‐Associated Immunosuppression: A Double‐Blind, Randomized, Placebo‐Controlled Multicenter Trial,” American Journal of Respiratory and Critical Care Medicine 180, no. 7 (2009): 640–648, 10.1164/rccm.200903-0363OC. 19590022

[33] E. M. Pinder , A. J. Rostron , T. P. Hellyer , et al., “Randomised Controlled Trial of GM‐CSF in Critically Ill Patients with Impaired Neutrophil Phagocytosis,” Thorax 73, no. 10 (2018): 918–925, 10.1136/thoraxjnl-2017-211323. 30064991 PMC6166597

[34] F. Wimmers , G. Schreibelt , and A. E. Sköld , “Paradigm Shift in Dendritic Cell‐Based Immunotherapy: From in Vitro Generated Monocyte‐Derived DCs to Naturally Circulating DC Subsets,” Frontiers in Immunology 5 (2014): 165, 10.3389/fimmu.2014.00165. 24782868 PMC3990057

[35] R. A. Burga , M. Thorn , G. R. Point , et al., “Liver Myeloid‐Derived Suppressor Cells Expand in Response to Liver Metastases in Mice and Inhibit the Anti‐Tumor Efficacy of Anti‐CEA CAR‐T,” Cancer Immunology, Immunotherapy 64, no. 7 (2015): 817–829, 10.1007/s00262-015-1692-6. 25850344 PMC4485571

[36] G. Kohanbash , K. McKaveney , M. Sakaki , et al., “GM‐CSF Promotes the Immunosuppressive Activity of Glioma‐Infiltrating Myeloid Cells through Interleukin‐4 Receptor‐α,” Cancer Research 73, no. 21 (2013): 6413–6423, 10.1158/0008-5472.Can-12-4124. 24030977 PMC3829000

[37] L. Codarri , G. Gyülvészi , V. Tosevski , et al., “RORγt Drives Production of the Cytokine GM‐CSF in Helper T Cells, Which Is Essential for the Effector Phase of Autoimmune Neuroinflammation,” Nature Immunology 12, no. 6 (2011): 560–567, 10.1038/ni.2027. 21516112

[38] Y. Wang , G. Han , K. Wang , et al., “Tumor‐Derived GM‐CSF Promotes Inflammatory Colon Carcinogenesis via Stimulating Epithelial Release of VEGF,” Cancer Research 74, no. 3 (2014): 716–726, 10.1158/0008-5472.Can-13-1459. 24366884

[39] X. Su , Y. Xu , G. C. Fox , et al., “Breast Cancer–derived GM‐CSF Regulates Arginase 1 in Myeloid Cells to Promote an Immunosuppressive Microenvironment,” Journal of Clinical Investigation 131, no. 20 (2021): 145296, 10.1172/jci145296. PMC8516467 34520398

[40] X. Zhu , Y. Heng , J. Ma , et al., “Prolonged Survival of Neutrophils Induced by Tumor‐Derived G‐CSF/GM‐CSF Promotes Immunosuppression and Progression in Laryngeal Squamous Cell Carcinoma,” Advanced Science 11 (2024): 2400836, 10.1002/advs.202400836. 39447112 PMC11633501

[41] J. Xiao , S. Wang , L. Chen , et al., “25‐Hydroxycholesterol Regulates Lysosome AMP Kinase Activation and Metabolic Reprogramming to Educate Immunosuppressive Macrophages,” Immunity 57, no. 5 (2024): 1087–1104.e7, 10.1016/j.immuni.2024.03.021. 38640930

[42] H. Chen , H. Sun , F. You , et al., “Activation of STAT6 by STING Is Critical for Antiviral Innate Immunity,” Cell 147, no. 2 (2011): 436–446, 10.1016/j.cell.2011.09.022. 22000020

[43] I. Ozel , G. Sha , A. Będzińska , et al., “Neutrophil‐Specific Targeting of STAT3 Impairs Tumor Progression via the Expansion of Cytotoxic CD8(+) T Cells,” Signal Transduction and Targeted Therapy 10, no. 1 (2025): 279, 10.1038/s41392-025-02363-z. 40885734 PMC12398498

[44] J. Wang , Y. Jia , N. Wang , et al., “The Clinical Significance of Tumor‐Infiltrating Neutrophils and Neutrophil‐To‐CD8+ Lymphocyte Ratio in Patients with Resectable Esophageal Squamous Cell Carcinoma,” Journal of Translational Medicine 12, no. 1 (2014): 7, 10.1186/1479-5876-12-7. 24397835 PMC3895663

[45] J.‐F. Wang , Y.‐P. Wang , J. Xie , et al., “Upregulated PD‐L1 Delays Human Neutrophil Apoptosis and Promotes Lung Injury in an Experimental Mouse Model of Sepsis,” Blood 138, no. 9 (2021): 806–810, 10.1182/blood.2020009417. 34473230

[46] I. Korsunsky , N. Millard , J. Fan , et al., “Fast, Sensitive and Accurate Integration of Single‐Cell Data with Harmony,” Nature Methods 16, no. 12 (2019): 1289–1296, 10.1038/s41592-019-0619-0. 31740819 PMC6884693

[47] Z. Yuan , Z. Zhu , F. Zhu , et al., “Impact of human Adipose Tissue‐Derived Stem Cells on Dermatofibrosarcoma Protuberans Cells in an Indirect co‐Culture: An in Vitro Study,” Stem Cell Research & Therapy 12, no. 1 (2021): 440, 10.1186/s13287-021-02512-5. 34362454 PMC8344160

