# Supplementary material for: GM‐CSF Promotes Neutrophil PD‐L1 Expression Through the VEGFR‐2/STAT6/CSF2 Signaling Axis in Oral Squamous Cell Carcinoma
Source: Adv Sci (Weinh). 2026 Aug 6;13(60):e76960. doi: 10.1002/advs.76960 (PMC13445316; doi:10.1002/advs.76960)

Corresponding to Supplementary Fig. S5B

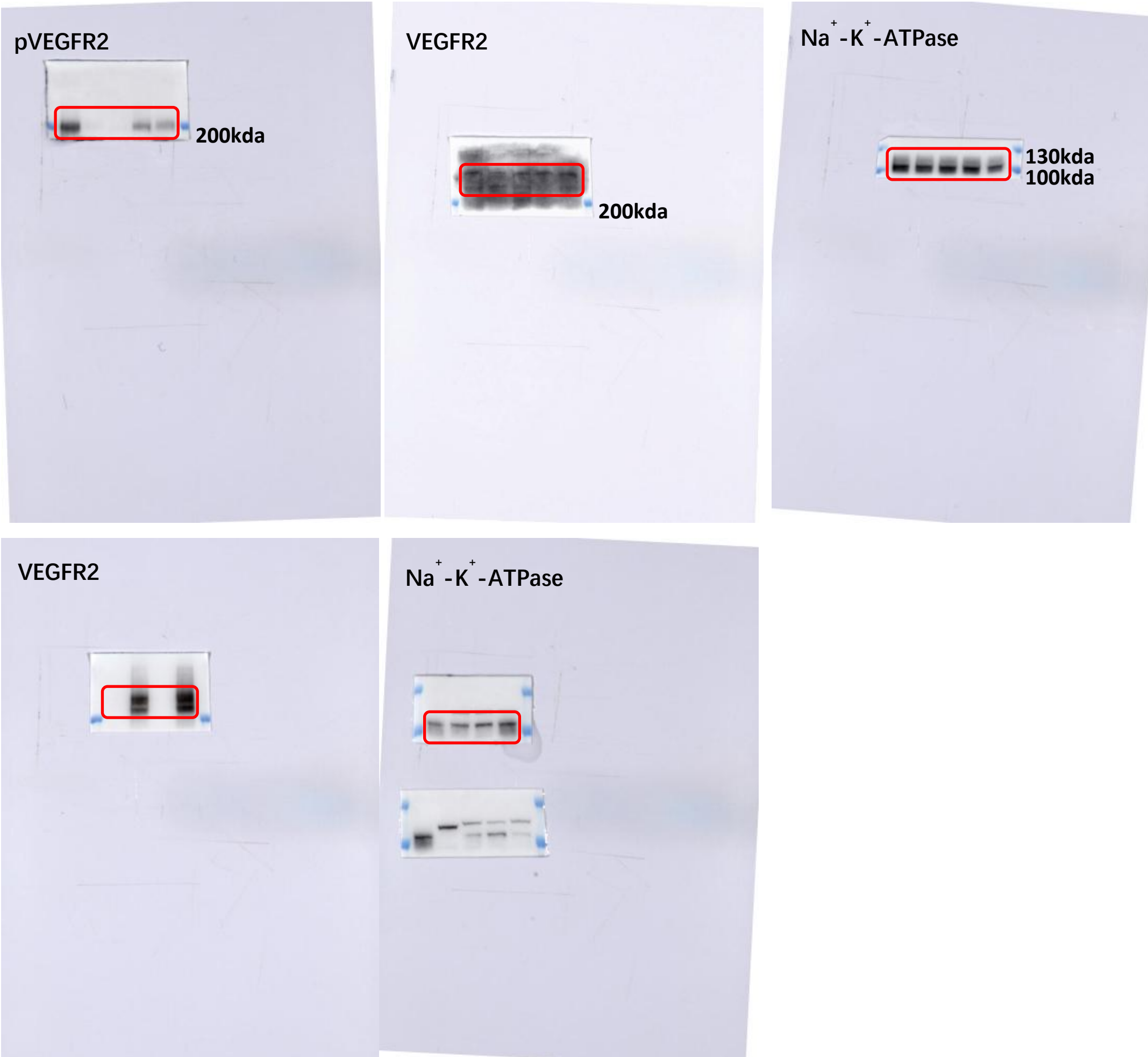

Corresponding to Supplementary Fig. S5C

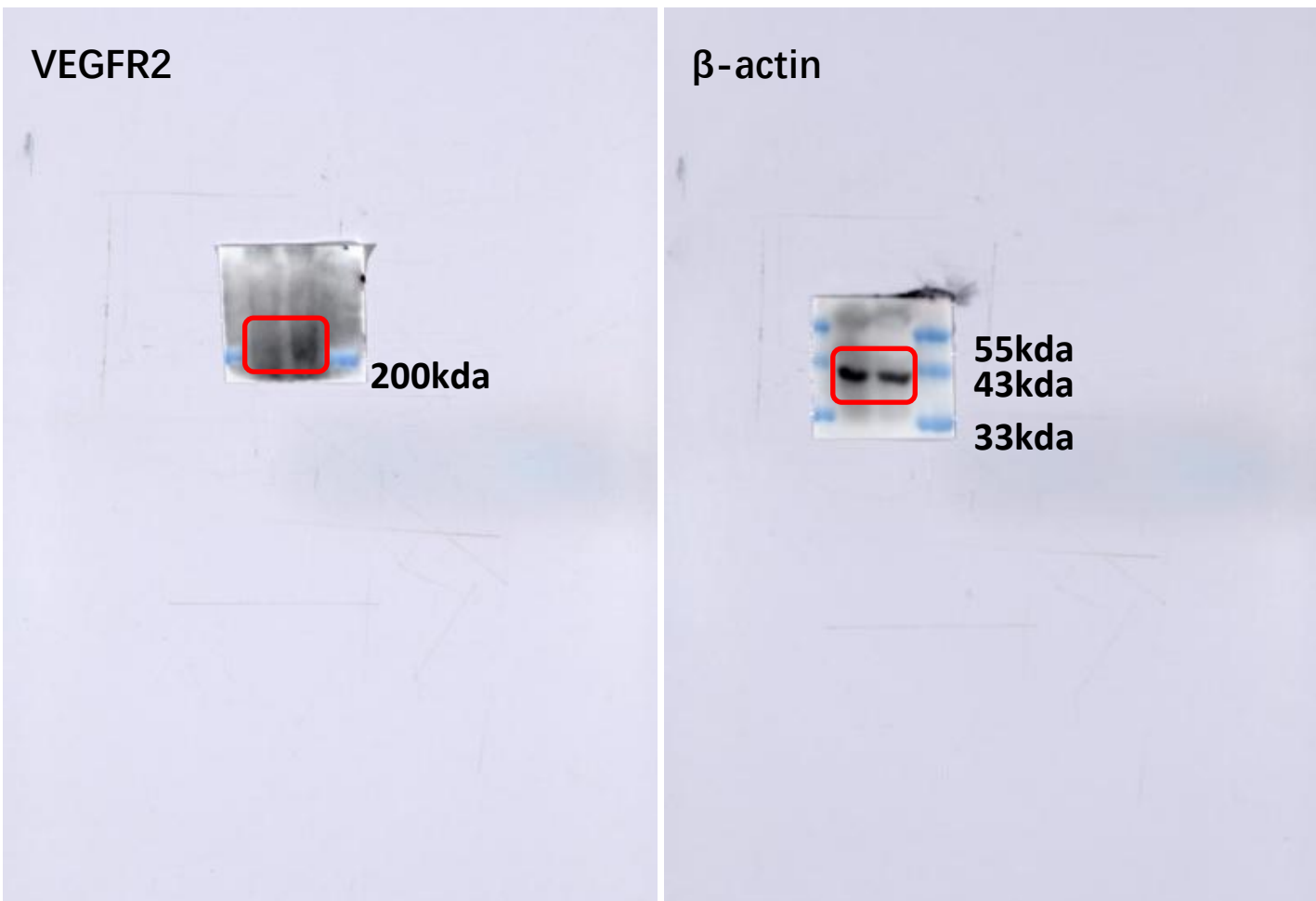

Corresponding to Fig. 3A

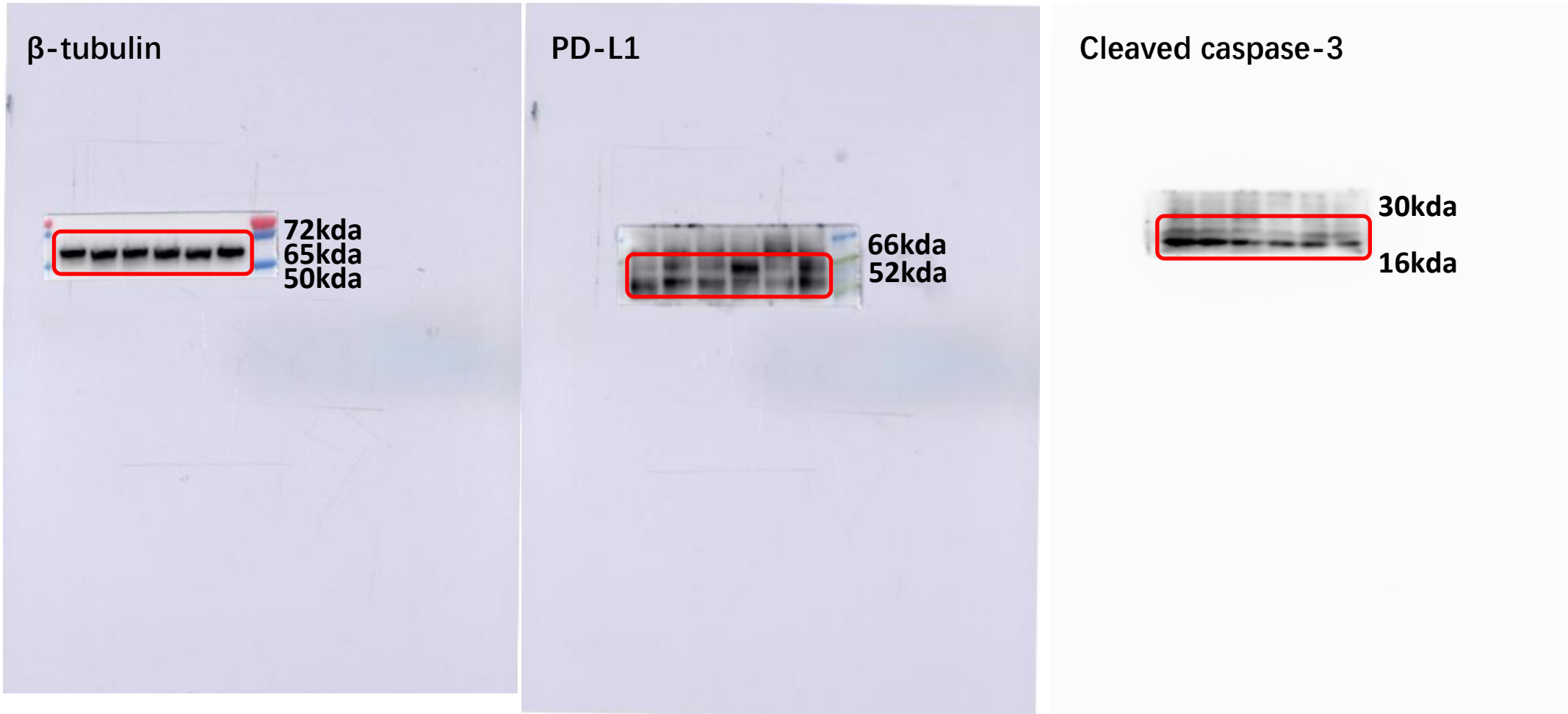

Corresponding to Fig. 4A

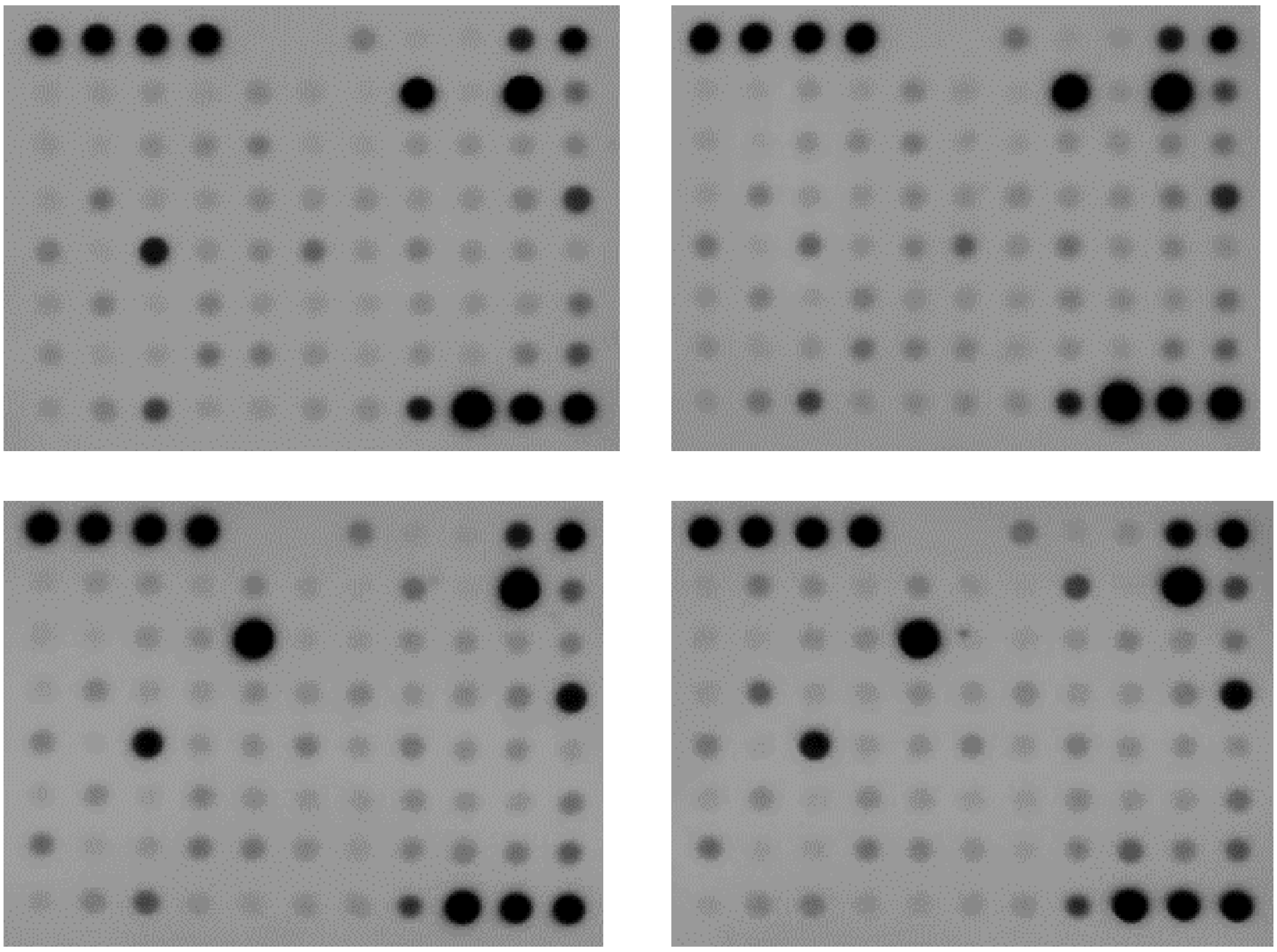

Corresponding to Fig. 4D

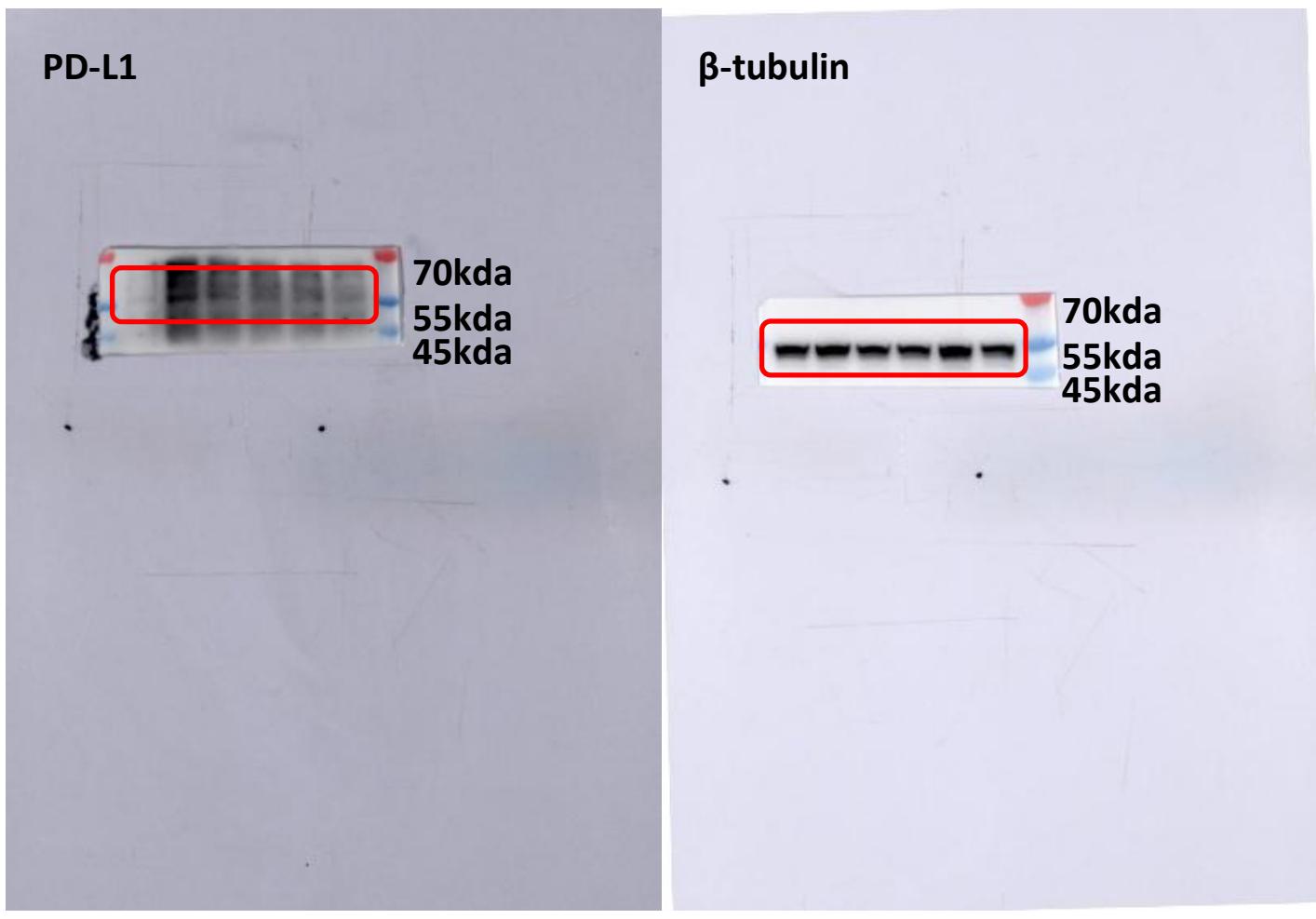

Corresponding to Fig. 4H left

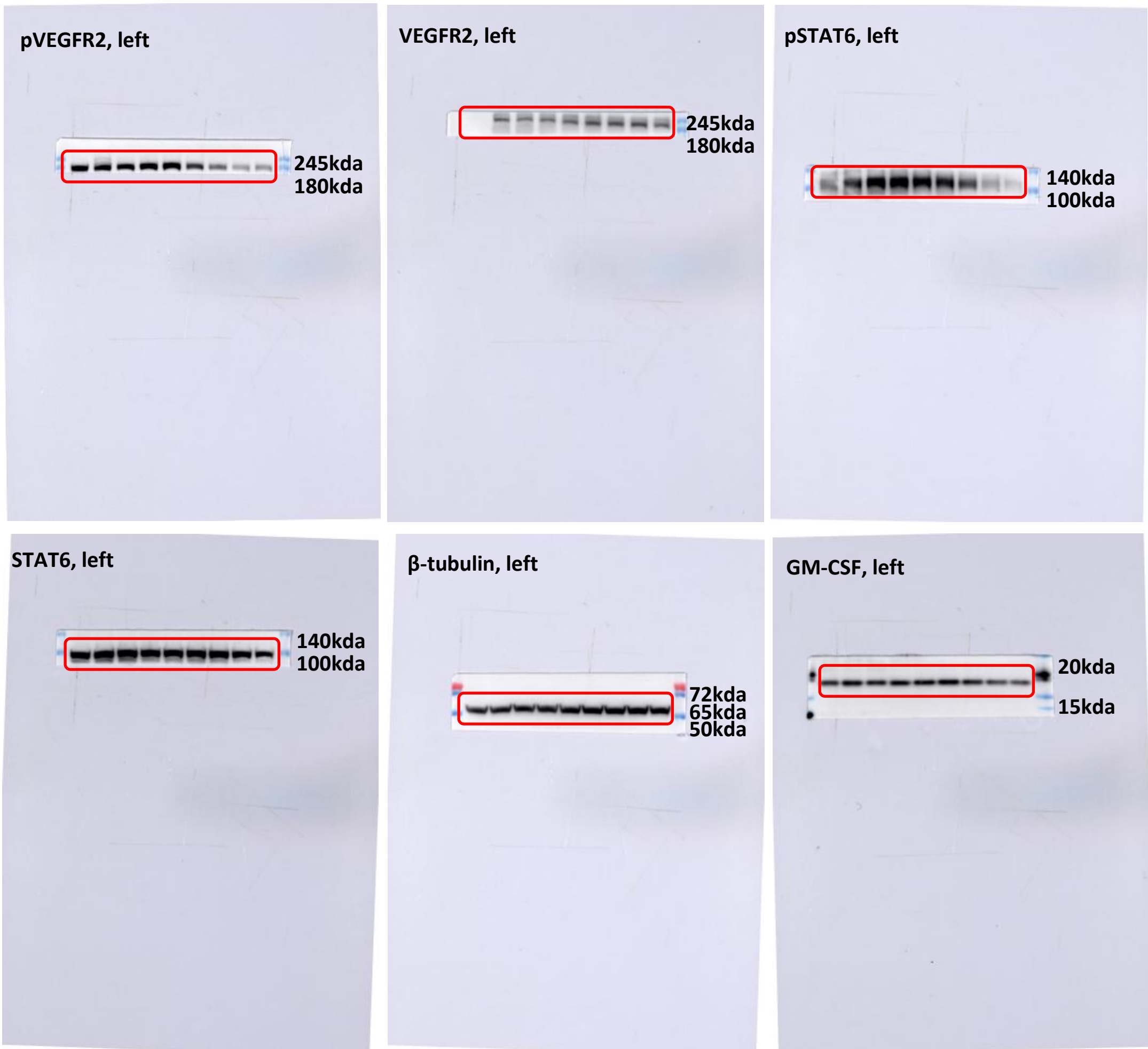

Corresponding to Fig. 4H right

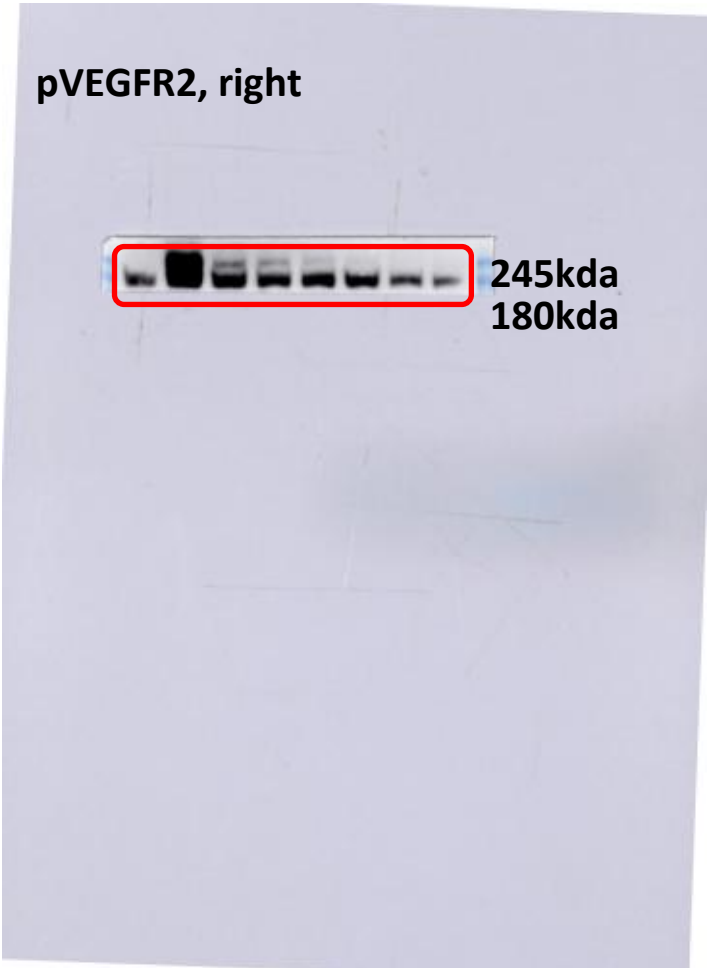

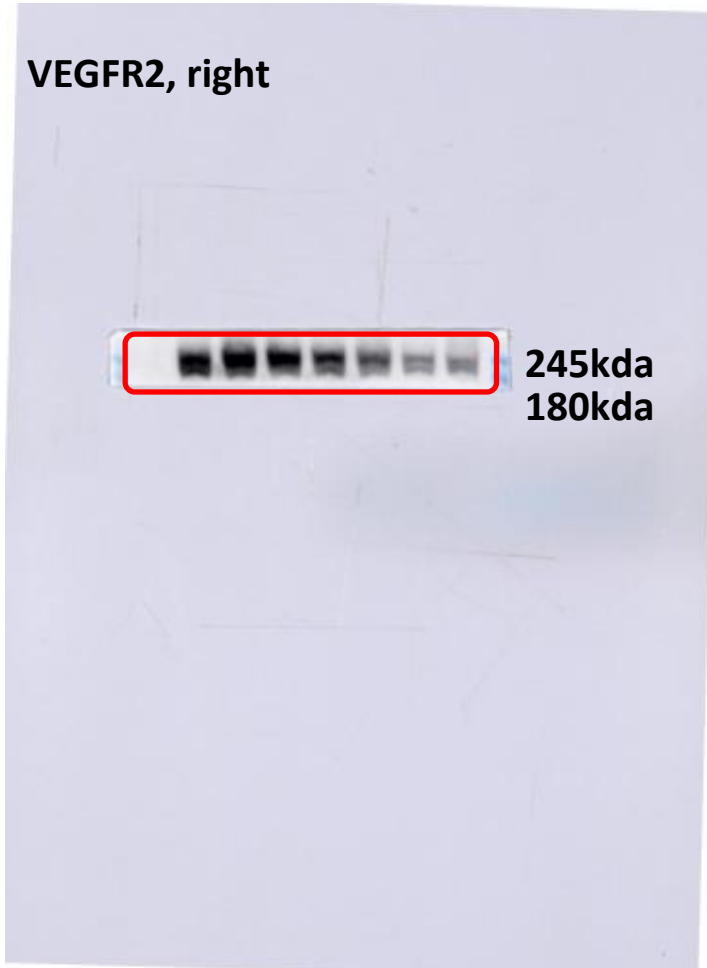

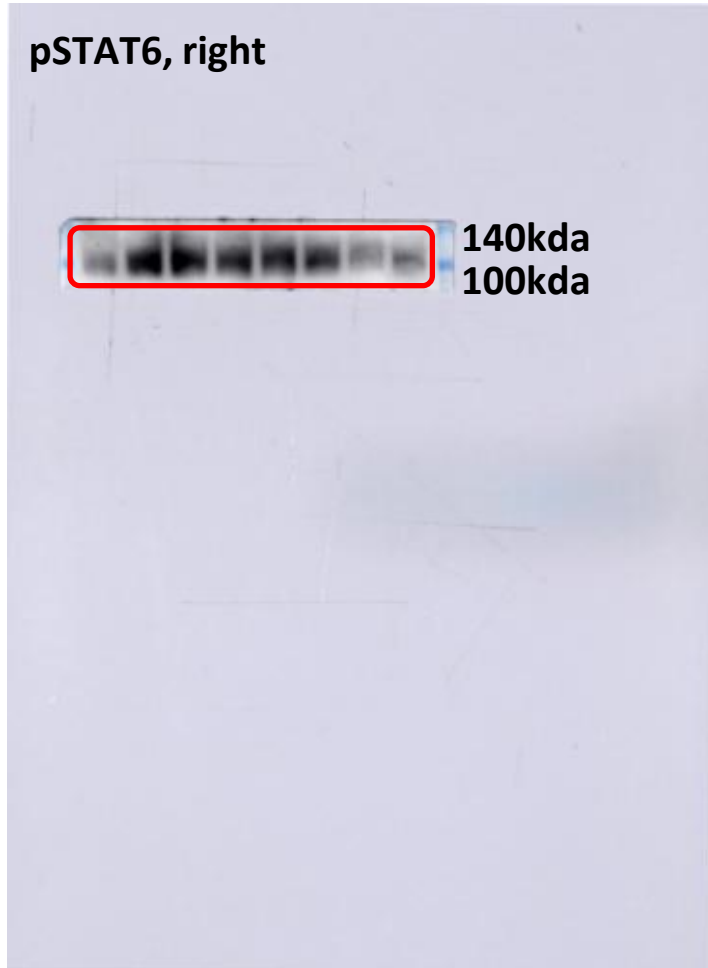

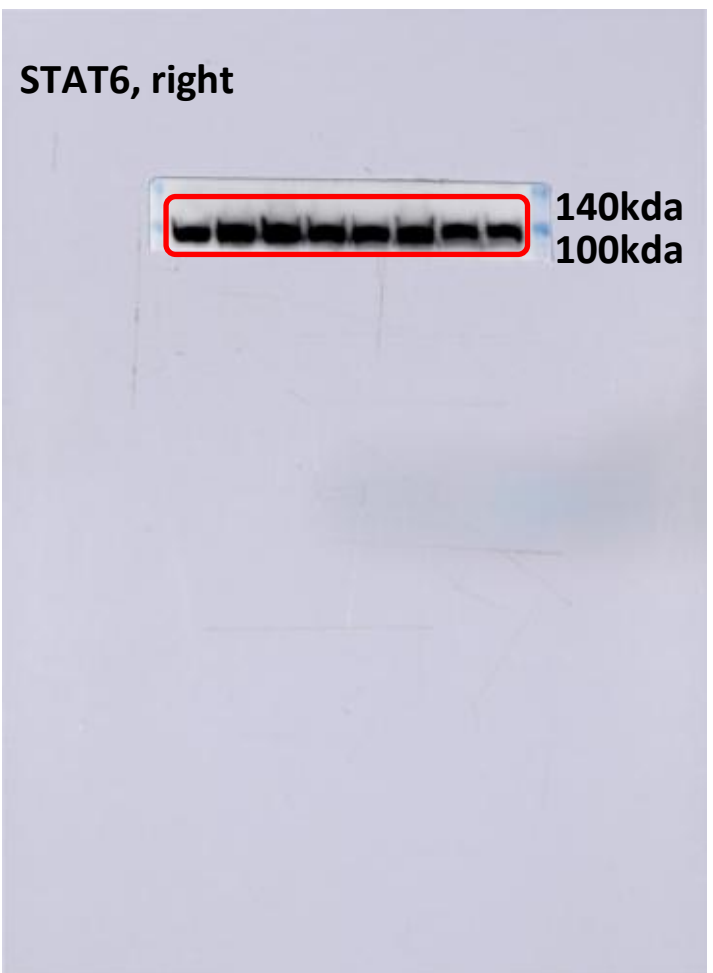

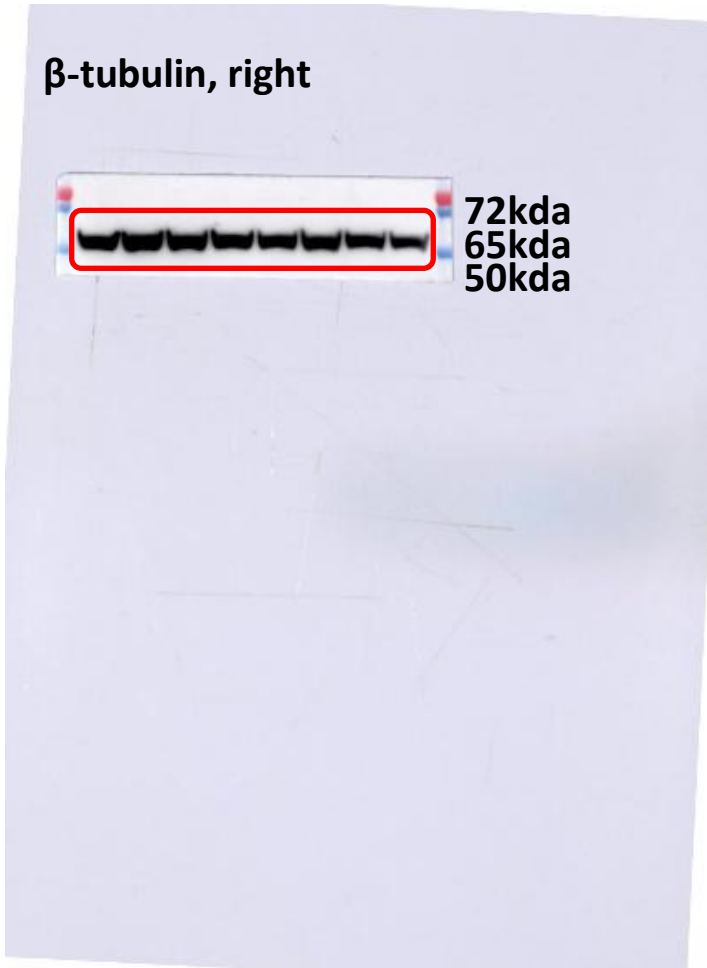

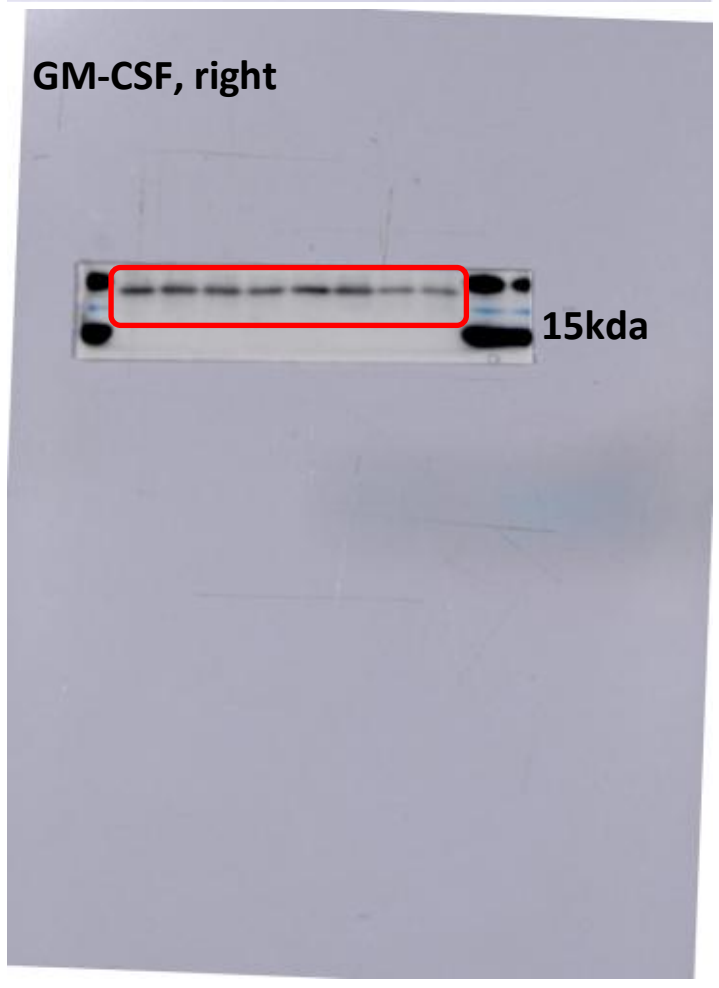

Corresponding to Supplementary Fig. S6A

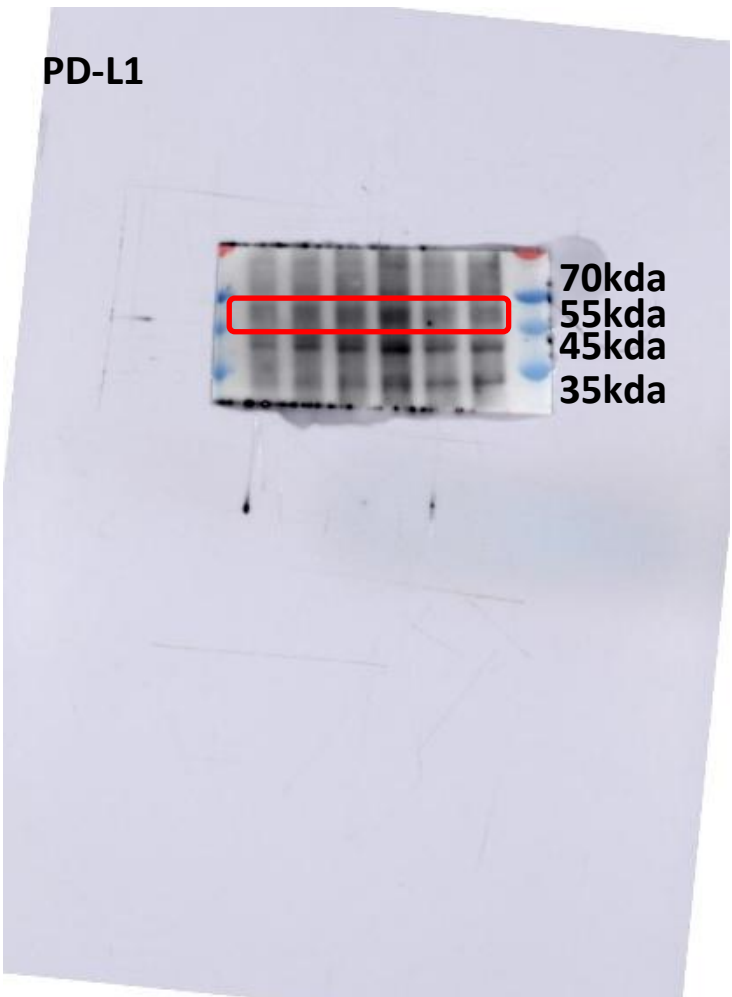

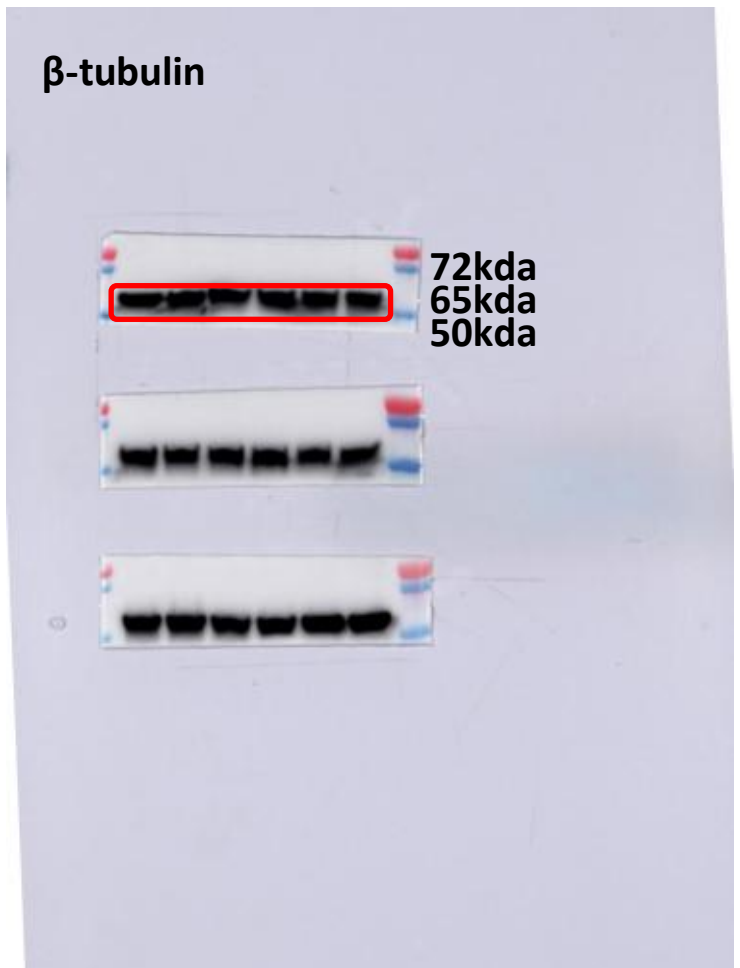

Corresponding to Supplementary Fig. S6B

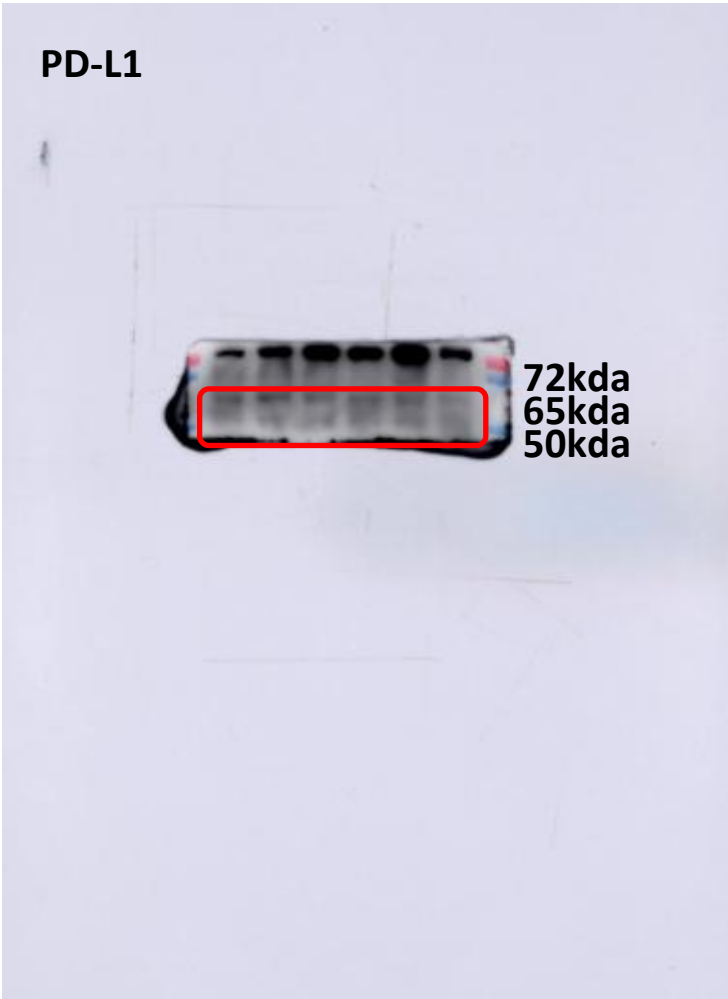

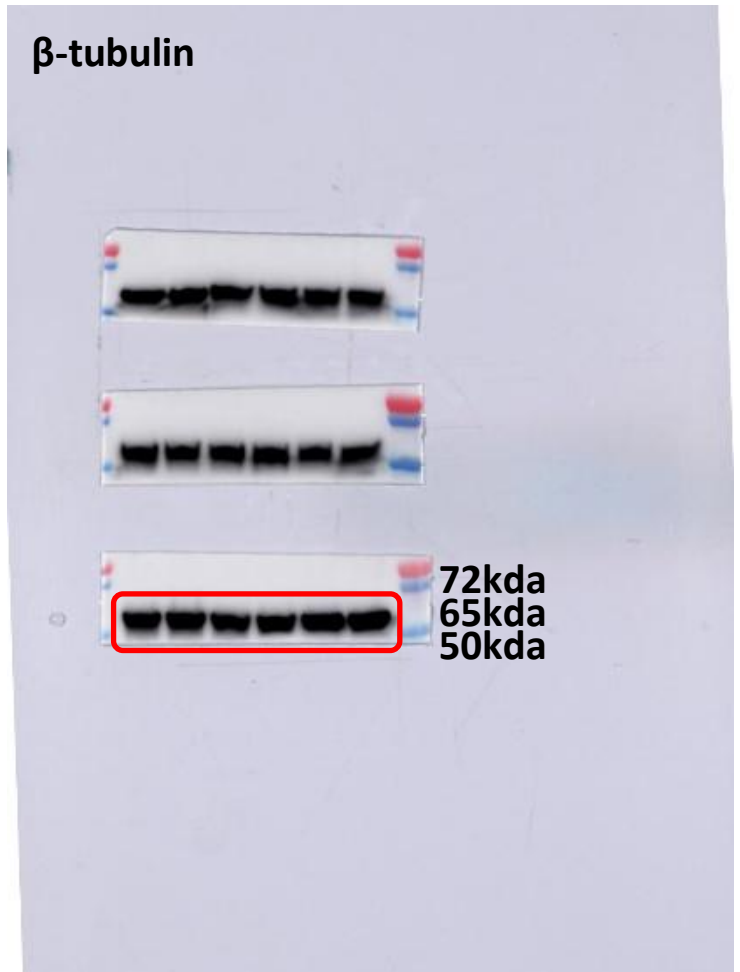

Corresponding to Supplementary Fig. S6C

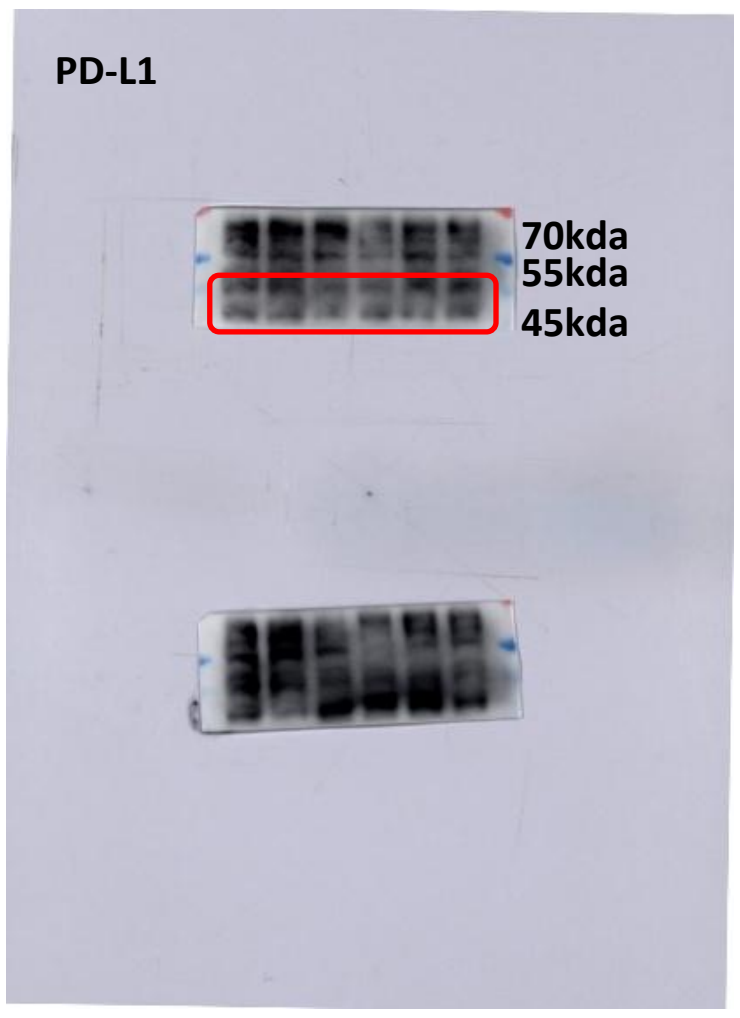

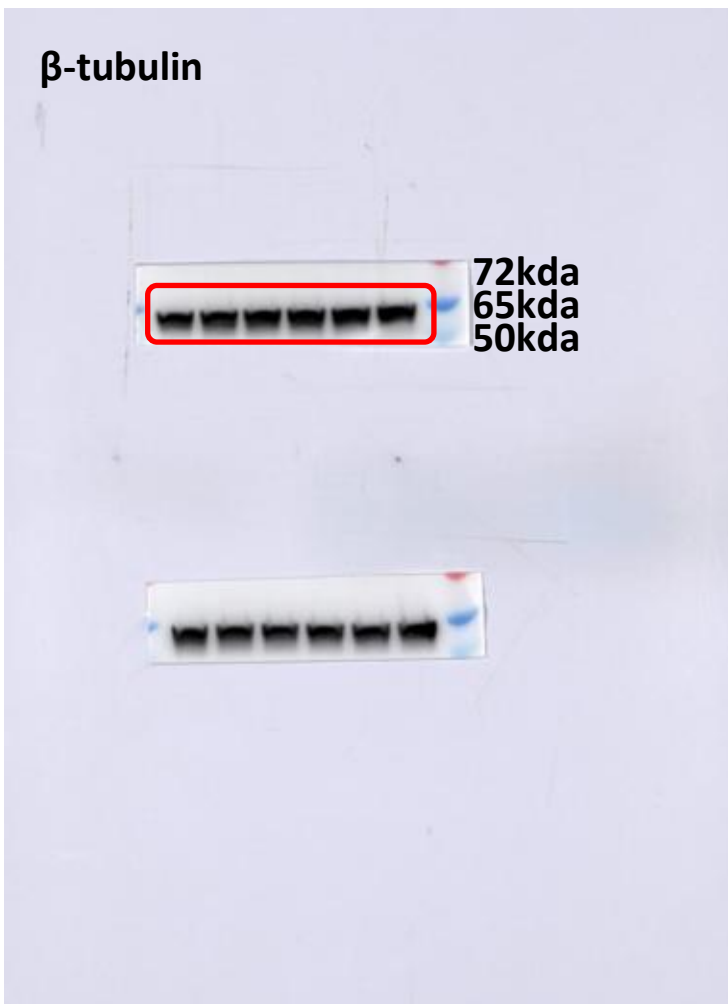

Corresponding to Supplementary Fig. S6D

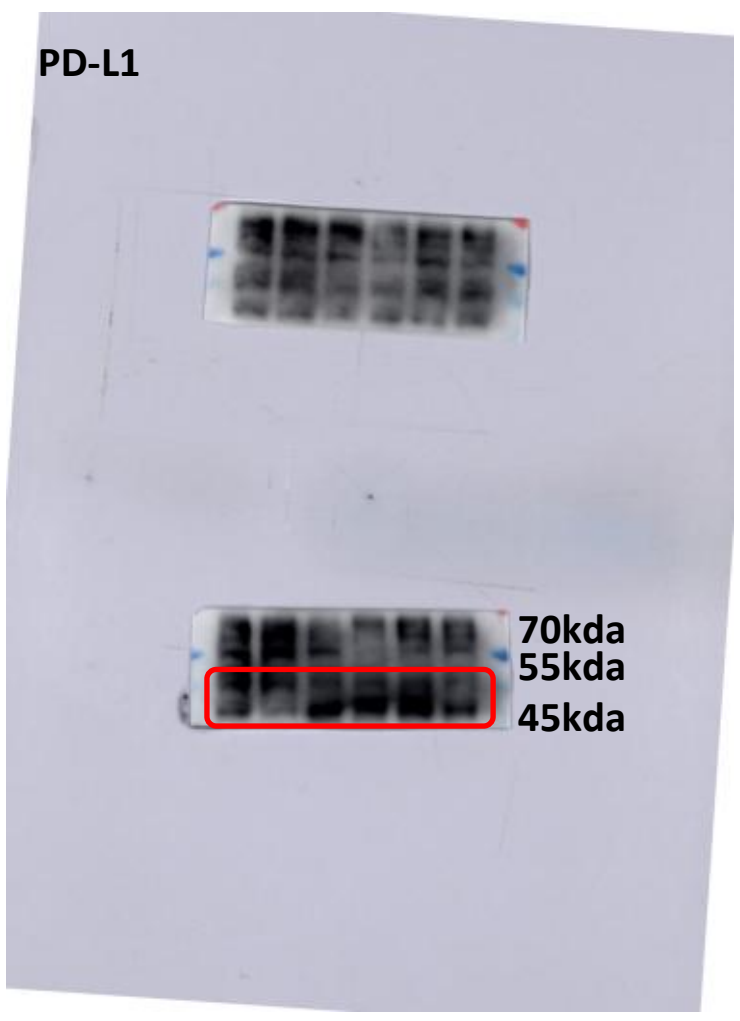

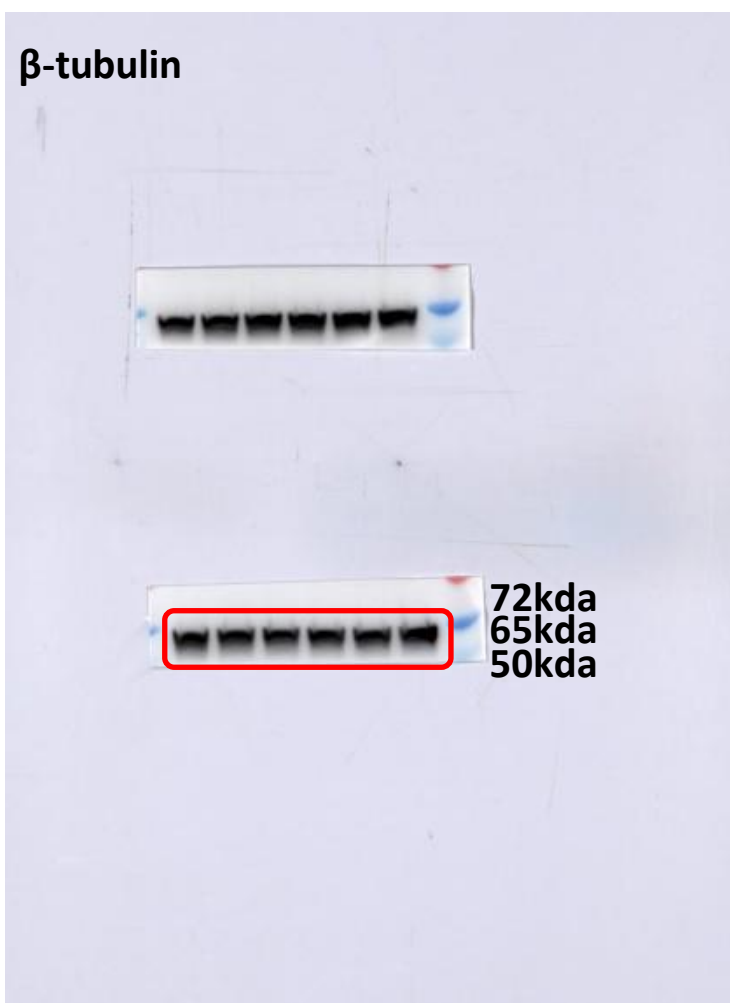

Corresponding to Supplementary Fig. S6E

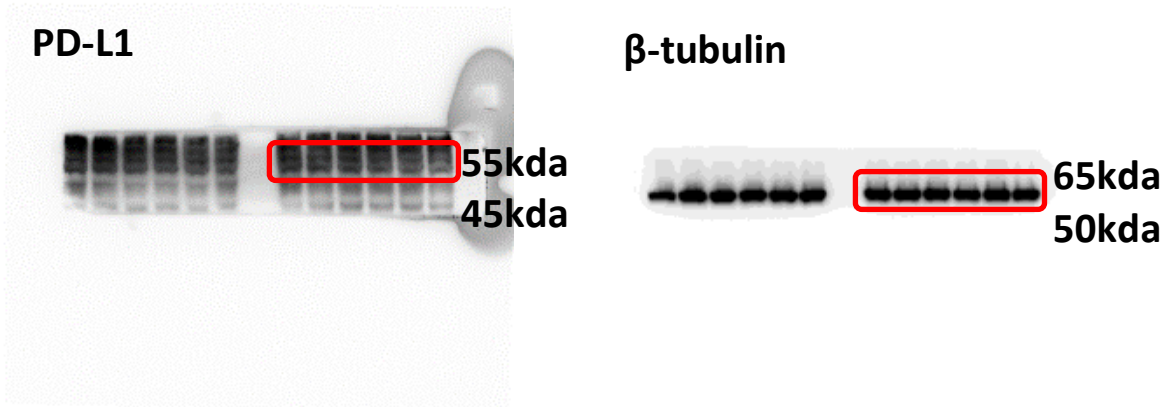

Corresponding to Fig. 5D

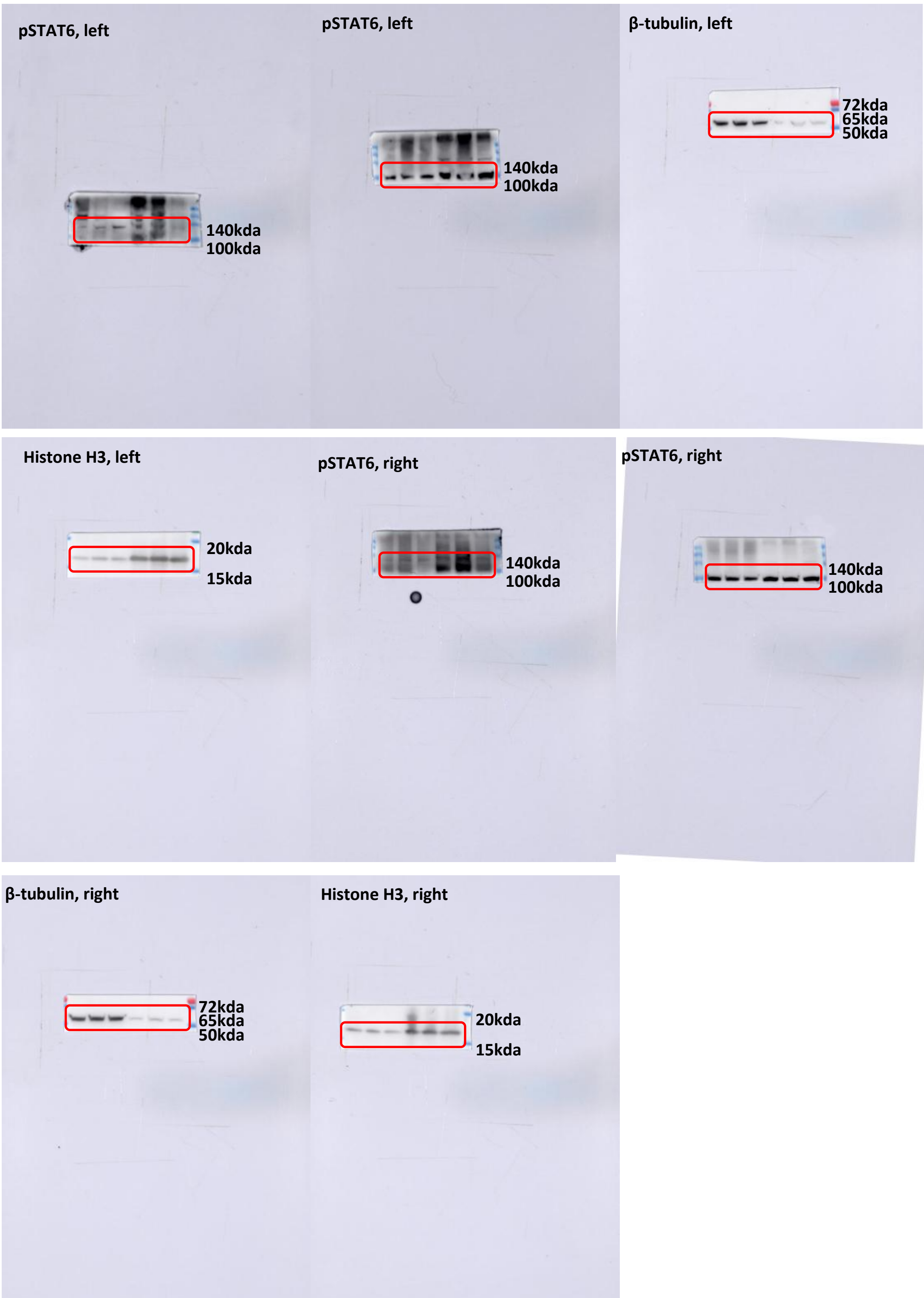

Corresponding to Fig. 5G

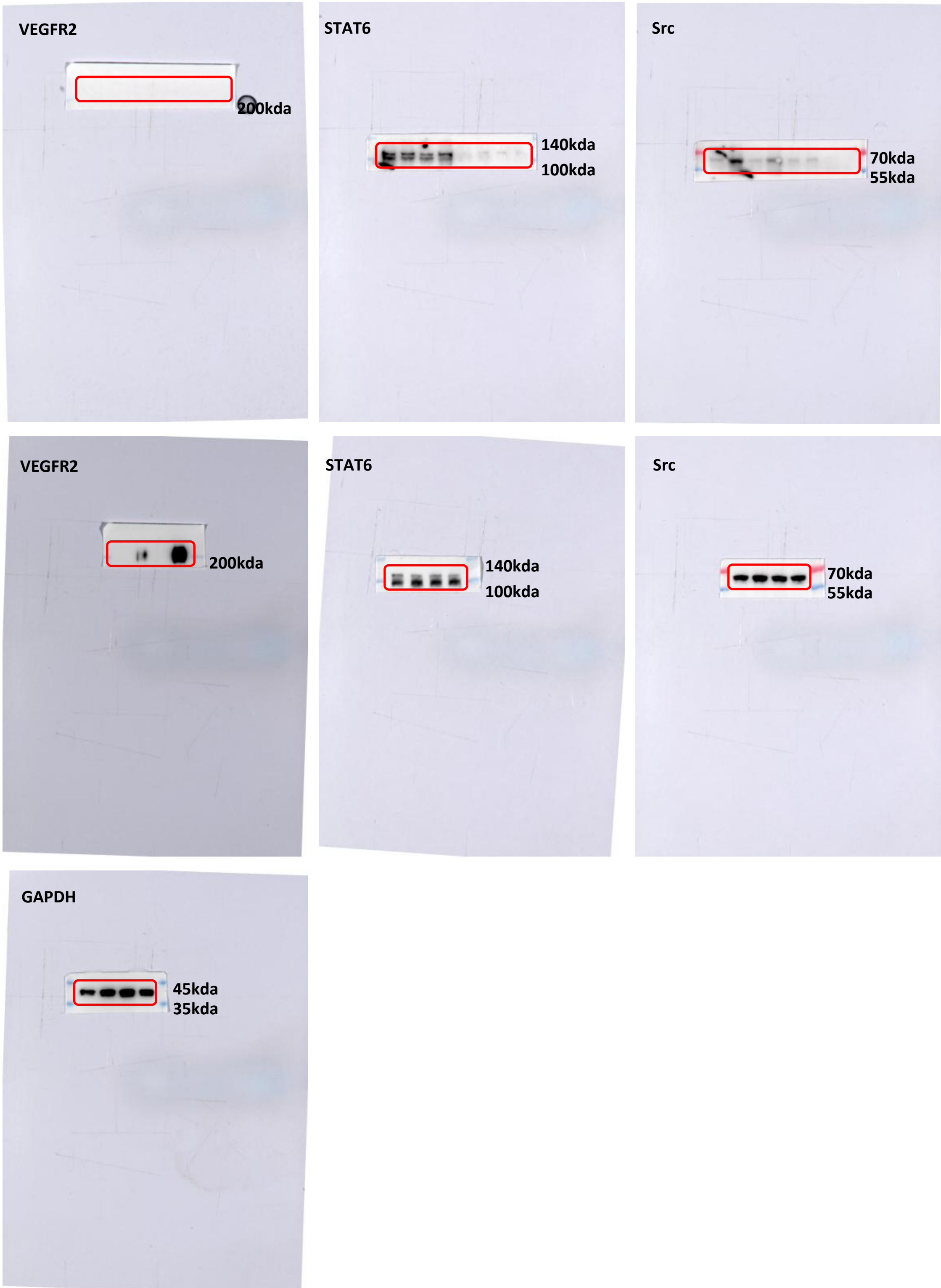

Corresponding to Fig. 5H

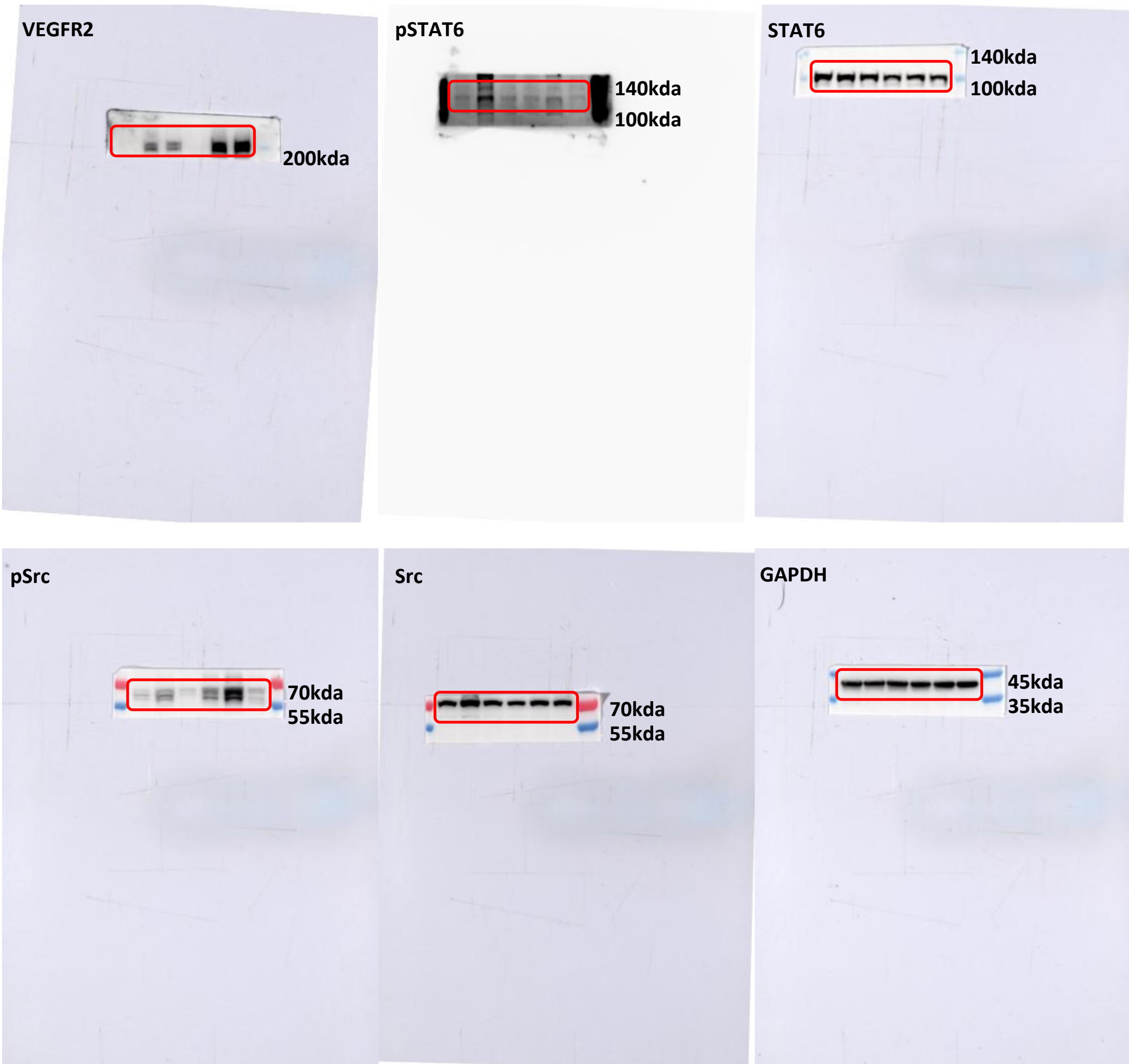

Corresponding to Supplementary Fig. S7C

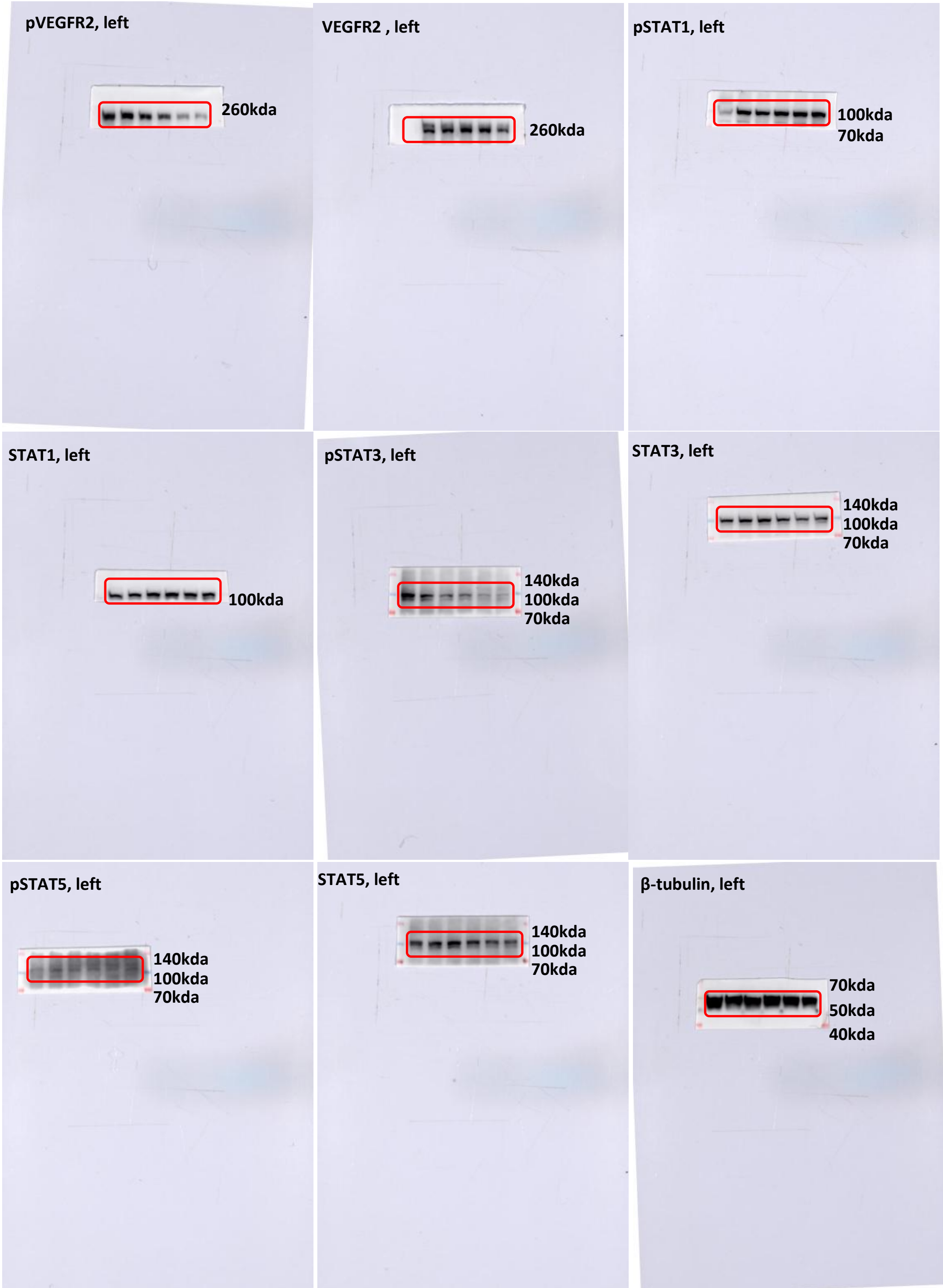

Corresponding to Supplementary Fig. S7C

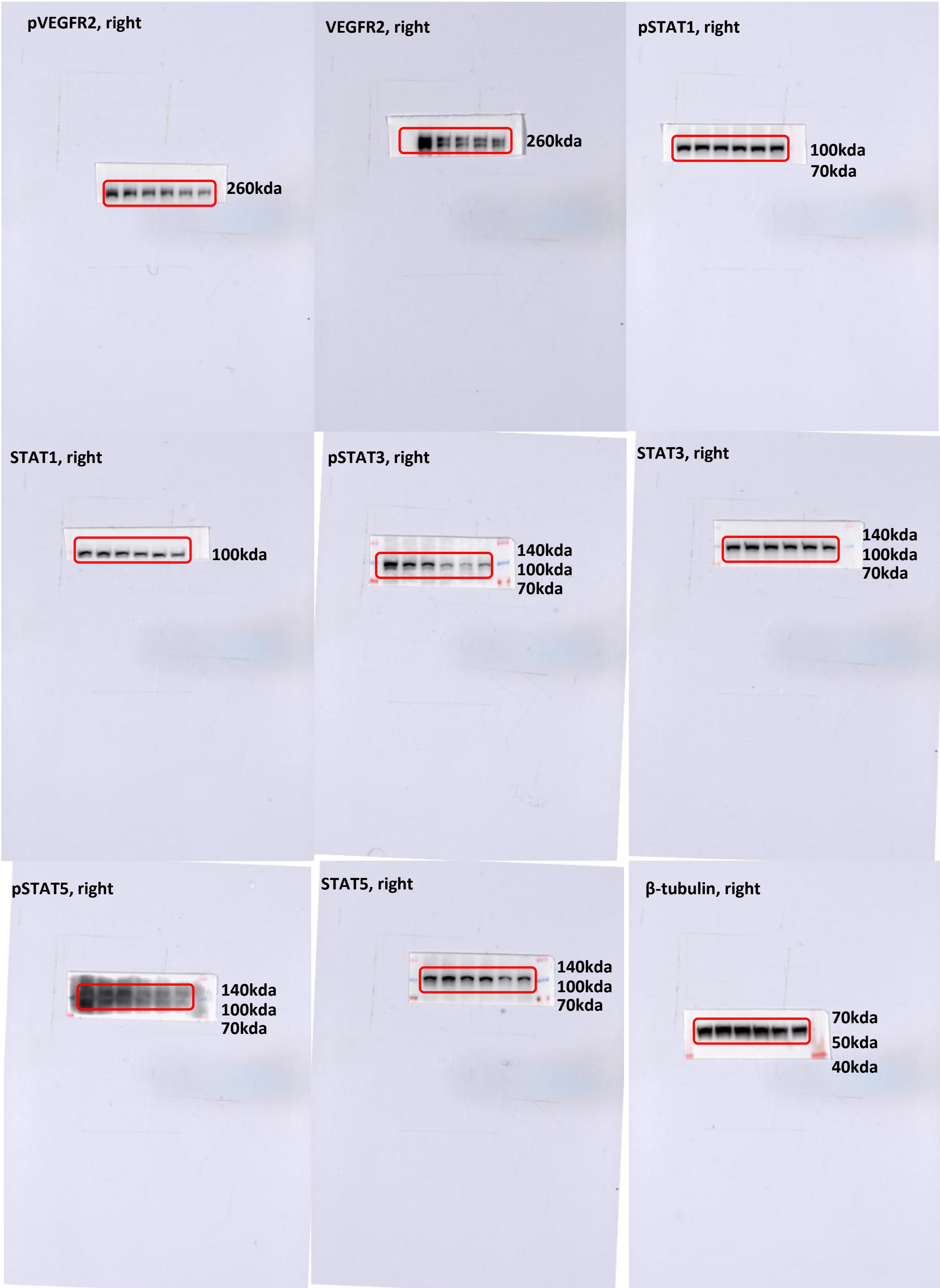

Corresponding to Supplementary Fig. S7D

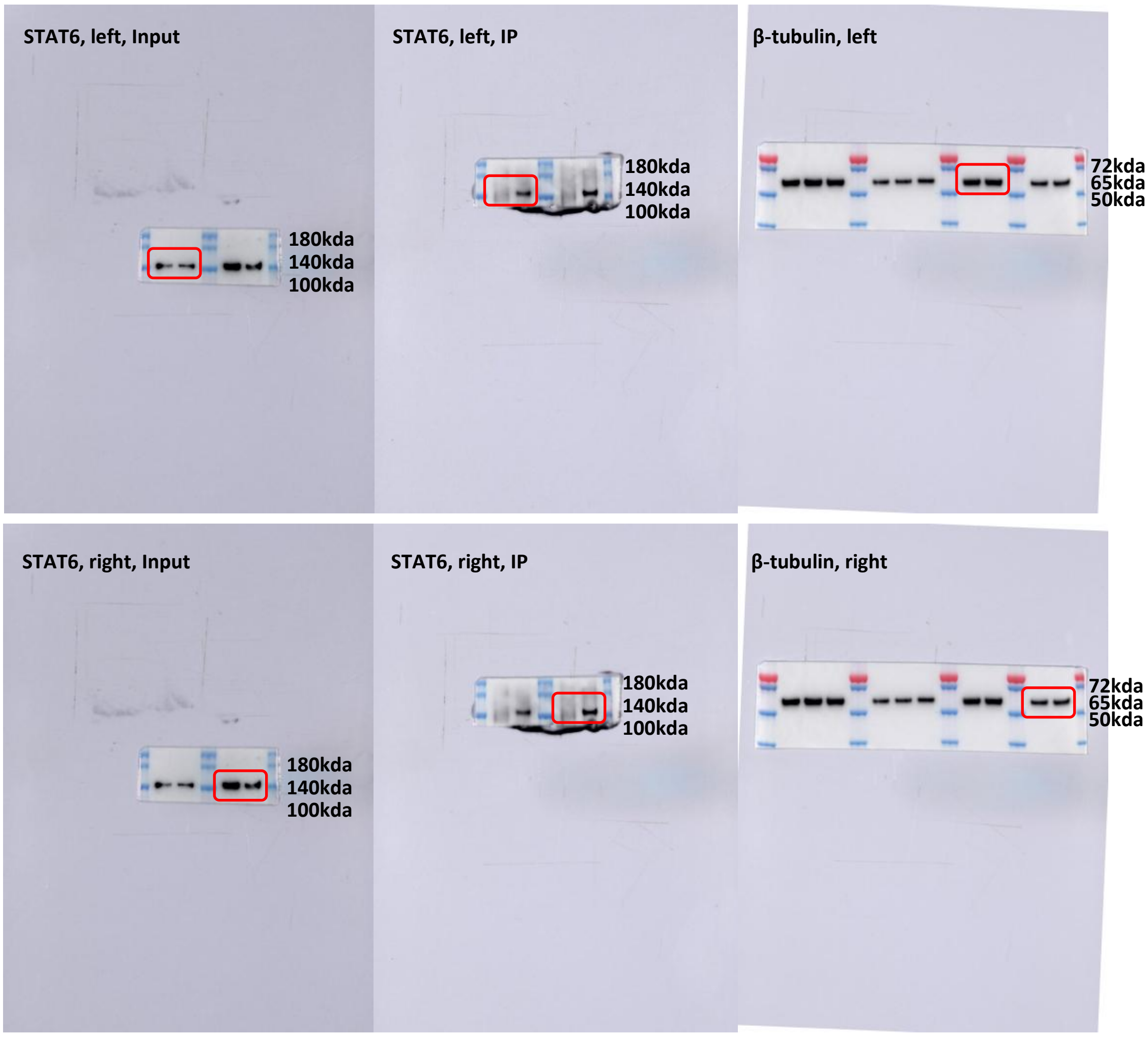

Corresponding to Supplementary Fig. S7E

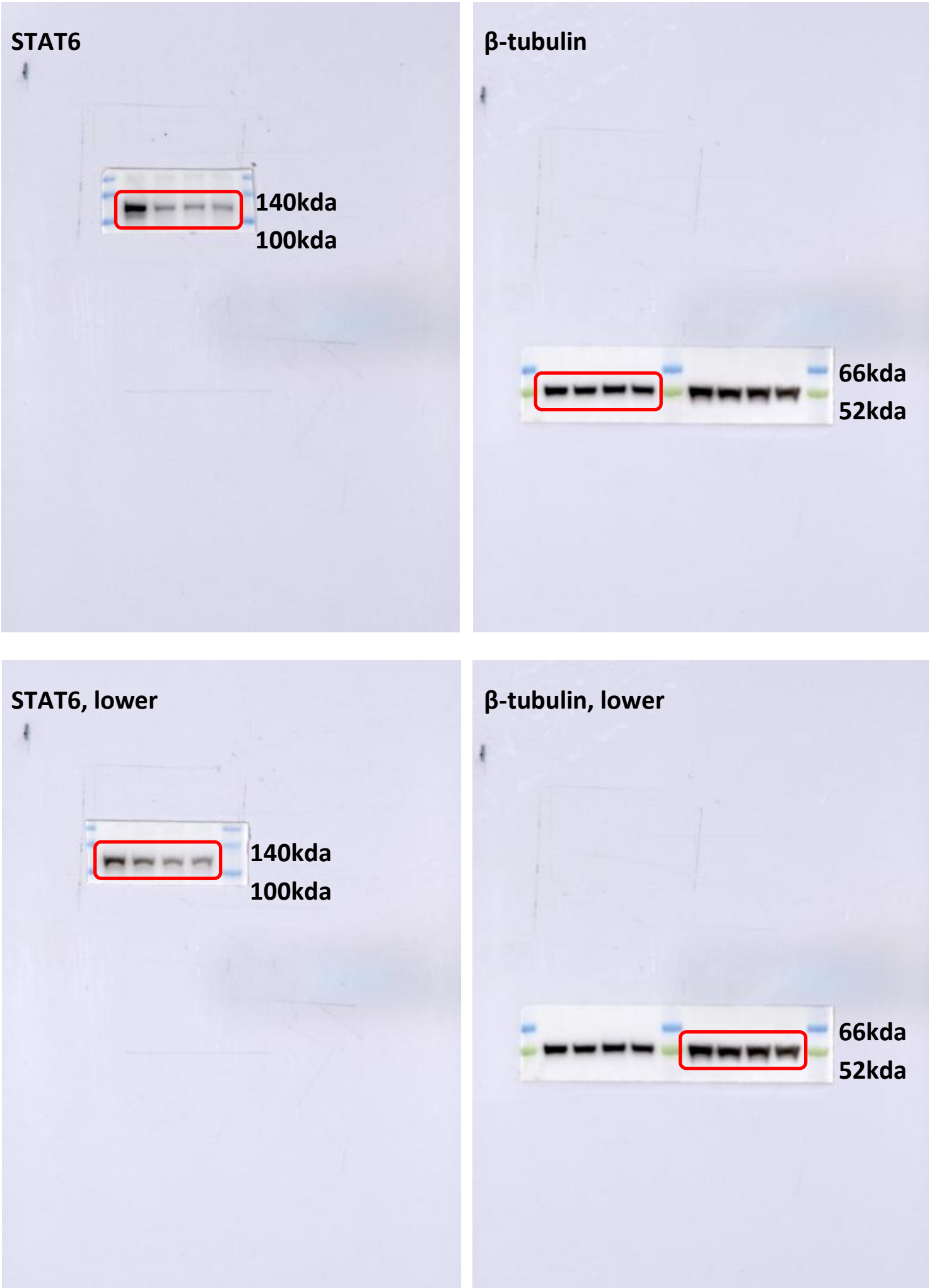

Corresponding to Supplementary Fig. S7F

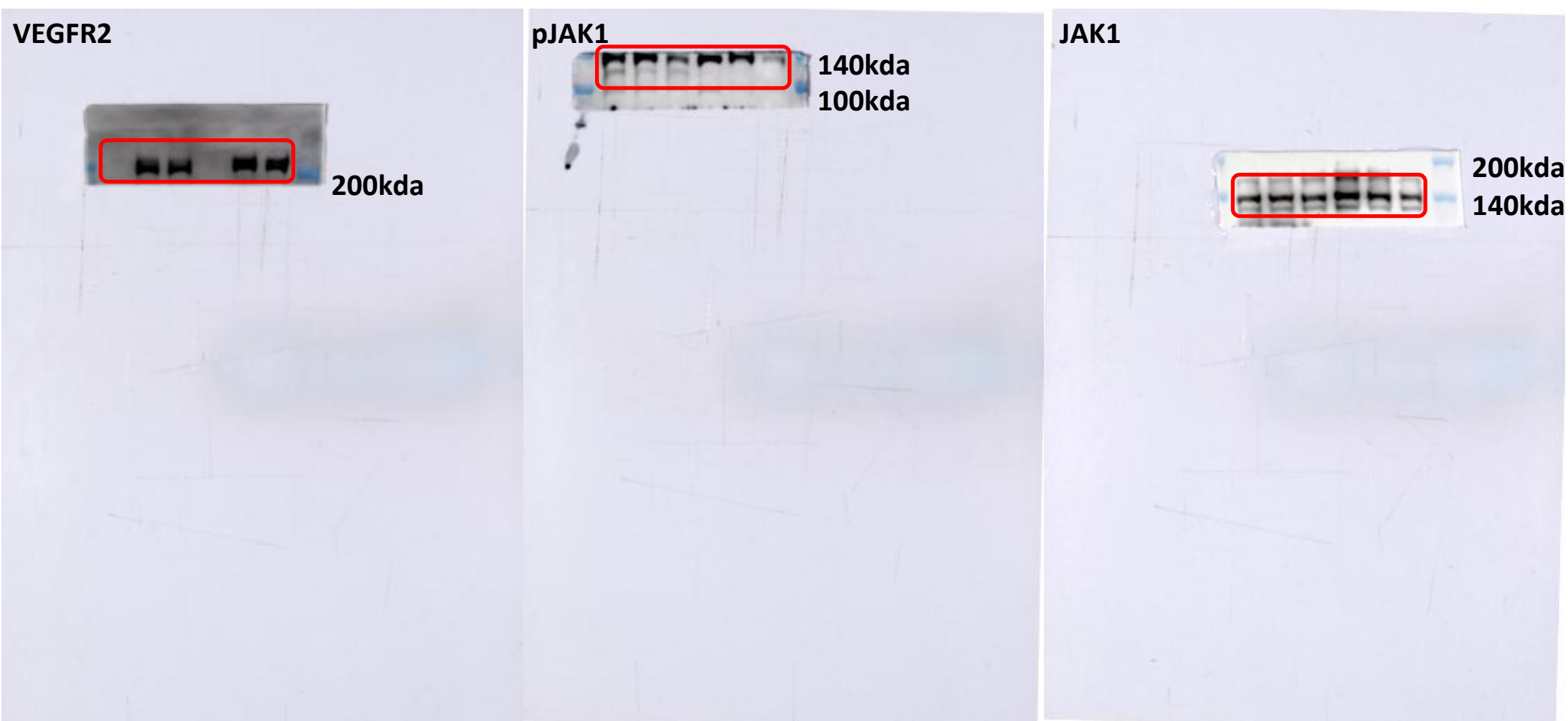

Corresponding to Supplementary Fig. S7F

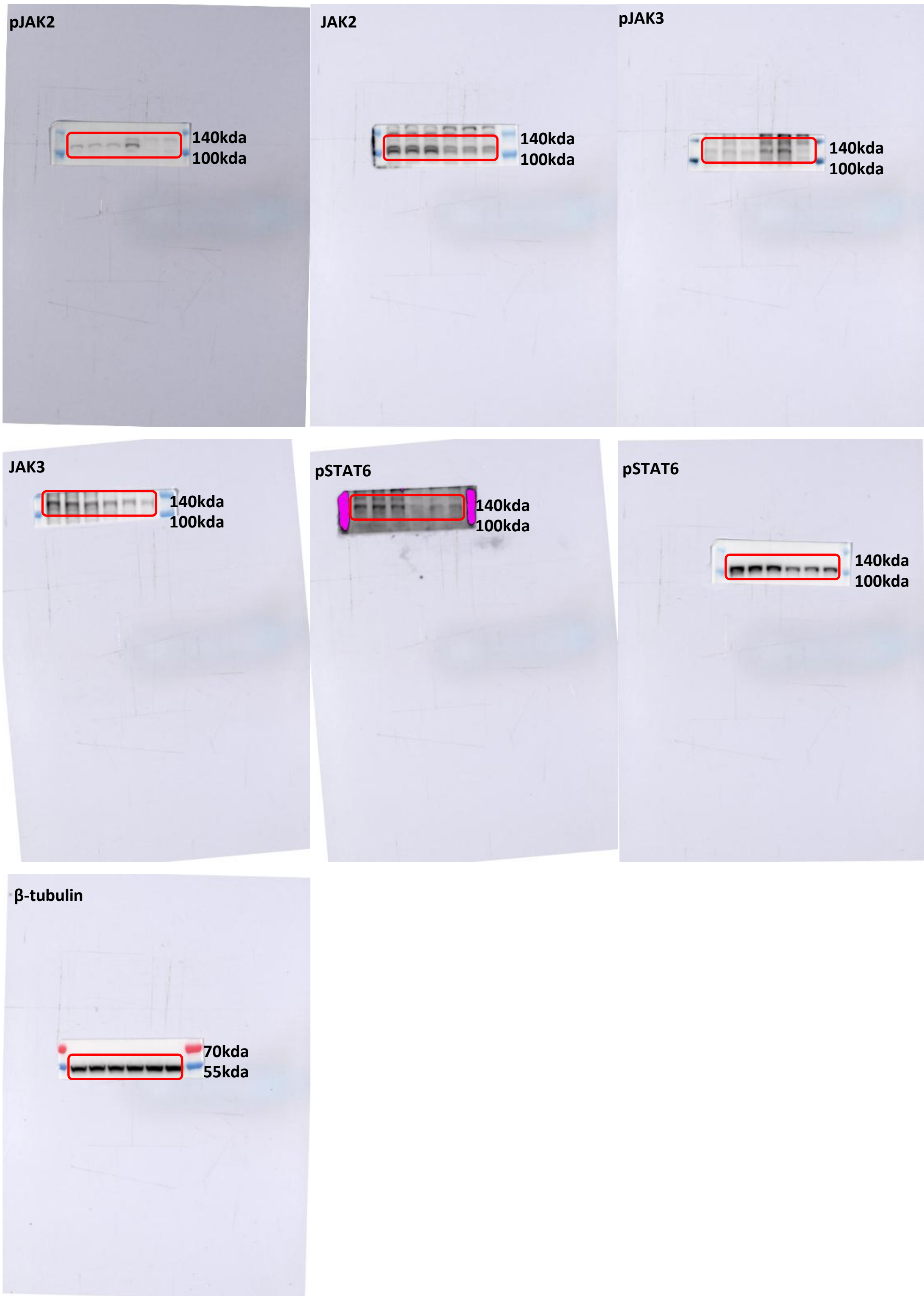

Corresponding to Fig. 6D

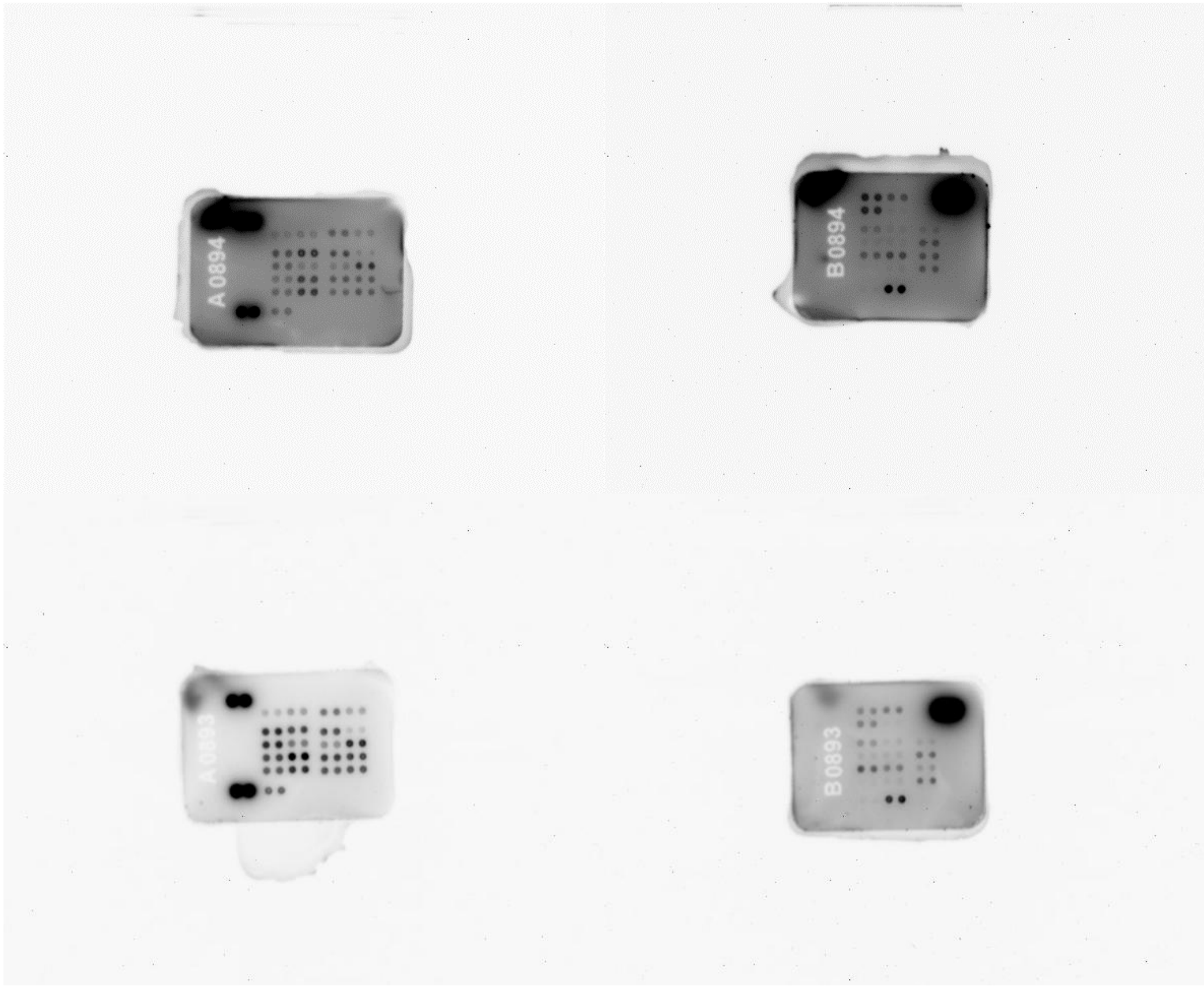

Corresponding to Fig. 6E

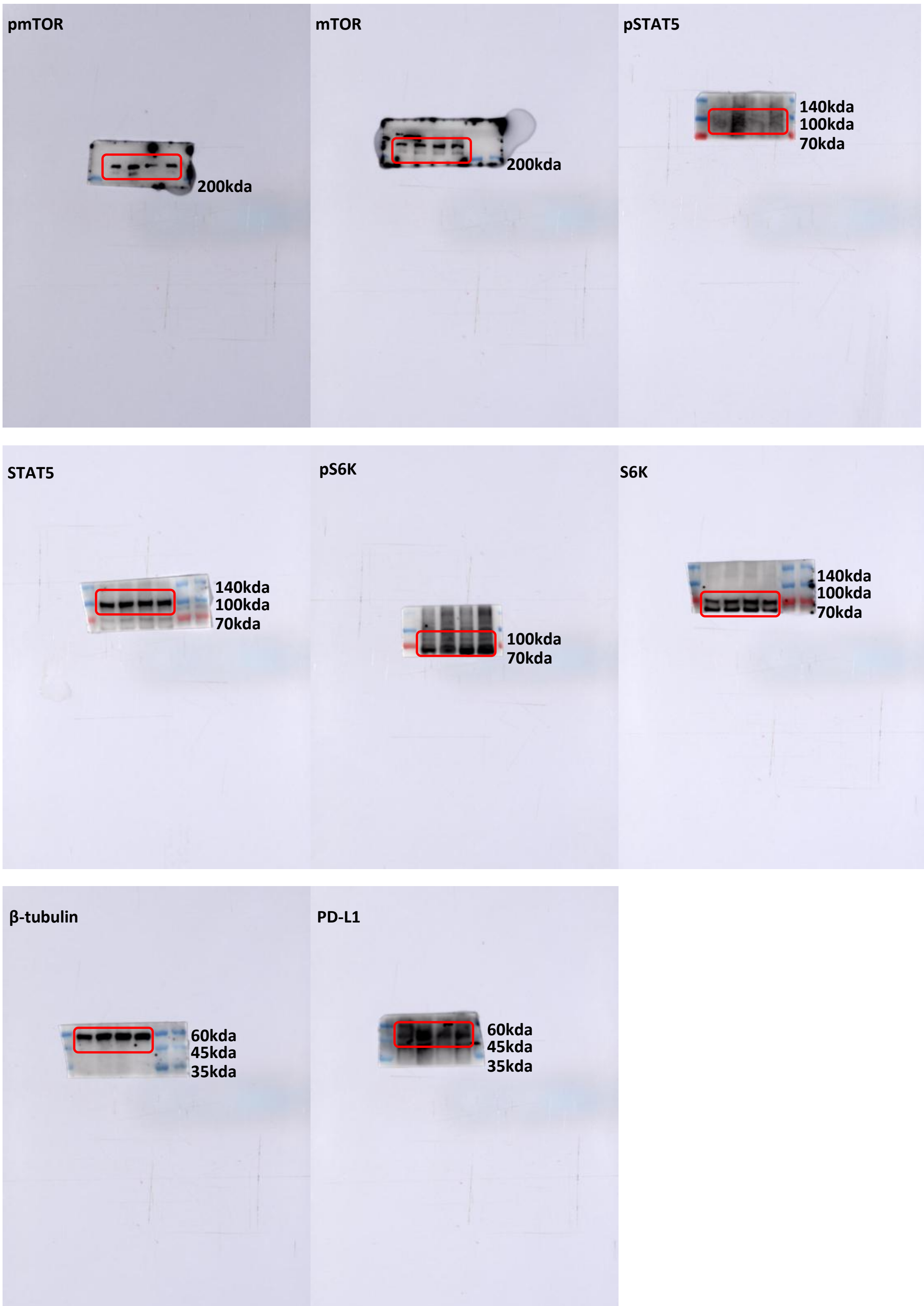

Corresponding to Fig. 6H

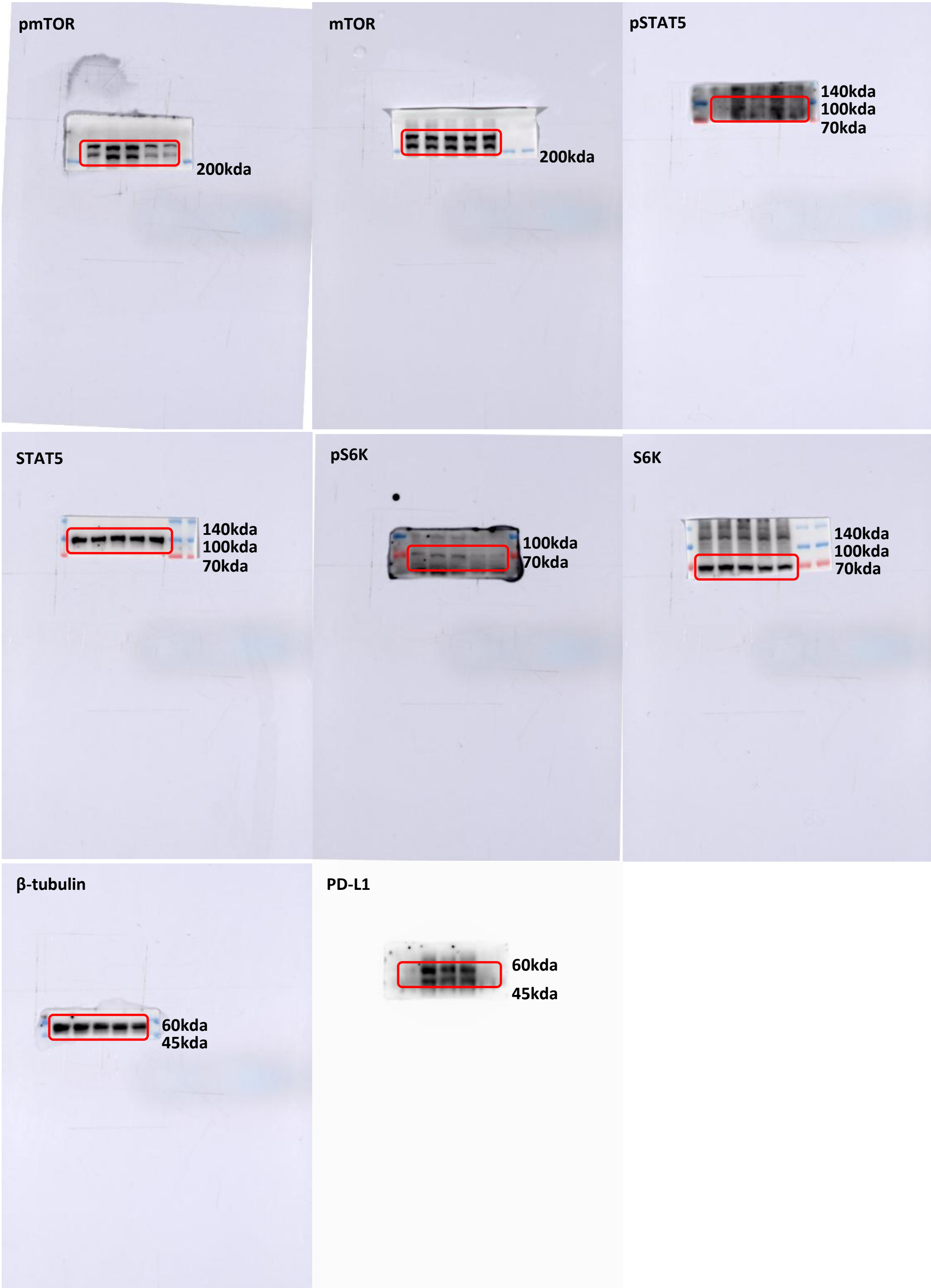

Supplement: Supplementary file 2 [file ADVS-13-e76960-s002.pdf]
