# Supplementary material for: GM‐CSF Promotes Neutrophil PD‐L1 Expression Through the VEGFR‐2/STAT6/CSF2 Signaling Axis in Oral Squamous Cell Carcinoma
Source: Adv Sci (Weinh). 2026 Aug 6;13(60):e76960. doi: 10.1002/advs.76960 (PMC13445316; doi:10.1002/advs.76960)
Supplement: Supplementary file 3 [file ADVS-13-e76960-s003.pdf]

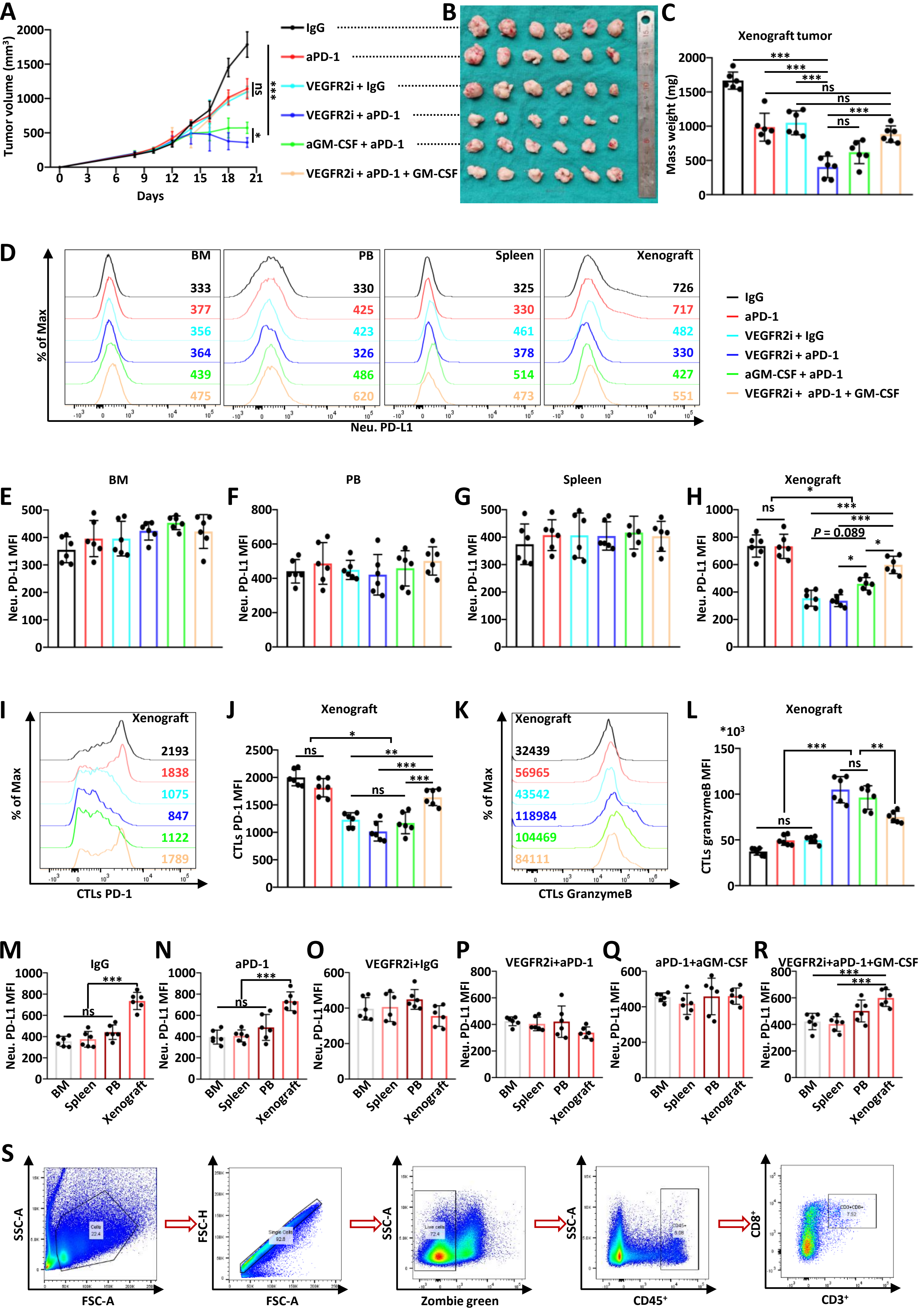
A
Tumor volume (mm3)
Days
IgG
aPD-1
VEGFR2i + IgG
VEGFR2i + aPD-1
aGM-CSF + aPD-1
VEGFR2i + aPD-1 + GM-CSF
ns
***
*
B
C
Xenograft tumor
Mass weight (mg)
D
BM
PB
Spleen
Xenograft
% of Max
Neu. PD-L1
333 377 356 364 439 475
330 425 423 326 486 620
325 330 461 378 514 473
726 717 482 330 427 551
E
BM
F
PB
G
Spleen
H
Xenograft
Neu. PD-L1 MFI
P = 0.089
I
Xenograft
2193 1838 1075 847 1122 1789
CTLs PD-1
J
Xenograft
CTLs PD-1 MFI
K
Xenograft
32439 56965 43542 118984 104469 84111
CTLs GranzymeB
L
Xenograft
*10³
CTLs granzymeB MFI
M
IgG
N
aPD-1
O
VEGFR2i+IgG
P
VEGFR2i+aPD-1
Q
aPD-1+aGM-CSF
R
VEGFR2i+aPD-1+GM-CSF
BM
Spleen
PB
Xenograft
S
SSC-A
FSC-A
FSC-H
Zombie green
CD45⁺
CD8⁺
CD3⁺
