# Supplementary material for: GM‐CSF Promotes Neutrophil PD‐L1 Expression Through the VEGFR‐2/STAT6/CSF2 Signaling Axis in Oral Squamous Cell Carcinoma
Source: Adv Sci (Weinh). 2026 Aug 6;13(60):e76960. doi: 10.1002/advs.76960 (PMC13445316; doi:10.1002/advs.76960)
Supplement: Supplementary file 4 [file ADVS-13-e76960-s006.pdf]

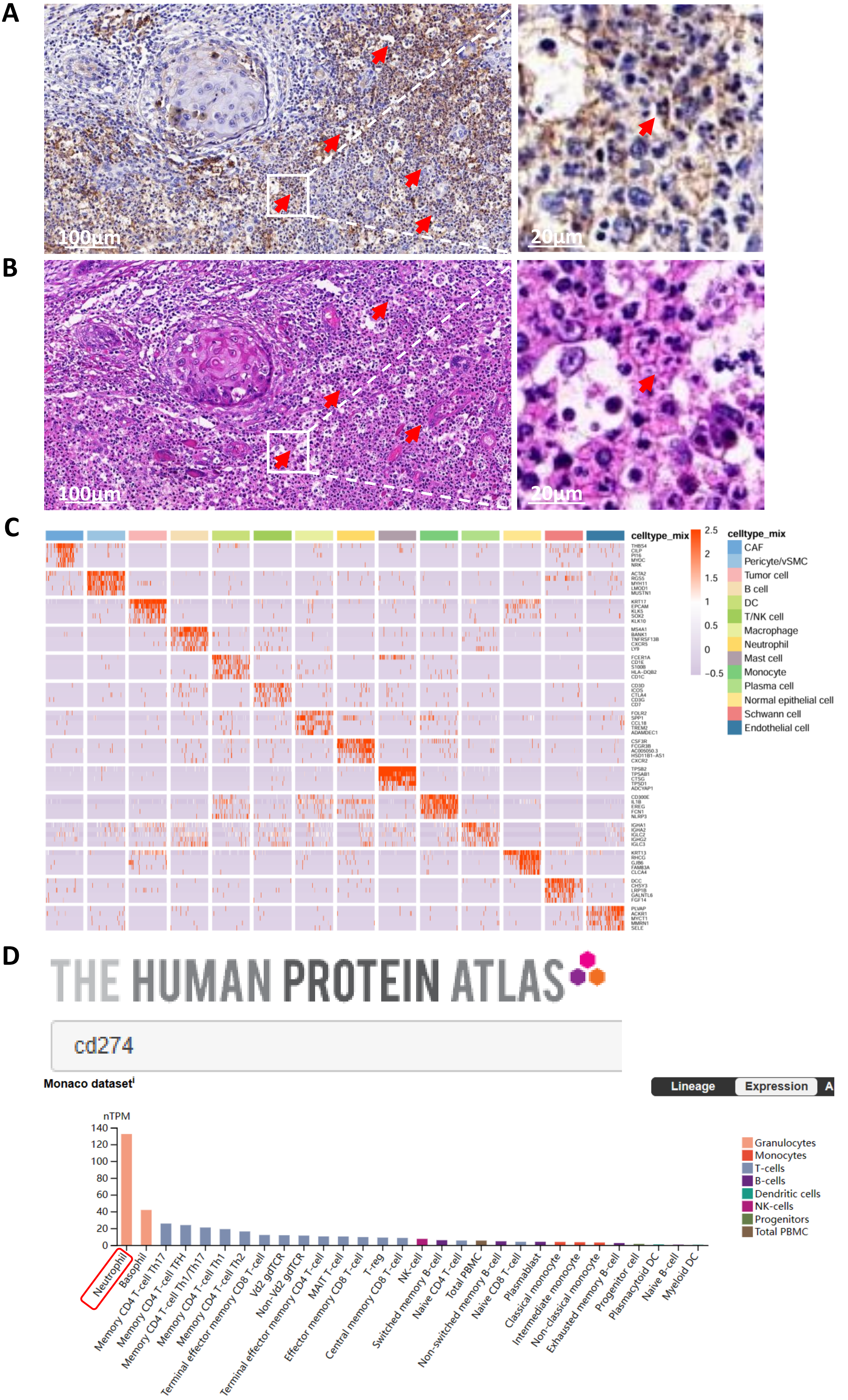
A
100μm
20μm
B
100μm
20μm
C
celltype_mix
2.5
2
1.5
1
0.5
0
−0.5
celltype_mix
CAF
Pericyte/vSMC
Tumor cell
B cell
DC
T/NK cell
Macrophage
Neutrophil
Mast cell
Monocyte
Plasma cell
Normal epithelial cell
Schwann cell
Endothelial cell
D
THE HUMAN PROTEIN ATLAS
cd274
Monaco dataset
Lineage
Expression
nTPM
140
120
100
80
60
40
20
0
Neutrophil
Basophil
Memory CD4 T-cell Th17
Memory CD4 T-cell TFH
Memory CD4 T-cell Th1/Th17
Memory CD4 T-cell Th1
Memory CD4 T-cell Th2
Terminal effector memory CD8 T-cell
Vd2 gdTCR
Non-Vd2 gdTCR
Terminal effector memory CD4 T-cell
MAIT T-cell
Effector memory CD8 T-cell
T-reg
Central memory CD8 T-cell
NK-cell
Switched memory B-cell
Naive CD4 T-cell
Total PBMC
Non-switched memory B-cell
Naive CD8 T-cell
Plasmablast
Classical monocyte
Intermediate monocyte
Non-classical monocyte
Exhausted memory B-cell
Progenitor cell
Plasmacytoid DC
Naive B-cell
Myeloid DC
Granulocytes
Monocytes
T-cells
B-cells
Dendritic cells
NK-cells
Progenitors
Total PBMC
