# Supplementary material for: GM‐CSF Promotes Neutrophil PD‐L1 Expression Through the VEGFR‐2/STAT6/CSF2 Signaling Axis in Oral Squamous Cell Carcinoma
Source: Adv Sci (Weinh). 2026 Aug 6;13(60):e76960. doi: 10.1002/advs.76960 (PMC13445316; doi:10.1002/advs.76960)
Supplement: Supplementary file 5 [file ADVS-13-e76960-s007.pdf]

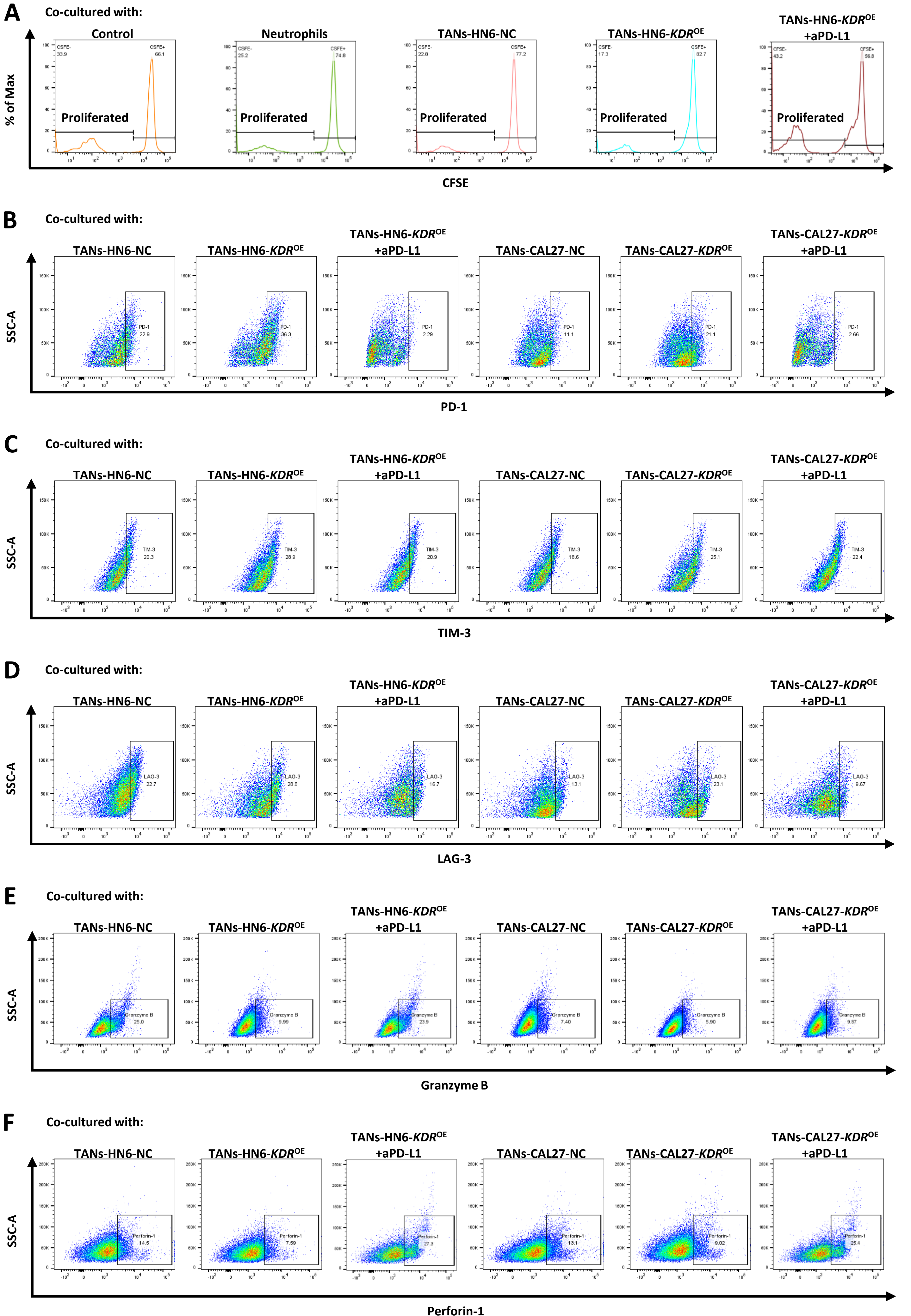
A
Co-cultured with:
Control
Neutrophils
TANs-HN6-NC
TANs-HN6-KDR^OE
TANs-HN6-KDR^OE +aPD-L1
% of Max
CSFE- 33.9
CSFE+ 66.1
CSFE- 25.2
CSFE+ 74.8
CSFE- 22.8
CSFE+ 77.2
CSFE- 17.3
CSFE+ 82.7
CFSE- 43.2
CFSE+ 56.8
Proliferated
CFSE
B
Co-cultured with:
TANs-HN6-NC
TANs-HN6-KDR^OE
TANs-HN6-KDR^OE +aPD-L1
TANs-CAL27-NC
TANs-CAL27-KDR^OE
TANs-CAL27-KDR^OE +aPD-L1
SSC-A
PD-1 22.9
PD-1 36.3
PD-1 2.29
PD-1 11.1
PD-1 21.1
PD-1 2.66
PD-1
C
Co-cultured with:
TANs-HN6-NC
TANs-HN6-KDR^OE
TANs-HN6-KDR^OE +aPD-L1
TANs-CAL27-NC
TANs-CAL27-KDR^OE
TANs-CAL27-KDR^OE +aPD-L1
SSC-A
TIM-3 20.3
TIM-3 28.9
TIM-3 20.9
TIM-3 18.6
TIM-3 25.1
TIM-3 22.4
TIM-3
D
Co-cultured with:
TANs-HN6-NC
TANs-HN6-KDR^OE
TANs-HN6-KDR^OE +aPD-L1
TANs-CAL27-NC
TANs-CAL27-KDR^OE
TANs-CAL27-KDR^OE +aPD-L1
SSC-A
LAG-3 22.7
LAG-3 28.8
LAG-3 16.7
LAG-3 13.1
LAG-3 23.1
LAG-3 9.67
LAG-3
E
Co-cultured with:
TANs-HN6-NC
TANs-HN6-KDR^OE
TANs-HN6-KDR^OE +aPD-L1
TANs-CAL27-NC
TANs-CAL27-KDR^OE
TANs-CAL27-KDR^OE +aPD-L1
SSC-A
Granzyme B 25.0
Granzyme B 9.99
Granzyme B 23.9
Granzyme B 7.40
Granzyme B 5.90
Granzyme B 9.87
Granzyme B
F
Co-cultured with:
TANs-HN6-NC
TANs-HN6-KDR^OE
TANs-HN6-KDR^OE +aPD-L1
TANs-CAL27-NC
TANs-CAL27-KDR^OE
TANs-CAL27-KDR^OE +aPD-L1
SSC-A
Perforin-1 14.5
Perforin-1 7.59
Perforin-1 27.3
Perforin-1 13.1
Perforin-1 9.02
Perforin-1 25.4
Perforin-1
