# Supplementary material for: GM‐CSF Promotes Neutrophil PD‐L1 Expression Through the VEGFR‐2/STAT6/CSF2 Signaling Axis in Oral Squamous Cell Carcinoma
Source: Adv Sci (Weinh). 2026 Aug 6;13(60):e76960. doi: 10.1002/advs.76960 (PMC13445316; doi:10.1002/advs.76960)
Supplement: Supplementary file 6 [file ADVS-13-e76960-s009.pdf]

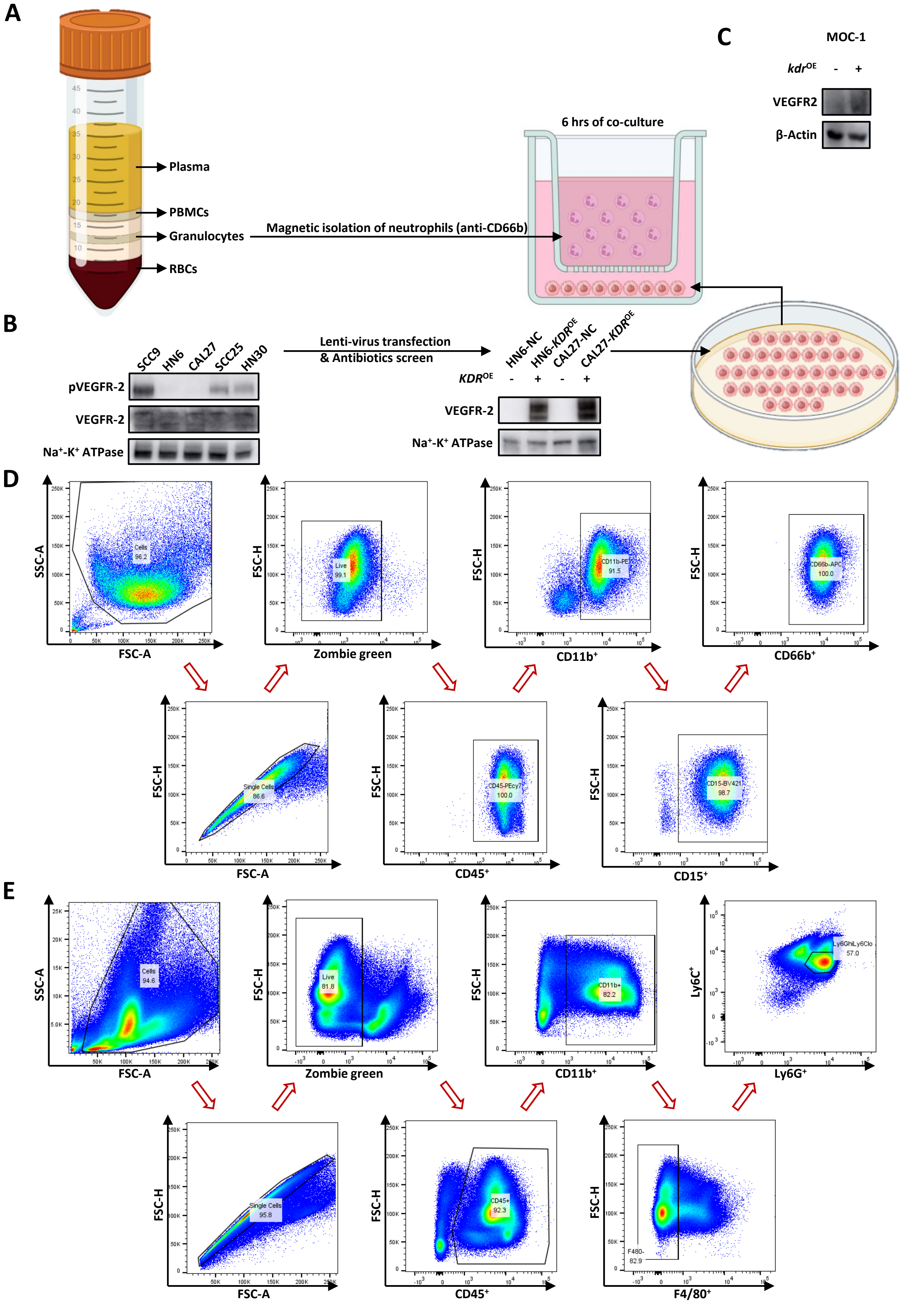

A
Plasma
PBMCs
Granulocytes
RBCs
Magnetic isolation of neutrophils (anti-CD66b)
6 hrs of co-culture
B
SCC9
HN6
CAL27
SCC25
HN30
pVEGFR-2
VEGFR-2
Na+-K+ ATPase
Lenti-virus transfection
& Antibiotics screen
HN6-NC
HN6-KDROE
CAL27-NC
CAL27-KDROE
KDROE
VEGFR-2
Na+-K+ ATPase
C
MOC-1
kdrOE
VEGFR2
β-Actin
D
SSC-A
FSC-A
Cells 96.2
FSC-H
Zombie green
Live 99.1
CD11b+
CD11b-PE 91.5
CD66b+
CD66b-APC 100.0
Single Cells 86.6
CD45+
CD45-PEcy7 100.0
CD15+
CD15-BV421 98.7
E
Cells 94.6
Live 81.8
CD11b+ 82.2
Ly6C+
Ly6G+
Ly6GhiLy6Clo 57.0
Single Cells 95.8
CD45+ 92.3
F4/80+
F480- 82.9
