# Supplementary material for: GM‐CSF Promotes Neutrophil PD‐L1 Expression Through the VEGFR‐2/STAT6/CSF2 Signaling Axis in Oral Squamous Cell Carcinoma
Source: Adv Sci (Weinh). 2026 Aug 6;13(60):e76960. doi: 10.1002/advs.76960 (PMC13445316; doi:10.1002/advs.76960)
Supplement: Supplementary file 7 [file ADVS-13-e76960-s012.pdf]

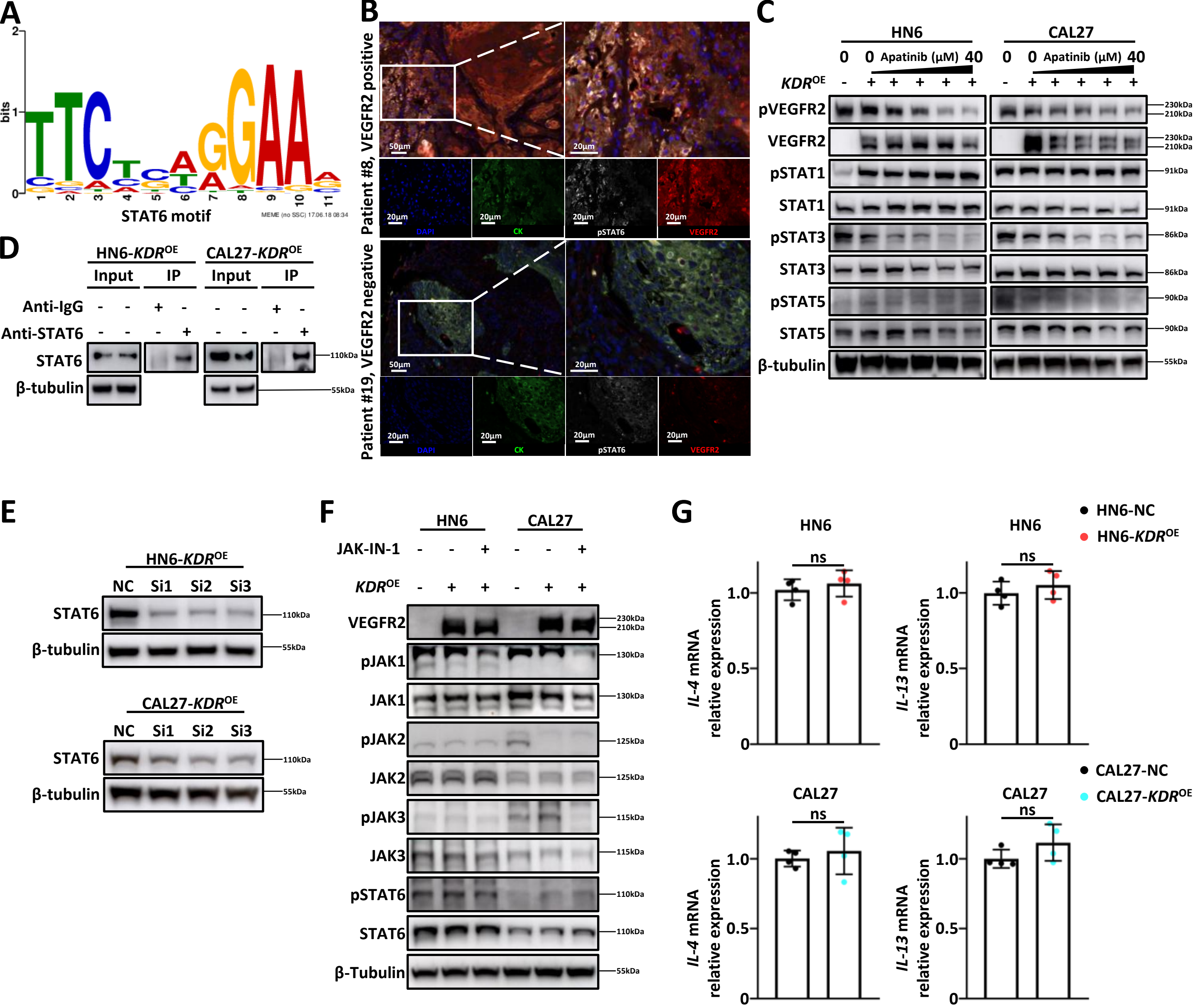
A
STAT6 motif
MEME (no SSC) 17.06.18 08:34
B
Patient #8, VEGFR2 positive
Patient #19, VEGFR2 negative
DAPI
CK
pSTAT6
VEGFR2
C
HN6
CAL27
Apatinib (μM)
KDR^OE
pVEGFR2
VEGFR2
pSTAT1
STAT1
pSTAT3
STAT3
pSTAT5
STAT5
β-tubulin
D
HN6-KDR^OE
CAL27-KDR^OE
Input
IP
Anti-IgG
Anti-STAT6
STAT6
β-tubulin
E
HN6-KDR^OE
CAL27-KDR^OE
NC
Si1
Si2
Si3
STAT6
β-tubulin
F
HN6
CAL27
JAK-IN-1
KDR^OE
VEGFR2
pJAK1
JAK1
pJAK2
JAK2
pJAK3
JAK3
pSTAT6
STAT6
β-Tubulin
G
HN6
CAL27
HN6-NC
HN6-KDR^OE
CAL27-NC
CAL27-KDR^OE
ns
IL-4 mRNA relative expression
IL-13 mRNA relative expression
