# Supplementary material for: GM‐CSF Promotes Neutrophil PD‐L1 Expression Through the VEGFR‐2/STAT6/CSF2 Signaling Axis in Oral Squamous Cell Carcinoma
Source: Adv Sci (Weinh). 2026 Aug 6;13(60):e76960. doi: 10.1002/advs.76960 (PMC13445316; doi:10.1002/advs.76960)
Supplement: Supplementary file 8 [file ADVS-13-e76960-s013.pdf]

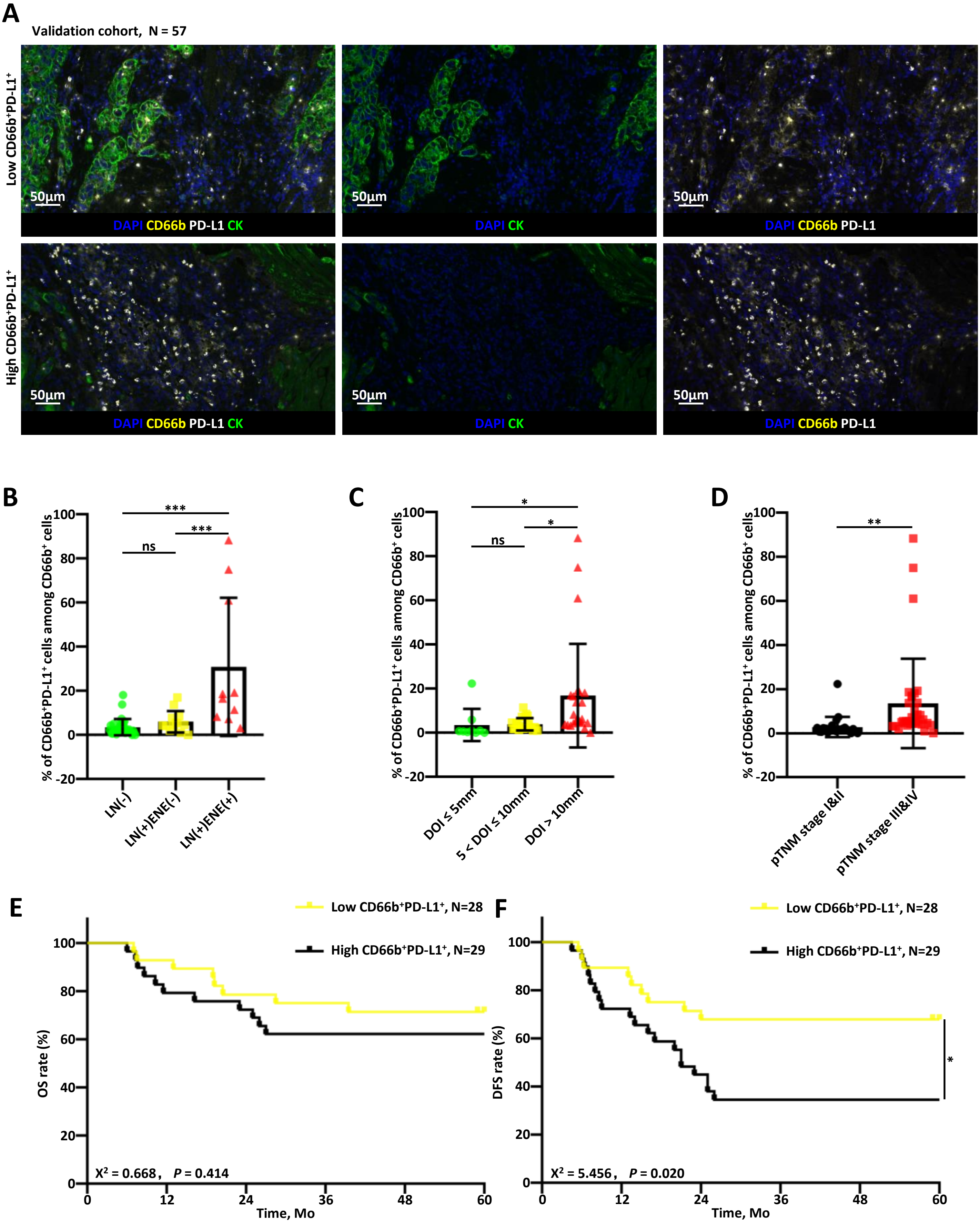
A
Validation cohort, N = 57
Low CD66b$^+$PD-L1$^+$
High CD66b$^+$PD-L1$^+$
50μm
DAPI CD66b PD-L1 CK
DAPI CK
DAPI CD66b PD-L1
B
C
D
% of CD66b$^+$PD-L1$^+$ cells among CD66b$^+$ cells
ns
***
*
**
LN(-)
LN(+)ENE(-)
LN(+)ENE(+)
DOI ≤ 5mm
5 < DOI ≤ 10mm
DOI > 10mm
pTNM stage I&II
pTNM stage III&IV
E
F
Low CD66b$^+$PD-L1$^+$, N=28
High CD66b$^+$PD-L1$^+$, N=29
OS rate (%)
DFS rate (%)
Time, Mo
X^2 = 0.668， P = 0.414
X^2 = 5.456， P = 0.020
