# Supplementary material for: GM‐CSF Promotes Neutrophil PD‐L1 Expression Through the VEGFR‐2/STAT6/CSF2 Signaling Axis in Oral Squamous Cell Carcinoma
Source: Adv Sci (Weinh). 2026 Aug 6;13(60):e76960. doi: 10.1002/advs.76960 (PMC13445316; doi:10.1002/advs.76960)
Supplement: Supplementary file 9 [file ADVS-13-e76960-s016.pdf]

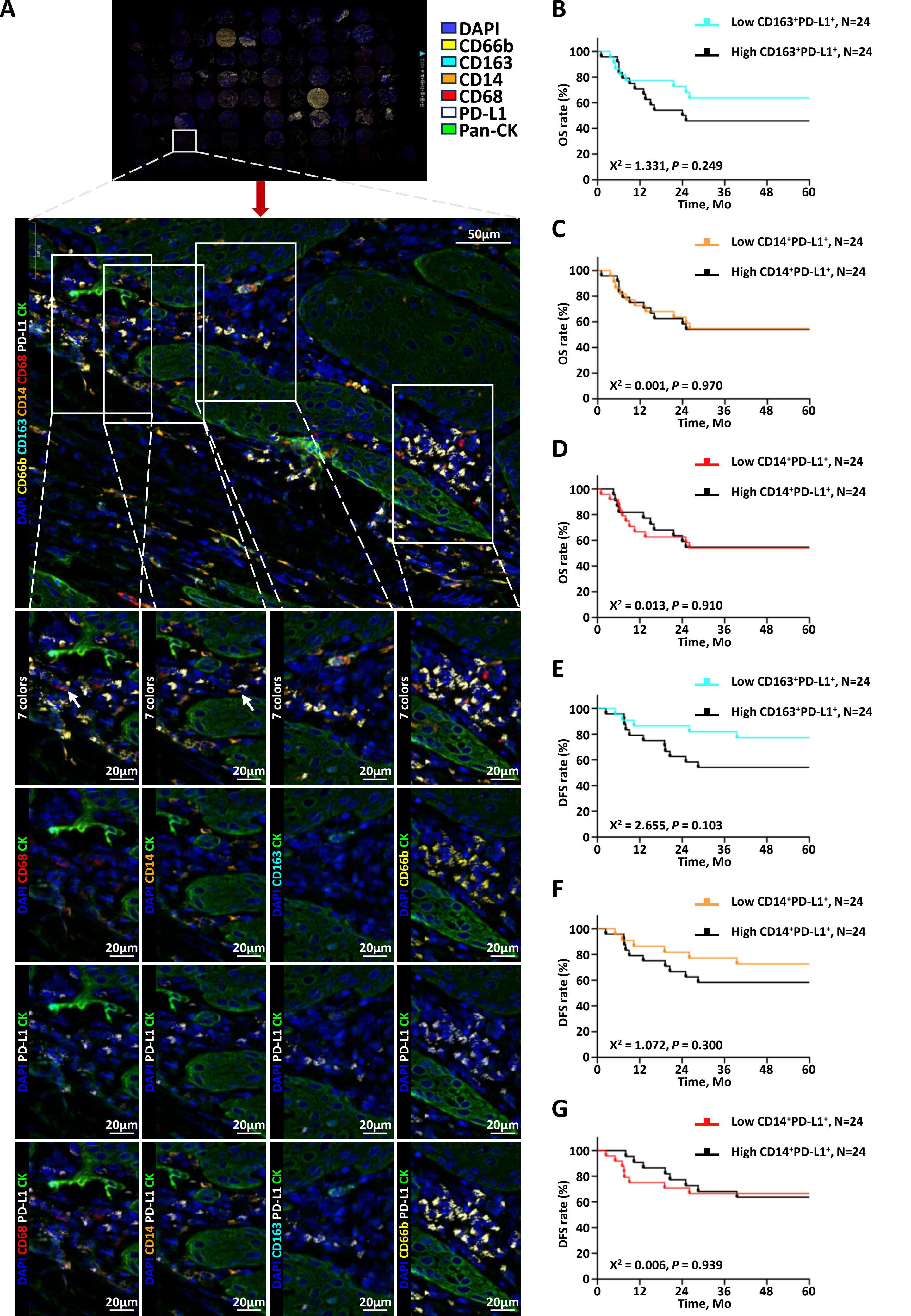
A
DAPI
CD66b
CD163
CD14
CD68
PD-L1
Pan-CK
50µm
DAPI CD66b CD163 CD14 CD68 PD-L1 CK
7 colors
20µm
DAPI CD68 CK
DAPI CD14 CK
DAPI CD163 CK
DAPI CD66b CK
DAPI PD-L1 CK
DAPI CD68 PD-L1 CK
DAPI CD14 PD-L1 CK
DAPI CD163 PD-L1 CK
DAPI CD66b PD-L1 CK
B
Low CD163$^+$PD-L1$^+$, N=24
High CD163$^+$PD-L1$^+$, N=24
OS rate (%)
X^2 = 1.331, P = 0.249
Time, Mo
C
Low CD14$^+$PD-L1$^+$, N=24
High CD14$^+$PD-L1$^+$, N=24
OS rate (%)
X^2 = 0.001, P = 0.970
Time, Mo
D
Low CD14$^+$PD-L1$^+$, N=24
High CD14$^+$PD-L1$^+$, N=24
OS rate (%)
X^2 = 0.013, P = 0.910
Time, Mo
E
Low CD163$^+$PD-L1$^+$, N=24
High CD163$^+$PD-L1$^+$, N=24
DFS rate (%)
X^2 = 2.655, P = 0.103
Time, Mo
F
Low CD14$^+$PD-L1$^+$, N=24
High CD14$^+$PD-L1$^+$, N=24
DFS rate (%)
X^2 = 1.072, P = 0.300
Time, Mo
G
Low CD14$^+$PD-L1$^+$, N=24
High CD14$^+$PD-L1$^+$, N=24
DFS rate (%)
X^2 = 0.006, P = 0.939
Time, Mo
