# Supplementary material for: GM‐CSF Promotes Neutrophil PD‐L1 Expression Through the VEGFR‐2/STAT6/CSF2 Signaling Axis in Oral Squamous Cell Carcinoma
Source: Adv Sci (Weinh). 2026 Aug 6;13(60):e76960. doi: 10.1002/advs.76960 (PMC13445316; doi:10.1002/advs.76960)
Supplement: Supplementary file 10 [file ADVS-13-e76960-s018.pdf]

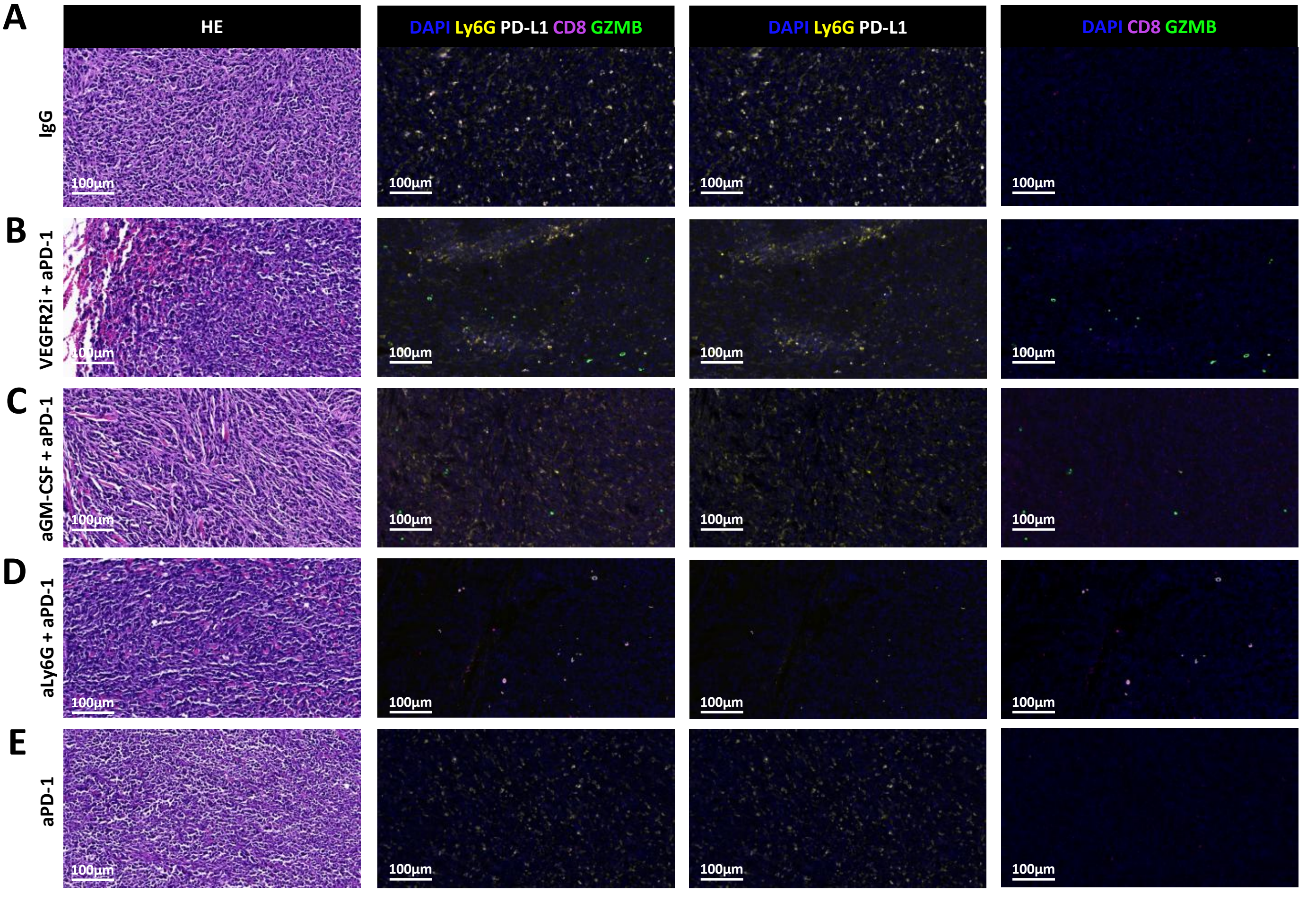
A
B
C
D
E
HE
DAPI Ly6G PD-L1 CD8 GZMB
DAPI Ly6G PD-L1
DAPI CD8 GZMB
IgG
VEGFR2i + aPD-1
aGM-CSF + aPD-1
aLy6G + aPD-1
aPD-1
100μm
