# Supplementary material for: GM‐CSF Promotes Neutrophil PD‐L1 Expression Through the VEGFR‐2/STAT6/CSF2 Signaling Axis in Oral Squamous Cell Carcinoma
Source: Adv Sci (Weinh). 2026 Aug 6;13(60):e76960. doi: 10.1002/advs.76960 (PMC13445316; doi:10.1002/advs.76960)
Supplement: Supplementary file 11 [file ADVS-13-e76960-s020.pdf]

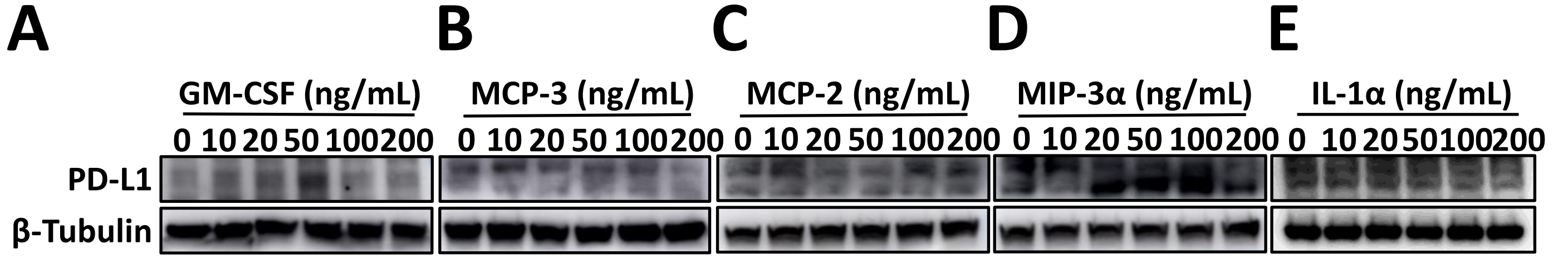
A
B
C
D
E
GM-CSF (ng/mL)
0 10 20 50 100 200
MCP-3 (ng/mL)
0 10 20 50 100 200
MCP-2 (ng/mL)
0 10 20 50 100 200
MIP-3α (ng/mL)
0 10 20 50 100 200
IL-1α (ng/mL)
0 10 20 50 100 200
PD-L1
β-Tubulin
