# Supplementary material for: GM‐CSF Promotes Neutrophil PD‐L1 Expression Through the VEGFR‐2/STAT6/CSF2 Signaling Axis in Oral Squamous Cell Carcinoma
Source: Adv Sci (Weinh). 2026 Aug 6;13(60):e76960. doi: 10.1002/advs.76960 (PMC13445316; doi:10.1002/advs.76960)
Supplement: Supplementary file 12 [file ADVS-13-e76960-s017.docx]

**Supplementary Fig. S1 PD-L1 is expressed on cells out of CPS counting cells in the micro-environment of OSCC patients.**

A. PD-L1 immunohistochemical staining of an OSCC patient sample, with red arrows indicating PD-L1^+^ non-CPS-counted cells;
B. Serial hematoxylin-eosin-stained sections of the OSCC patient sample, with red arrows indicating PD-L1^+^ neutrophils;
C. Heatmap showing expression of known markers across cell clusters;
D. *CD274* expression among immune cells from the human protein atlas.

**Supplementary Fig. S2. mIHC staining and Kaplan-Meier analysis of patients with different PD-L1^+^ cells of the 48 OSCC patients’ cohort.**

A. Representative image of mIHC staining of TMA with a cohort of 48 OSCC patients, showing PD-L1 staining on different cells types. White arrows showing CD14^+^CD66b^+^PD-L1^+^ and CD68^+^CD66b^+^PD-L1^+^ cells;
B. Kaplan-Meier analysis of OS based on the CD163^+^PD-L1^+^ cells level in 48 OSCC patients;
C. Kaplan-Meier analysis of DFS based on the CD163^+^PD-L1^+^ cells level in 48 OSCC patients;
D. Kaplan-Meier analysis of OS based on the CD14^+^PD-L1^+^ cells level in 48 OSCC patients;
E. Kaplan-Meier analysis of DFS based on the CD14^+^PD-L1^+^ cells level in 48 OSCC patients;
F. Kaplan-Meier analysis of OS based on the CD68^+^PD-L1^+^ cells level in 48 OSCC patients;
G. Kaplan-Meier analysis of DFS based on the CD68^+^PD-L1^+^ cells level in 48 OSCC patients;

*: *P*<0.05; OS: overall survival; DFS: disease-free survival.

**Supplementary Fig. S3. High CD66b^+^PD-L1^+^ cell density is associated with more aggressive clinicopathological features validated in the 57 OSCC patients’ cohort.**

A. Representative image of mIHC staining of the 57 OSCC patients’ cohort, showing patients with low CD66b^+^PD-L1^+^ and high CD66b^+^PD-L1^+^ cells;
B. Percentage of CD66b^+^PD-L1^+^ cells in patients without lymph node metastasis (LN-MT), with LN-MT but without extranodal extension (ENE), or with LN-MT and ENE;
C. Percentage of CD66b^+^PD-L1^+^ cells of patients with different depth of invasion (DOI);
D. Percentage of CD66b^+^PD-L1^+^ cells of patients with different pathological TNM (pTNM) stage;
E. Kaplan-Meier analysis of overall survival (OS) based on the CD66b^+^PD-L1^+^ cells level in 57 OSCC patients;
F. Kaplan-Meier analysis of disease-free survival (DFS) based on the CD66b^+^PD-L1^+^ cells level in 57 OSCC patients.

ns: non-significant; *: *P*<0.05; **: *P*<0.01; ***: *P*<0.001; mIHC: multiplex-immunohistochemical; LN-MT: lymph node metastasis; ENE: extra-node extension; pTNM: pathological TNM; OS: overall survival; DFS: disease-free survival.

**Supplementary Fig. S4. *KDR* expression and immune cell infiltration.**

A. *KDR* expression difference in tumor and the paired normal tissue of different cancer types from TCGA database;
B. *KDR* expression and neutrophil infiltration from head and neck squamous cell carcinoma patients from TCGA database;

**Supplementary Fig. S5.**

A. Schematic illustration of neutrophil isolation and coculture with OSCC cell lines;
B. VEGFR-2 expression in different cell lines detected by western blotting, and VEGFR-2 expression after lentiviral infection and antibiotic selection;
C. VEGFR-2 expression in C57BL/6 OSCC cell line MOC-1 detected by western blotting after lenti-virus infection and antibiotics screen;
D. Gating strategy of human neutrophils;
E. Gating strategy of mouse neutrophils.

**Supplementary Fig. S6. Neutrophil PD-L1 expression after stimulation with different cytokines/chemokines.**

A. Western blot detection of PD-L1 expression in neutrophils cultured with different concentrations of GM-CSF;
B. Western blot detection of PD-L1 expression in neutrophils cultured with different concentrations of MCP-3;
C. Western blot detection of PD-L1 expression in neutrophils cultured with different concentrations of MCP-2;
D. Western blot detection of PD-L1 expression in neutrophils cultured with different concentrations of MIP-3α;
E. Western blot detection of PD-L1 expression in neutrophils cultured with different concentrations of IL-1α.

**Supplementary Fig. S7. STAT6 phosphorylation is not mainly induced by JAK or IL-4/IL13 signaling pathway in VEGFR-2 overexpression OSCC cell lines.**

A. Transcription factor prediction by database hTFtarget and JASPAR;
B. mIHC staining showing that pSTAT6 positively correlates with VEGFR-2 expression. Upper: VEGFR-2-positive; lower: VEGFR-2-negative;
C. Western blotting detection of VEGFR-2, STAT1, STAT3, STAT5 in HN6-NC or HN6-*KDR*^OE^, and CAL27-NC or CAL27-*KDR*^OE^, treated with VEGFR-2 inhibitor, apatinib;
D. Chromatin immunoprecipitation (ChIP) with anti-pSTAT6 and western blotting detection;
E. Western blotting was used to assess the knockdown efficiency of Si-STAT6 in HN6-*KDR*^OE^ and CAL27-*KDR*^OE^;
F. Western blot assessment of VEGFR-2 overexpression, STAT6 phosphorylation, and JAK kinase phosphorylation;
G. RT-qPCR showing VEGFR-2 overexpression and *IL-4*/*IL-13* mRNA expression.

**Supplementary Fig. S8. CTLs proliferation and cytotoxic function are suppressed by OSCC-KDROE-derived TANs.**

A. Flow cytometry showing CFSE-labeled CTL proliferation after coculture with different cells;
B. Flow cytometry showing CTL PD-1 expression after coculture with different cells;
C. Flow cytometry showing CTL TIM-3 expression after coculture with different cells;
D. Flow cytometry showing CTL LAG-3 expression after coculture with different cells;
E. Flow cytometry showing CTL granzyme B expression after coculture with different cells;
F. Flow cytometry showing CTL perforin 1 expression after coculture with different cells;

All cells in this figure were gated on single cells, live cells, CD45^+^ cells, CD3^+^ cells, CD8^+^ T lymphocytes.

**Supplementary Fig. S9. VEGFR2 inhibition enhances anti-PD-1 efficacy by suppressing GM-CSF-dependent TANs PD-L1 expression and restoring CTLs function in MOC1-*kdr*^OE^ subcutaneous xenografts**

A. Tumor growth of MOC1-*kdr*^OE^ subcutaneous xenografts treated with the drug combinations;
B. Photo of the subcutaneous xenografts;
C. Tumor weight at day 20;
D. Flow cytometry detection of neutrophil PD-L1 expression in bone marrow, spleen, peripheral blood, and xenografts;
E. Quantitative flow cytometry analysis of PD-L1 MFI in bone marrow-derived neutrophils from tumor-bearing mice;
F. Quantitative flow cytometry analysis of PD-L1 MFI in peripheral blood-derived neutrophils from tumor-bearing mice;
G. Quantitative flow cytometry analysis of PD-L1 MFI in spleen-derived neutrophils from tumor-bearing mice;
H. Quantitative flow cytometry analysis of PD-L1 MFI in xenograft-derived neutrophils (TANs) from tumor-bearing mice;
I. Flow cytometry detection of PD-1 expression in xenograft-derived CTLs;
J. Quantitative flow cytometry analysis of PD-1 MFI in xenograft-derived CTLs from tumor-bearing mice;
K. Flow cytometry detecting CTLs granzyme B expression in xenograft;
L. Quantitative flow cytometry analysis of granzyme B MFI in xenograft-derived CTLs from tumor-bearing mice;
M. Statistics of neutrophil PD-L1 MFI from different organs in MOC1-*kdr*^OE^ xenograft-bearing mice treated with IgG;
N. Statistics of neutrophil PD-L1 MFI from different organs in MOC1-*kdr*^OE^ xenograft-bearing mice treated with aPD-1;
O. Statistics of neutrophil PD-L1 MFI from different organs in MOC1-*kdr*^OE^ xenograft-bearing mice treated with VEGFR2i + IgG;
P. Statistics of neutrophil PD-L1 MFI from different organs in MOC1-*kdr*^OE^ xenograft-bearing mice treated with VEGFR2i + aPD-1;
Q. Statistics of neutrophil PD-L1 MFI from different organs in MOC1-*kdr*^OE^ xenograft-bearing mice treated with VEGFR2i + aGM-CSF;
R. Statistics of neutrophil PD-L1 MFI from different organs in MOC1-*kdr*^OE^ xenograft-bearing mice treated with VEGFR2i + aPD-1 + GM-CSF;
S. Gating strategy of CTLs in mice.

BM: bone marrow; PB: peripheral blood; Neu.: neutrophils; CTLs: cytotoxic T lymphocytes; aPD-1: PD-1 antibody; aGM-CSF: GM-CSF neutralizing antibody; VEGFR2i: VEGFR-2 inhibitor.

**Supplementary Fig. S10. Representative H&E and mIHC images of orthotopic tongue tumors from different treatment groups.**

A. Representative HE and mIHC staining of orthotopic tongue tumors from the IgG group;
B. Representative HE and mIHC staining of orthotopic tongue tumors from the VEGFR2i + aPD-1 group;
C. Representative HE and mIHC staining of orthotopic tongue tumors from the aGM-CSF + aPD-1 group;
D. Representative HE and mIHC staining of orthotopic tongue tumors from the aLy6G + aPD-1 group;
E. Representative HE and mIHC staining of orthotopic tongue tumors from the aPD-1 group;

**Supplementary Fig. S11. Raw and unedited immunoblot images.**
